# The *Calcidiscus leptoporus* genome reveals vitamin-mediated holobiont interactions

**DOI:** 10.1093/ismeco/ycag222

**Published:** 2026-08-23

**Authors:** Frederic Chaux, Tanja Vojvoda Zeljko, Borna Branimir Vuković, John A Burns, Titouan Le Perrun, Marta Žižek, Briget P Bannerman, Donald Tonye Bara Mason, Clotilde Garrido, Zhou Xu, Richard G Dorrell, Jelena Godrijan

**Affiliations:** Division for Marine and Environmental Research, Ruđer Bošković Institute, 10000 Zagreb, Croatia; Division for Marine and Environmental Research, Ruđer Bošković Institute, 10000 Zagreb, Croatia; Division for Marine and Environmental Research, Ruđer Bošković Institute, 10000 Zagreb, Croatia; Bigelow Laboratory for Ocean Sciences, East Boothbay, ME 04544, United States; Division for Marine and Environmental Research, Ruđer Bošković Institute, 10000 Zagreb, Croatia; Division for Marine and Environmental Research, Ruđer Bošković Institute, 10000 Zagreb, Croatia; Faculty of Clinical Medicine, University of Cambridge, Cambridge CB2 0SP, United Kingdom; Faculty of Clinical Medicine, University of Cambridge, Cambridge CB2 0SP, United Kingdom; Department of Computer Science, Department of Computer Science, University of the People, Pasadena, CA 91101, United States; Computational, Quantitative and Synthetic Biology (CQSB), Institut de Biologie Paris-Seine (IBPS), CNRS, INSERM, Sorbonne Université, 75005 Paris, France; Computational, Quantitative and Synthetic Biology (CQSB), Institut de Biologie Paris-Seine (IBPS), CNRS, INSERM, Sorbonne Université, 75005 Paris, France; Faculty of Clinical Medicine, University of Cambridge, Cambridge CB2 0SP, United Kingdom; Division for Marine and Environmental Research, Ruđer Bošković Institute, 10000 Zagreb, Croatia

**Keywords:** *Calcidiscus leptoporus,* coccolithophore genome, genome-resolved metagenomics, holobiont metabolism complementarity, phycosphere, B-vitamin biosynthesis, vitamin cross-feeding, microbial community assembly, host–microbe interactions, microbiome succession

## Abstract

Coccolithophores are major marine phytoplankton that contribute to ocean carbon cycling through both organic carbon fixation and calcium carbonate biomineralization, yet the functional basis of their interactions with phycosphere bacteria remains poorly resolved. Here, we present the nuclear genome of the haploid phase of the coccolithophore *Calcidiscus leptoporus*, a coccolithophore that calcifies in both life-cycle stages. We combined host genome analysis, genome-resolved characterization of associated bacteria, and a four-month vitamin-manipulation experiment to test how B-vitamin biosynthetic complementarity relates to host performance and phycosphere community assembly. Metabolic reconstructions indicate partitioned B-vitamin biosynthtic potential: the host encodes pathways for B2, B5, B6, and B9, including a rare fused B5 biosynthesis gene, but lacks complete pathways for B1, B3, B7, and B12. These missing functions were distributed among recurrent bacterial taxa, with no single bacterial MAG encoding the full set of host-missing vitamins. Across the experiment, bacterial community composition was structured primarily by the experimental phase, while vitamin treatments secondarily influenced which taxa became enriched at later stages. Consistent with genome-inferred auxotrophy, B-vitamin availability constrained long-term growth under the tested conditions, with B1 alone providing partial rescue and vitamin-replete treatments showing equal or stronger responses. Together, these data establish *C. leptoporus* as a genomic model for coccolithophore biology and holobiont interactions, while providing a testable framework linking vitamin economies to phycosphere assembly and host performance.

## Graphical abstract

**Figure:**
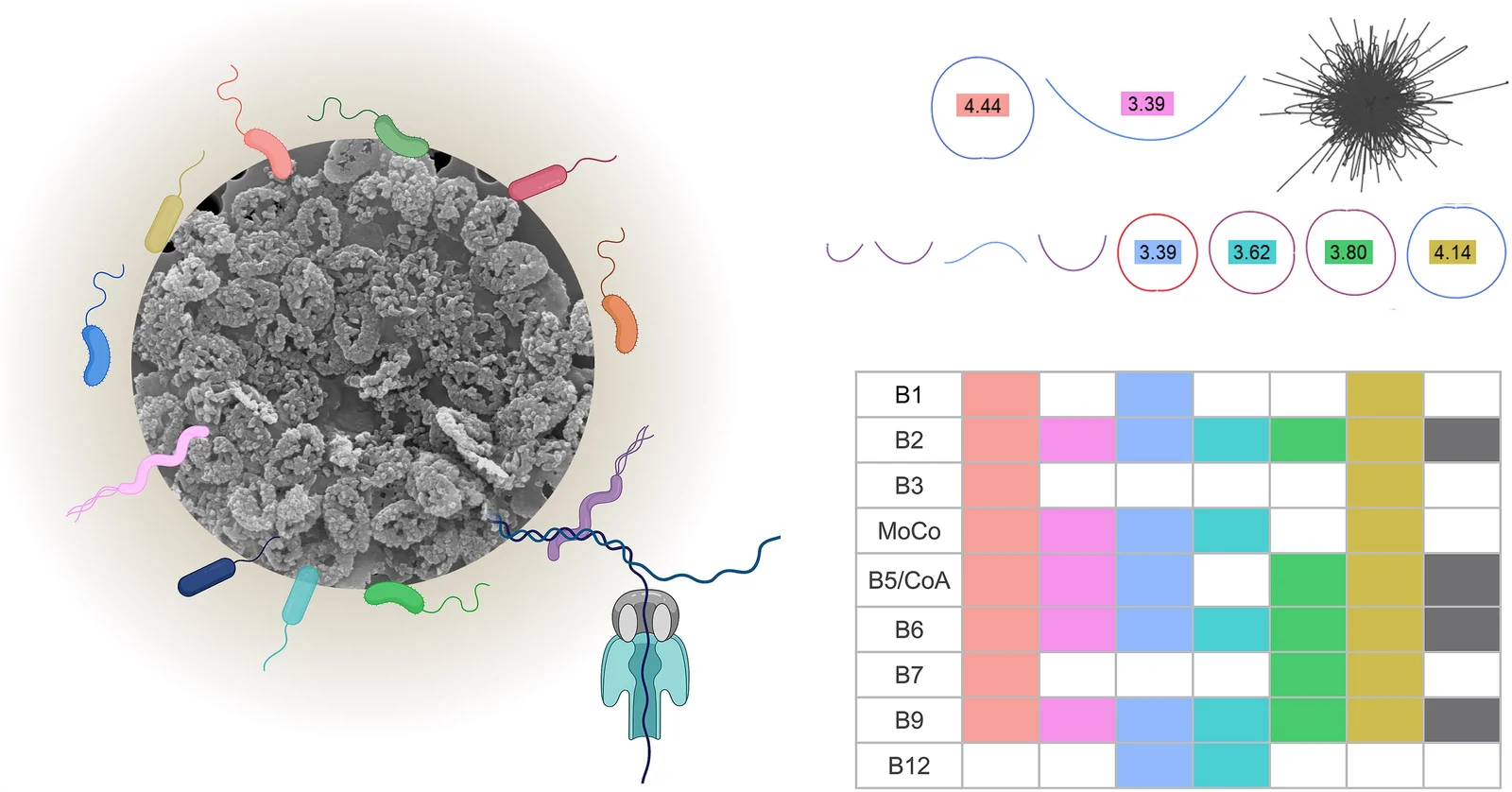

## Introduction

The ocean harbors diverse microbial life that regulates biogeochemical cycles across spatial and temporal scales [1–3]. Among them, coccolithophores are calcifying haptophyte algae that combine photosynthetic carbon fixation with the production of calcium carbonate plates (coccoliths), making them important contributors to marine carbon cycling [4, 5]. Their life cycle alternates between haploid and diploid phases, which differ in coccolith morphology, calcification traits, motility, cell size, physiology, and environmental distribution, thereby expanding the ecological breadth of the group across contrasting environments [6]. However, although coccolithophore biology has been studied extensively [7], the role of their associated microbiome in shaping host performance and ecology is only beginning to be resolved.

Like other phytoplankton, coccolithophores interact with diverse bacteria in the phycosphere, a microscale environment enriched in algal exudates, where bacteria both influence and respond to host metabolism [8, 9]. In coccolithophores, coccoliths may further shape interactions by providing surfaces for microbial attachment and by altering the microenvironment around the host cell. These effects could influence microbial colonization, nutrient exchange, and symbiotic interactions [10, 11] and have also been proposed to alter local pH dynamics [12, 13] and potentially link organic matter turnover to calcification [14].

More broadly, phycosphere interactions depend on both the quantity and quality of algal carbon release [8, 15, 16]. Experimental studies with synthetic phycospheres showed that bacterial communities can be shaped predictably by the metabolite composition of the surrounding environment [17], and in nature, phytoplankton blooms can drive substrate-controlled succession in marine bacterioplankton, with shifts in community composition linked to changing availability of bloom-derived organic matter [18].

B vitamins are low-abundance but high-impact growth factors that can structure microbial communities and mediate host–microbe interactions [19]. Over evolutionary timescales, such interactions can generate metabolic interdependencies, including partitioned B-vitamin biosynthesis in which algal hosts lacking complete de novo pathways rely on associated bacteria for vitamin supply or recycling [20–22]. Such auxotrophies are widespread among marine algae, including numerous haptophytes [23]. Yet the identity of partners, the routes of exchange, and the stability of these interdependencies remain unclear.

A key limitation is the uneven genomic representation of calcifying haptophytes, coccolithophores, which constrains comparisons of host traits and phycosphere assembly across taxa. Generating coccolithophore genomes can be challenging because some species have intron-rich, repeat-rich genomes [24], and sequencing from non-axenic cultures further complicates assembly by mixing host and microbial DNA [25]. Long-read sequencing helps mitigate these challenges by increasing assembly contiguity [26], while in mixed datasets it can also improve the recovery of associated microbial genomes [27]. Although high-quality assemblies exist for several haptophytes [28], genomic resources remain concentrated on *Gephyrocapsa (Emiliania) huxleyi* [29], which captures only part of coccolithophore trait diversity because it is lightly calcified, calcifies only in the diploid stage [30, 31], and belongs to a relatively young, opportunistic lineage [32, 33].

To complement insights from *G. huxleyi*, we focused on *Calcidiscus leptoporus*, a cosmopolitan coccolithophore that calcifies in both life-cycle phases and forms larger, more heavily calcified coccoliths. Global estimates suggest that *G. huxleyi* accounts for only ~7.2% of coccolithophore inorganic carbon standing stock, with more heavily calcified taxa, including *C. leptoporus*, accounting for most of the remainder [34, 35]. Moreover, the larger cell size and coccosphere of *C. leptoporus* could also expand the physical and chemical interface for phycosphere interactions. Together with its suitability for long-term cultivation and perturbation, these traits make *C. leptoporus* a complementary reference for testing vitamin-mediated microbial interactions.

Here we (i) expand coccolithophore genomic resources by assembling the nuclear genome of the haploid strain of *C. leptoporus*, (ii) reconstruct vitamin biosynthetic potential within the holobiont, here defined as the coccolithophore strain and its bacterial community, and (iii) assess how vitamins affect the holobiont in a long-term supplementation experiment. We reasoned that if key metabolic functions are partitioned across the holobiont, altering vitamin availability should affect both host physiology and microbial composition. Although haploid and diploid cells are expected to share most genomic biosynthetic potential, physiological and regulatory differences between phases [36] could shift vitamin demand, uptake, or bacterial provisioning dynamics in natural populations. The resulting data connect predicted metabolic complementarity with holobiont-level responses to vitamin perturbation of a haploid phase *C. leptoporus*.

## Materials and methods

### Strain selection

For genome sequencing of *C. leptoporus*, we selected a haploid strain (RCC1151, Roscoff Culture Collection) to facilitate assembly by minimizing allelic heterozygosity, which can inflate contiguity and size estimates in diploid genomes [37]. We retained the non-axenic community to examine links among host vitamin auxotrophy, bacterial biosynthetic potential, and phycosphere dynamics. Although culture phycospheres do not capture full in situ diversity, phytoplankton strains can establish reproducible host-associated consortia that stabilize and persist, suggesting host-driven selection rather than transient culture effects [38]. We therefore treat the RCC1151 as a tractable holobiont model under controlled perturbations, without claiming to represent the full natural *C. leptoporus* microbiome.

Calcification of RCC1151 was confirmed by scanning electron microscopy (ZEISS EVO-100; Zeiss, Jena, Germany). Culture aliquots were fixed with glutaraldehyde (5% final; Sigma-Aldrich) overnight at 4°C, gently filtered onto 0.4 μm membrane filters (Isopore PC; Merck Millipore), rinsed with pH-8 Milli-Q water to remove salts, and dried overnight at 50°C. Filter pieces were mounted on aluminum stubs with carbon tape, gold-coated, and imaged.

### Cultivation, physiological monitoring, and biomass harvesting

Semi-continuous batch cultures were maintained at 18°C in a climate chamber (INKOLAB, Zagreb, Croatia) under a 16:8 h light–dark cycle with white LEDs (68 μmol photons m^−2^ s^−1^) in K + Si/2 medium [39] which is supplemented with vitamins B1, B7, and B12. Medium was prepared from aged seawater, filtered (0.22 μm), sterilized by microwave boiling, cooled, nutrient-amended, and re-filtered (0.22 μm) [40]. Cultures were maintained in 50 ml vented tissue-culture flasks, while for DNA extraction and experiments we used 200 ml flasks (TPP Techno Plastic Products AG, Trasadingen, Switzerland).

Growth was monitored by minimum chlorophyll fluorescence yield (F0; DUAL PAM-100, Heinz Walz GmbH, Effeltrich, Germany) and correlated with manual counts performed using a Sedgewick Rafter counting chamber (Paul Marienfeld GmbH & Co. KG, Lauda-Königshofen, Germany) and an inverted AE2000 Trinocular microscope (MoticEurope S.L.U., Cabrera de Mar, Barcelona, Spain) (Fig. S1). Subsequent growth monitoring during the experiments used F_0_ only. For DNA extraction, exponential cultures were harvested twice over one month by centrifugation (4000 rpm, 10 min), resuspended in 500 μl K + Si/2, and stored at −20°C.

### High-molecular weight DNA extraction and sequencing

High-molecular-weight DNA was extracted using a CTAB–phenol–chloroform protocol [41]. Long-read libraries were prepared with the ONT Ligation Sequencing Kit V14 (SQK-LSK114; Oxford Nanopore Technologies, Oxford, UK) with 2 μg short read eliminator (SRE)-treated DNA in 48 μl input, 1 h adapter ligation, and long fragment buffer washes. Library quality was checked on a TapStation (Agilent), and sequencing was performed at P3S (Pitié-Salpêtrière, Paris, France) on FLO-PRO114M flow cells loaded with 24 fmol DNA (~60% pore occupancy).

### Genome assembly, binning, and refinement

Raw fast5 reads were base-called with Guppy (“Super Accuracy”) and adapters trimmed with Porechop v0.2.3_seqan2.1.1. Because sequencing was performed on DNA extracted from a non-axenic culture, we used Flye v2.9 [42] in metagenome mode, using --nano-hq --meta (other parameters default) to assemble the mixed dataset prior to downstream binning and manual refinement.

Assembly graphs were inspected in Bandage [43]. Contigs were binned with Metabat2 v2.15 [44] and evaluated with CheckM v1.2.2 [45]. Bins meeting ≥90% completeness and ≤1% contamination were retained; contig reassignment and curation are described in Supplementary Text. Curated bins were polished with two rounds of Racon v1.5.0 followed by Medaka v1.11.1 (dna_r10.4.1_e8_sup.cfg). MAG statistics are provided in Table S6, and MAG FASTA sequences/annotations are deposited in NCBI under SAMN47336961–SAMN47336966.

### Genome annotation and functional prediction

Repeats in the *C. leptoporus* nuclear genome (MAG_1000) were masked with RepeatMasker [46]. Structural annotations were performed with BRAKER3 (v3.0.5) in ETP mode using RNA-seq and protein evidence [47–56]. Initially, relatively few genes were annotated, due to the default parameters of TSEBRA; several sets of parameters were further tested, and the final gene set, comprising 32 457 predicted genes represented by 34 225 transcripts, was obtained with the following changes: intron_support = 0.2, sta_sto_support = 0.5. Turning off the option “--filter_single_exon_genes” yielded about twice more annotations without improving the BUSCO score, so this filtering was retained.

The RNA-seq dataset (SRR1300371), corresponding to diploid *C. leptoporus* strain RCC1130 [57], was downloaded from the NCBI SRA using SRA-tools v2.11.0 and used only to support structural gene prediction. Protein evidence comprised Eukaryota_odb11 orthologs (https://bioinf.uni-greifswald.de/bioinf/partitioned_odb11/Eukaryota.fa.gz) [58] and RefSeq haptophyte proteins (https://www.ncbi.nlm.nih.gov/protein/?term=txid2830[organism:exp)[59]. RNA-seq reads were aligned with STAR v2.7.11a [60], (--alignIntronMax 10 000 --alignMatesGapMax 10 000), assessed using STAR metrics (Supplementary Table S3), and visualized in JBrowse2 [61].

This Whole Genome Shotgun project has been deposited at DDBJ/ENA/GenBank under the BioProject accession PRJNA1235139. The version described in this paper is version SAMN47336967.

A metabolic reconstruction of *C. leptoporus* was generated by parsing the genome annotations through the Kyoto Encyclopedia of Genes and Genomes (KEGG) and International Union of Biochemistry and Molecular Biology (IUBMB) databases [62] into a systems biology model, available at https://www.patmedb.org/protists/calcidiscus (see Supplementary note for details). Trophic mode was inferred from genome-based functional annotations and compared with the MMETSP transcriptome of strain RCC1130 [57] using the gene-based predictive framework of Burns *et al.* [63].

### Bacterial MAGs

Taxonomic assignment of bacterial MAGs was performed with GTDB-Tk v2.3.2 [64] in KBase [65], which places genomes within the GTDB reference framework using bacterial marker genes. For phylogenetic analysis, a species tree was reconstructed in KBase using SpeciesTreeBuilder v0.1.4 from a GenomeSet containing the bacterial MAGs and the closest GTDB representative genomes identified by GTDB-Tk. SpeciesTreeBuilder identifies 49 core universal COG marker genes, inserts query genomes into curated and GBLOCKS-trimmed multiple-sequence alignments, concatenates these alignments, and infers an approximately maximum-likelihood tree using FastTree2 v2.1.10 (-fastest). The resulting phylogeny was visualized in FigTree v1.4.4 and refined in CorelDraw. MAGs were annotated with kb_DRAM [66]. KEGG orthology (KO) identifiers were grouped into pathway categories based on KEGG annotations (accessed 2024–2025) [67]. KO profiles were analyzed/visualized in R (tidyverse), with additional annotation support from GhostKOALA [68] and targeted homology searches.

### Vitamin biosynthesis gene phylogenies

We more closely examined the evolutionary origin of biotin (bioF, bioK, bioC, bioD, bioA) biosynthesis in *C. leptoporus* and its associated microbial community. Reference sequences were retrieved from UniProtKB, and homologs were identified by BLASTp searches (e-value <1e^−5^) against NCBI databases, with representative hits from early rounds used as additional queries in subsequent searches to broaden phylogenetic sampling. Individual genes were aligned with MAFFT v7.308 (AUTO) in Geneious v9.1.8 (Biomatters Ltd., Auckland, New Zealand), concatenated, and partitioned by gene. Maximum-likelihood analyses were performed on the concatenated protein sequence data, with each gene treated as a separate partition. The best-fit evolutionary models for each partition were selected using ModelFinder in IQ-TREE v2.0 [69, 70], and branch support was assessed with 1000 ultrafast bootstrap replicates (UFBoot; robust support ≥95%). Analyses were run with the --runs 10 option to ensure convergence.

For pantothenate, panB/panC homologs were identified by BLASTp against UniProt (accessed February 2024) [71], JGI PhycoCosm [72], and decontaminated MMETSP and OneKP transcriptomes [57, 73], using a relaxed e-value threshold of 1 × 10^−5^ for initial candidate recovery. Retrieved sequences were reciprocally searched against the *C. leptoporus* proteome (-max_target_seqs 1), retaining best panB/panC hits, and screened for PanBC-associated Pfam domains using HMMER/hmmsearch. Sequences were aligned with MAFFT, trimmed with trimAl (-gt 0.5) [74], curated in Geneious v10.0.9 [75], based on alignment quality and phylogenetic placement, and analyzed with IQ-TREE v2 (LG + R10; 1000 bootstraps) [76], as detailed in the Supplementary Text Methodology. Fusion proteins were defined as single, non-truncated sequences containing both domains; fusion counts and taxonomic summaries were generated in R. Complete sequences, annotations, inclusion/exclusion rationale, alignments, and tree topologies are provided in Supplementary Dataset S1.

### Design of the Long-term Vitamin Experiment

To investigate the metabolic interdependencies between *C. leptoporus* and its associated bacterial community, we manipulated B-vitamin availability in K + Si/2 medium during a four-month experiment under five treatments: (K) K-medium vitamin mix (B1, B7, B12 at 0.5 mg l^−1^, 2.5 μg l^−1^, and 2.5 μg l^−1^, respectively), (ø) no vitamins, (B1) B1 only at the K-medium concentration, (K + B6) K-medium vitamin mix plus B6 at 0.6 mg l^−1^, and (All) all eight B vitamins (K-medium vitamin mix plus B2 at 0.4 mg l^−1^; B3, B5, B9 at 50 μg l^−1^ each; and B6 at 0.6 mg l^−1^). These treatments were designed as targeted long-term perturbations rather than a full factorial B-vitamin screen, retaining the standard K-medium vitamin mix because B1, B7, and B12 matched predicted host biosynthetic gaps, while B1-only tested thiamine-specific effects and K + B6 and All tested broader supplementation effects.

Treatments were triplicated in 200 ml flasks inoculated with 10 ml exponentially growing non-axenic cultures. Cultures were maintained at 18°C (16:8 h light:dark; 68 μmol photons m^−2^ s^−1^) and transferred into fresh media every 3–4 weeks, depending on logistics. The design comprised an initial growth-cycle immediately after transfer into the treatment media (“Start”), followed by repeated sub-culturing for 4 months and a final growth-cycle (“End”). Here, “growth-cycle” refers to one batch-culture interval from inoculation into fresh medium to stationary phase. 16S rRNA samples were collected at the “Start” stationary phase and at the exponential and stationary phases of the “End” growth-cycle (Fig. 4a).

### Coccolithophore physiology

Coccolithophore physiology was assessed by measuring chlorophyll fluorescence parameters—minimal chlorophyll fluorescence yield (F₀), maximal quantum yield of photosystem II (F_v_/F_m_), and relative electron transfer rate (ETR)—using a DUAL PAM-100 fluorometer. To evaluate immediate and long-term responses, 2 ml samples were collected every second day during the “Start” and “End” cycles, respectively.

### Sample collection and processing for 16S rRNA data

For bacterial community profiling, samples were collected at “Start” stationary phase and at “End” exponential and “End” stationary phases. For each sample, 150 ml of culture (mean 2.1 × 10^5^ cells ml^−1^) was centrifuged (4000 rpm, 10 min); pellets were resuspended in 500 μl K + Si/2 medium and stored at −20°C until extraction. Genomic DNA was extracted with the DNeasy PowerLyzer Microbial Kit (Qiagen, Hilden, Germany) and quantified on a BioSpec-nano spectrophotometer (Shimadzu Biotech, Kyoto, Japan).

Amplicon sequencing (V3–V4; primers 341F (5′-CCTACGGGNGGCWGCAG-3′) and 805R(5′-GACTACHVGGGTATCTAATCC-3′)) was performed by Macrogen (Seoul, South Korea) using the Illumina 16S Metagenomic Sequencing Library Preparation workflow (Part #15044223 Rev. B) and sequenced on an Illumina MiSeq (2 × 300 bp). Demultiplexed FASTQ files (adapters/barcodes removed) were returned for downstream analysis.

### Bioinformatic processing of 16S rRNA data

Reads were processed in QIIME2 v2024.5 [77]. Primers were removed with Cutadapt v2024.5.0 [78] and ASVs were inferred with DADA2 [79] via the q2-dada2 plugin. Because reverse-read quality was low (40%–50% read loss), downstream analyses used forward reads only, denoised with a truncation length of 241 bp. Taxonomy was assigned with q2-feature-classifier [80] using a naïve Bayes classifier trained on the SILVA v138.2 [81] reference sequences extracted with RESCRIPt v2024.5.1 [82] for the 341F–805R V3–V4 target region in forward orientation. This classifier was then applied to the retained forward-only ASVs.

ASVs were aligned with MAFFT and a phylogeny built with FastTree2 [83]. Chloroplast, eukaryotic, and unassigned-domain reads were removed. Downstream analyses/visualization were performed in R v4.3.2 (qiime2R v0.99.6; ggplot2 v3.5.1); 16S rRNA ASV sequences and the code used to analyze them are available on GitHub (https://github.com/bbvukovic/Calcidiscus_Genome_Phycosphere_Interactions_2025/tree/v1.0.0).

### Statistical analyses of bacterial community structure

Vitamin effects on bacterial community composition were evaluated from relative abundances in the 16S rRNA ASV-derived bacterial taxon abundance table. Canonical correspondence analysis (CCA) [84] was performed in CANOCO v5.12 on the full taxon table. Relative abundances were square-root transformed before analysis. Growth-cycle phase (“Start” stationary, “End” exponential, “End” stationary) and vitamin treatments were included as categorical explanatory variables. Individual vitamin concentrations were not used as independent explanatory variables because they were collinear by experimental design.

SIMPER [85] was run in PRIMER v6 as a post hoc descriptive analysis to identify taxa contributing to within-treatment similarity within each growth-cycle phase, using Bray–Curtis similarity matrices calculated from square-root-transformed relative abundances in the 16S rRNA ASV-derived bacterial taxon abundance table. Taxa contributing ≥3% to within-treatment similarity in at least one phase × treatment group were retained for visualization and interpretation. Because only one no-vitamin replicate yielded sufficient sequence data at the “End” exponential phase, this sample was retained for CCA and descriptive visualization of community composition, but within-treatment SIMPER similarity was not calculated for this group.

## Results

### Genome is intron-rich and repeat-dense, with pervasive interstitial telomeric tracts

**Figure 1:**
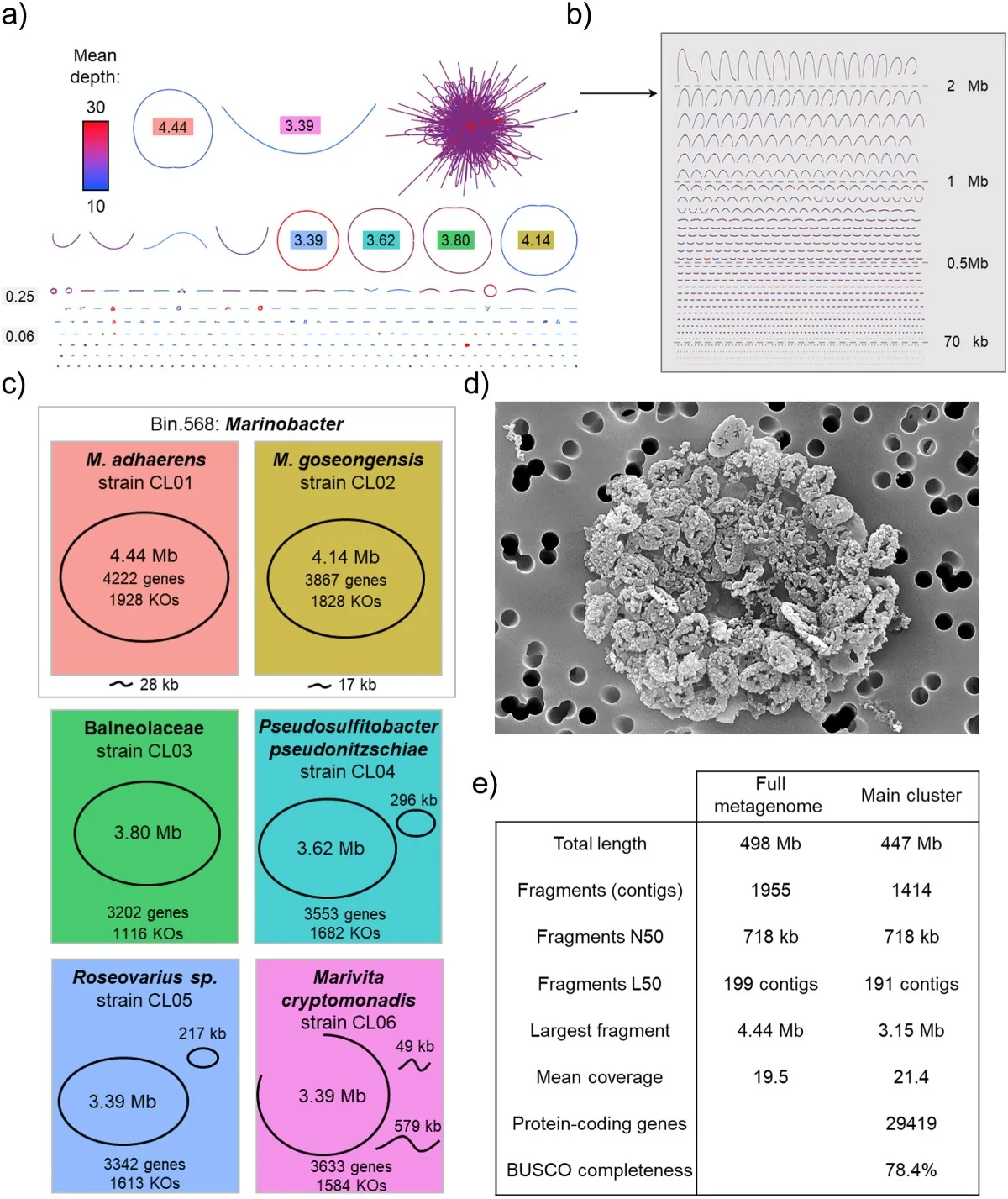
Metagenome of *C. leptoporus* RCC-1151 and its phycosphere. The non-axenic culture was grown in K/2-derived medium, which is notably supplemented with vitamins B1, B7, and B12. (a) Assembly graph of the full metagenome. Edges, representing assembled DNA sequences, are sorted by length, with lengths indicated in megabase pairs (Mb) in colored and grey boxes. Color scale depicts mean sequencing depth. Closed fragments indicate circular chromosomes. The upper right graph represents the *C. leptoporus* nuclear genome, strongly entangled due to abundant repetitive DNA sequences. (b) Edges of the nuclear genome, omitting nodes i.e. actual and artefactual fragment contiguity (connexions). (c) Map of the bacterial metagenomes. Background colors represent bins of contigs that display similar sequence patterns. MAGs contain the depicted contigs, taxonomic assignment, contig length, count of protein-coding genes, and count of distinct KO annotations. (d) Scanning electron microscope picture of collapsed coccosphere of *C. leptoporus* haploid strain RCC-1151. Scale bar: 2 μm. (e) Assembly statistics. The full metagenome is the final, unbinned assembly including *C. leptoporus* (nuclear, chloroplast, and mitochondrial) and bacterial genomes. The main cluster represents the nuclear genome of *C. leptoporus*, which forms a connected graph due to abundant repetitive genetic elements. N50 indicates that at least 50% of the total nucleotides belong to contigs larger than the indicated length. L50 indicates that at least 50% of the total nucleotides belong to the indicated number of contigs. BUSCO completeness assessed gene-space completeness as the proportion of universal single-copy orthologs detected and is expressed in percentages. Created in BioRender.

We sequenced the *C. leptoporus* haploid strain RCC1151 using long-read technology, retaining 1.7 million reads (10.3 Gb, Table S1). De novo assembly produced a 447 Mb metagenome (1955 contigs, Fig. 1), in which the assembly graph contained a large, highly connected cluster representing the host nuclear genome (Fig. 1a and b). Five >3 Mb contigs formed circular structures consistent with complete bacterial chromosomes (Fig. 1c). We also recovered circular chloroplast (110 kb) and mitochondrial (42.9 kb) genomes (Figs S2, S3). Scanning electron microscopy confirmed the characteristic haploid coccolith morphology of RCC1151 (Fig. 1d).

Host and bacterial contigs were separated by composition- and coverage-based binning followed by manual curation (Supplementary Text). The host nuclear genome (MAG_1000) corresponded to bin.372 and comprised 398 Mb (810 contigs). This bin showed 97% overlap with the large host-associated cluster identified independently in the Flye assembly graph (Fig. S4b), with GC 59% and ~ 20× mean depth. Assembly contiguity metrics were L50 = 191 contigs (>718 kb) and L90 = 629 contigs (>205 kb) (Fig. S4a), and CheckM did not indicate bacterial contamination.

The nuclear assembly was intron-rich and repeat-associated. Canonical GT/AG splice motifs predominated (75%; Table S2), and repeats/low-complexity sequence accounted for ~11% of the assembly (Table S2), including 130 short contigs (0.5–40 kb; ~1 Mb total) consistent with repetitive elements. The canonical telomeric repeat (TTAGGG) formed kilobase-scale arrays at contig termini, but also occurred frequently as interstitial telomeric sequences (ITSs) throughout the genome (Fig. S5), limiting the use of telomeric motifs for scaffolding and likely contributing to assembly fragmentation.

RNA-seq reads mapped with 81% unique alignment (36 million reads; Table S3) supporting broad capture of expressed genes. Gene prediction yielded 29 419 protein-coding loci (mean length 4093 ± 3817 bp), with 88% containing at least one intron (Table S4, Fig. S6). BUSCO analysis recovered 77.3% complete single-copy orthologs (11.0% duplicated; 17.3% fragmented), consistent with an intron-rich genome in which some loci remain split or redundant.

Assembly fragmentation can in principle truncate or split gene models and thereby affect inference of gene absence. However, in RCC1151, fragmentation appears to arise primarily from repeat-rich regions, whereas coding regions are relatively compact; accordingly, downstream pathway inferences were based on manually curated focal genes and interpreted conservatively. Together, these features establish an intron-rich, repeat-dense *C. leptoporus* nuclear genome resource for downstream comparative and functional analyses.

### Comparative annotation indicates gene losses and phago-mixotrophy

Predicted proteins were annotated with KOs and compared to MMETSP *C. leptoporus* transcriptomes [57]. KO overlap between the genome and MMETSP datasets was 43% (Fig. S7), consistent with differences in expression conditions and/or annotation pipelines. Comparison with eight haptophyte proteomes in PhycoCosm identified 707 KOs shared across all species and 65 KOs unique to *C. leptoporus* (Fig. S7).

To assess genome functionalities, the predicted proteome was further assembled into a metabolic reconstruction and compared with the published *G. huxleyi* metabolic context. In *C. leptoporus*, we identified pathway modules involved in central carbon and carbohydrate metabolism, including pyruvate, glyoxylate, and dicarboxylate, propanoate, inositol phosphate, and N-glycan metabolism. Notably, enzymes associated with glycogen/starch synthesis and degradation, including maltase-glucoamylase and cellulose 1,4-beta-cellobiosidase, suggest a more elaborate predicted starch/glycan metabolism in *C. leptoporus* than in *G. huxleyi*.

Trophic potential was inferred from the RCC1151 genome and compared to the MMETSP transcriptome of strain RCC1130 [57, 63]. Both strains were identified as phago-mixotrophs (phagocytosis + photosynthesis + prototrophy) (Fig. S8).

### Six recurrent bacterial lineages define the phycosphere

**Figure 2:**
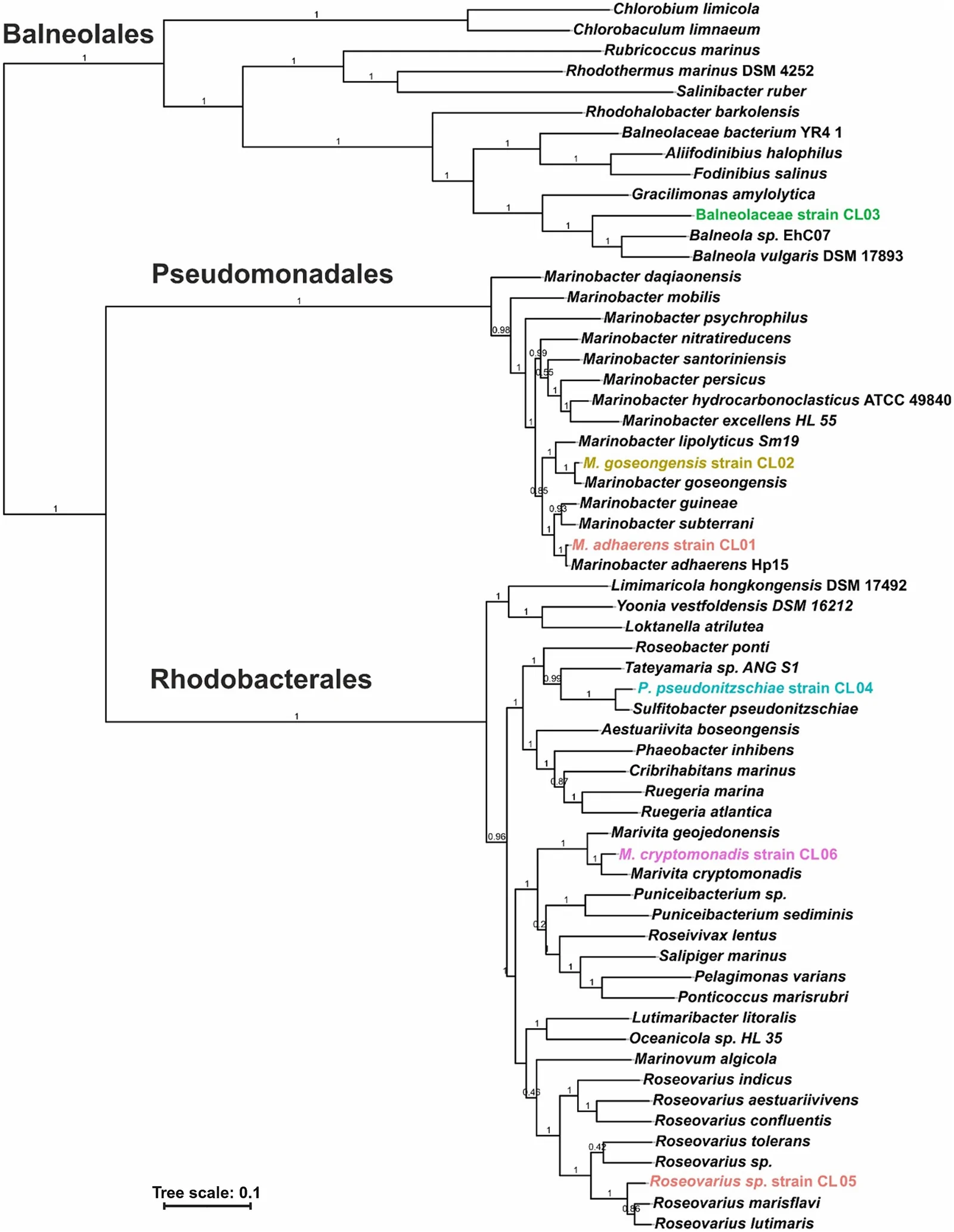
Phylogenetic assignment of the bacterial MAGs. MAGs obtained in this study (colored text) were aligned to the closest representative genomes (black text), and a phylogenetic tree was constructed using Kbase. Taxonomic orders and bootstrap support values are shown above the corresponding branches.

To identify microbial contributors to holobiont metabolism, we reconstructed bacterial metagenome-assembled genomes (MAGs) from the non-axenic *C. leptoporus* culture. Binning and manual curation yielded six medium- to high-quality MAGs representing recurrent phycosphere lineages (Fig. 1c, Supplementary Text). Taxonomy was assigned using genome-wide phylogenies (Table 1; Fig. 2; Table S4) and supported by protein alignments and KO profiles (Data S2).

**Table 1:** Bacterial genomes. Bacterial genomes were assessed using Checkm (see details in Supplementary Table S6), BLASTn on NCBI database, and GTDBtk (v2.4.0)—a toolkit for assigning objective taxonomic classifications to bacterial and archaeal genomes. Abbreviations: L, length (Mb); Comp., complete; Cont., contamination; AL, alignment length (kb).

Strain name	NCBI	L (Mb)	GC %	Depth	Checkm			Best blast hit 16S database NCBI				GTDBtk
					Marker lineage	Comp.	Cont.	Species	Accession	Identity %	AL (kb)	
*Marinobacter adhaerens* strain CL01	x	4.44	57.2	13	Class Gammaproteobacteria	99.1	0.1	*Marinobacter adhaerens*	NR_074765.1	100	1.4	*Marinobacter adhaerens*
*Marinobacter goseongensis* strain CL02	x	4.13	59.2	12	Class Gammaproteobacteria	100.0	0.4	*Marinobacter goseongensis*	NR_044340.1	99.65	1.4	*Marinobacter sp017643965*
Balneolaceae strain CL03	x	3.80	39.1	21	Kingdom Bacteria	99.7	0.0	*Balneola vulgaris*	NR_042991.1	94.90	1.5	Family: Balneolaceae; Genus: JAUICM01
*Pseudosulfitobacter pseudonitzschiae* strain CL04	x	3.61 + 0.30	59.9	22	Family Rhodobacteraceae	99.4	0.3	*Pseudosulfitobacter pseudonitzschiae*	NR_178596.1	99.20	1.4	*Pseudosulfitobacter pseudonitzschiae_A*
*Roseovarius* sp. strain CL05	x	3.39 + 0.22	60.8	28	Family Rhodobacteraceae	98.2	0.0	*Roseovarius lutimaris*	NR_109378.1	99.64	1.4	*Roseovarius sp913054395*
*Marivita cryptomonadis* strain CL06	x	3.39* + 0.63	57.7	8	Family Rhodobacteraceae	93.6	0.4	*Marivita litorea*	NR_044513.1	99.85	1.3	*Marivita sp947503115*

Two MAGs belonged to *Marinobacter* (*Marinobacter adhaerens* CL01 and *Marinobacter goseongensis* CL02, respectively). MAG_03 represented a novel Balneolaceae lineage (strain CL03). The remaining MAGs were Rhodobacterales: *Pseudosulfitobacter pseudonitzschiae* CL04, *Roseovarius* sp. CL05, and *Marivita cryptomonadis* CL06; CL06 was non-circular and slightly less complete (93%). Several additional bins were low completeness and excluded from downstream analyses (Table S4; Supplementary Text). No other eukaryotic primary producer was detected in the metagenome.

### Vitamin biosynthesis is partitioned; no single bacterium supplies all

We next examined manually curated B-vitamin biosynthetic potential across the holobiont to test for metabolic complementarity (Fig. 3; Figs S10–S19).

**Figure 3:**
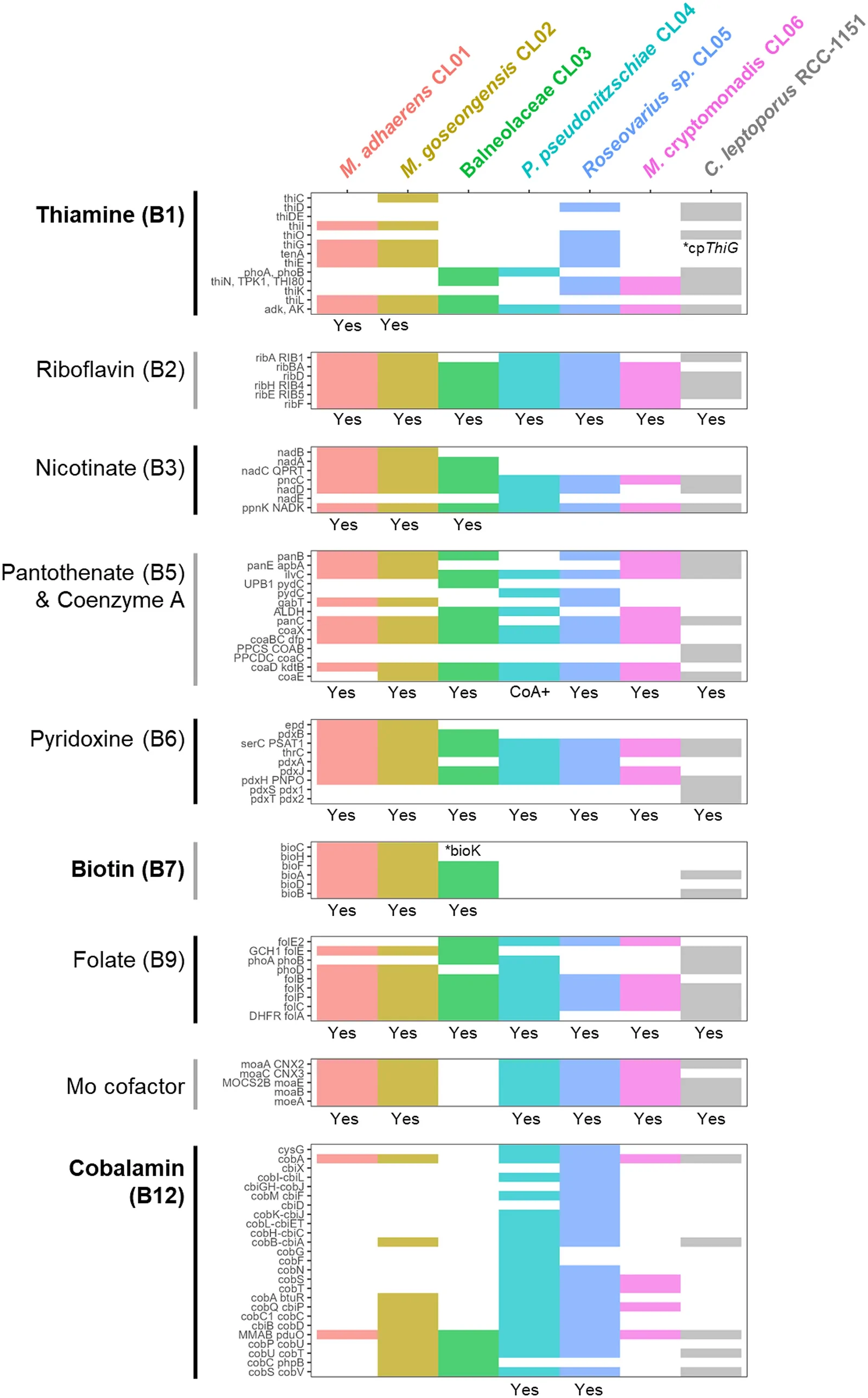
Biosynthetic pathways of B vitamins in the phycosphere. Bacterial and *C. leptoporus* genes derive from the KO annotations and are grouped in the context of biosynthetic pathways. Colored and blank tiles represent present and absent enzymes, respectively. To improve clarity, a few genes marginally related to the pathways are not shown. Yes stands for the complete pathway. Symbol “*” indicates (i) chloroplast-encoded *thiG* in thiamine pathway and (ii) the putative bioK function. In bold are vitamins of the K media vitamin mix.

- **B1 (thiamine).** Complete de novo thiamine biosynthesis was encoded by both *Marinobacter* (*M. adhaerens* CL01 and *M. goseongensis* CL02), whereas other MAGs encoded only partial salvage modules. The *C. leptoporus* genome lacked *thiC*, consistent with dependence on salvage/recycling rather than de novo synthesis (Fig. 3, Fig. S10).
- **B2 (riboflavin).** All members of the holobiont encoded a complete riboflavin pathway, with the sole gap being a hydrolysis step for which no prokaryotic enzyme is known (Fig. 3, Fig. S11).
- **B3 (nicotinate).**  *Marinobacter* strains CL01 and CL02 encoded the full *nadABC* operon for de novo nicotinate biosynthesis, whereas other MAGs and the host lacked a complete de novo set, indicating reliance on salvage/uptake (Fig. 3, Fig. S12). The NaMN transporter pnuC occurred in CL03 and CL04, and a niaP-like transporter was detected in the host (anno1.g6394.t1).
- **B5 (pantothenate) and CoA.** All MAGs except *P. pseudonitzschiae* encoded pantothenate biosynthesis (*panB* and *panC*), while all six MAGs encoded the downstream coenzyme A (CoA) pathway (Fig. S13). In *C. leptoporus*, pantothenate biosynthesis was completed by a single fused *panB-panC* gene supported by intron structure and genomic context; alternative KO assignments supported a complete CoA pathway (panK, COASY). A comparative survey across 5728 taxa identified 1678 species with *panB* and *panC* homologs but only 65 with validated *panB/panC* fusions (Fig. S14), confined to marine eukaryotes and present across major prymnesiophyte orders but absent from Pavlovophyceae.
- **B6 (pyridoxal/pyridoxine).** All bacterial MAGs encoded the DXP-dependent pathway, with apparent gaps resolved by targeted searches. The host encoded the DXP-independent *pdxS–pdxT* pathway, supporting de novo B6 synthesis (Fig. 3, Fig. S15).
- **B7 (biotin).**  *M. adhaerens* CL01 and *M. goseongensis* CL02 encoded the complete biotin biosynthesis pathway (Fig. 3, Fig. S16), whereas Rhodobacterales MAGs (CL04–CL06), as well as the host, lacked biosynthetic genes but encoded uptake capacity (e.g. *bioY*). In Balneolaceae CL03 a *bioF–bioK–bioC–bioD–bioA* operon consistent with de novo biosynthesis was supported by GhostKOALA/manual curation and cyanobacteria-like gene order (Fig. 3, Fig. S17).
- **B9 (folate) and MoCo.** Although MoCo is not a vitamin, we considered it alongside B9 because both pathways derive their pterin moieties from GTP, with folate supporting one-carbon metabolism and MoCo enabling molybdenum-dependent redox reactions [86–88]. Folate biosynthesis was present in all MAGs and the host (Fig. 3, Fig. S18), missing steps were resolved by alternative enzymes (e.g. *phoX* in CL06) and targeted host gene identification (*FolB*: anno1.g31798.t1). The molybdenum cofactor pathway was absent from the host and CL03 but present in the remaining MAGs.
- **B12 (cobalamin).** Only *P. pseudonitzschiae* CL04 and *Roseovarius* sp. CL05 encoded cobalamin biosynthesis, consistent with aerobic and anaerobic routes, respectively (Fig. 3, Fig. S19). All MAGs encoded *pduO* for cobalamin activation; only *Marinobacter* encoded the cobalamin-independent methionine synthase metE. The host lacked de novo cobalamin genes but encoded *pduO* and trafficking factors (*bacA*, LMBRD1), consistent with cobalamin auxotrophy.

Together, these data indicate that *C. leptoporus* can synthesize B2, B5, B6, and B9, but is salvage-dependent for B1 and B3 and auxotrophic for B7 and B12. Correspondingly, phycosphere bacteria provide complementary biosynthetic capacity, and no single MAG encodes the full set of host-missing vitamins, consistent with partitioned vitamin provisioning within the holobiont.

### B-vitamin availability shapes host physiology; ecological succession structures the microbiome

**Figure 4:**
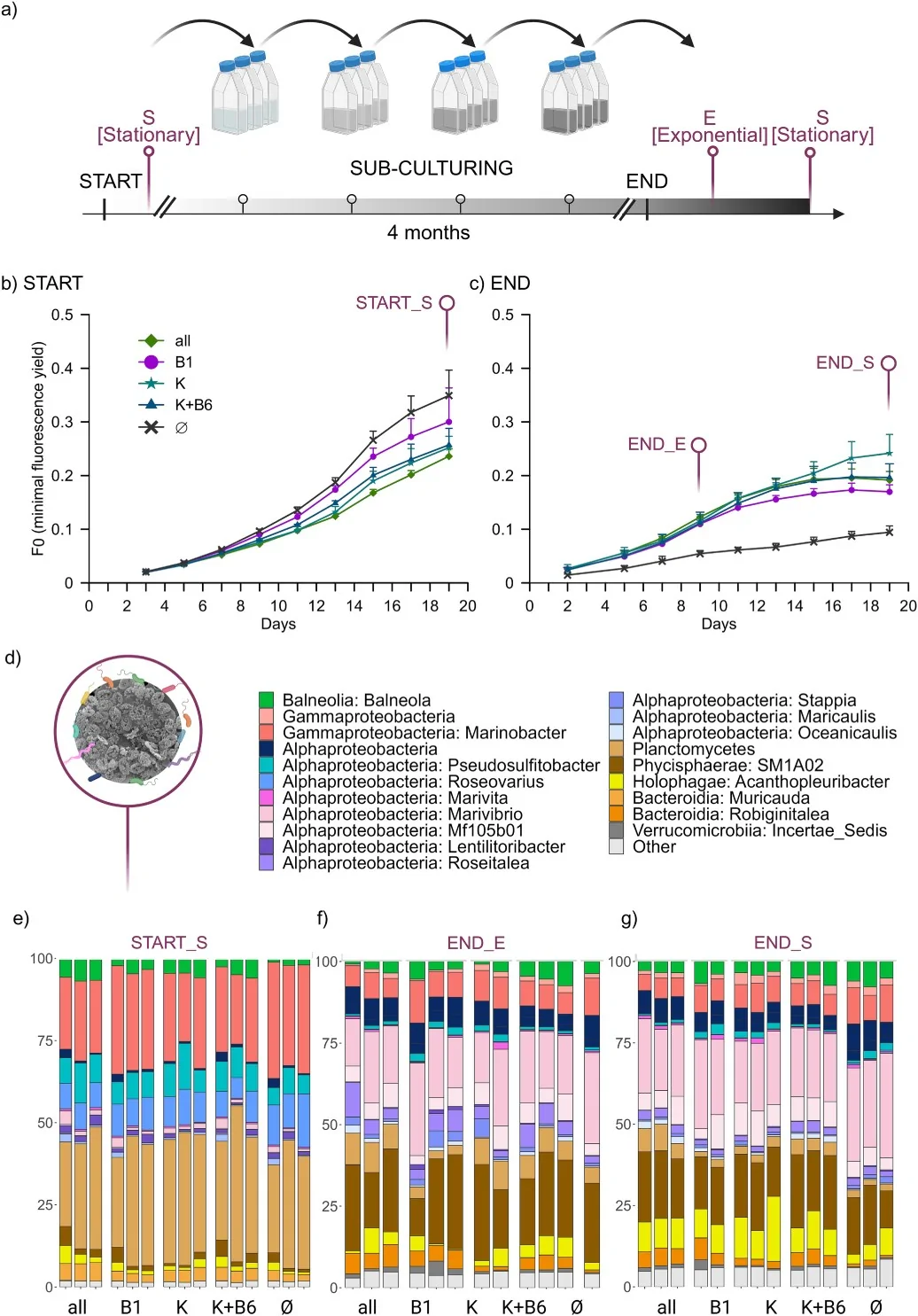
Experimental design and responses of the non-axenic *C. leptoporus* holobiont to long-term vitamin manipulation. (a) RCC1151 pre-cultures grown in K + Si/2 medium were transferred at stationary phase into five vitamin treatments and serially sub-cultured every 3–4 weeks for 4 months. Physiological responses were monitored during the initial culture cycle immediately after transfer (“Start”) and again during the final culture cycle after 4 months (“End”), while bacterial community composition was sampled at three points: The “Start” stationary phase and the exponential and stationary phases of the “End” growth-cycle. (b) Growth during the initial cycle after transfer (“Start”), shown as minimal chlorophyll fluorescence yield (F₀; mean ± SD, *n* = 3). (c) Growth during the “End” growth-cycle after 4 months of treatment (“End”). (d) Schematic representation indicating the sampling points and depicting a coccolithophore and its surrounding bacterial community, with the taxonomic groups shown in the community-composition plots. (e–g) Relative bacterial community composition based on 16S rRNA amplicon sequencing for replicate cultures sampled at the “START_S” stationary phase (e), the “END_E” exponential phase (f), and the “END_S” stationary phase (g). Treatments were K, K-medium vitamin mix; ø, no vitamins; B1, thiamine only; K+B6, K-medium vitamin mix plus B6; All, eight B vitamins. Created in BioRender.

Based on the inferred partitioning of B-vitamin biosynthesis across the holobiont, we tested how external vitamin availability shapes both host performance and community dynamics by cultivating *C. leptoporus* RCC1151 for 4 months under five defined vitamin treatments (Fig. 4). Because our cultures were non-axenic, treatment effects reflect holobiont-level responses rather than direct bacterial responses alone.

**Host responses.** During the initial culture cycle (“Start”), growth (F₀) was similar across treatments (Fig. 4, Fig. S20). After 4 months of repeated sub-culturing, the final growth-cycle (“End”) showed reduced growth and lower photophysiological performance in cultures grown without vitamins.

At the final sampled time point, no-vitamin cultures reached 39% of the F₀ observed under the K-medium vitamin mix, whereas B1-only cultures reached 70%, indicating partial rescue by thiamine (Fig. 4c). Broader vitamin-replete treatments reached 79%–81% of the K-medium vitamin mix response. Because B7 and B12 were supplied together in the K treatment and were not tested individually, their separate contributions cannot be resolved. The All-vitamins treatment did not further improve growth relative to the K-medium vitamin mix, indicating that the response was not simply additive across vitamins and may also reflect indirect effects in the non-axenic community.

PSII maximum efficiency (F_v_/F_m_) varied little among treatments, while electron transport rates (ETR) were much lower without vitamins (Fig. S20), indicating downstream metabolic limitation rather than impaired light harvesting. Although we did not measure vitamin concentrations during serial transfers, the responses to B1 and vitamin-replete treatments are consistent with the predicted thiamine auxotrophy of the host.

**Figure 5:**
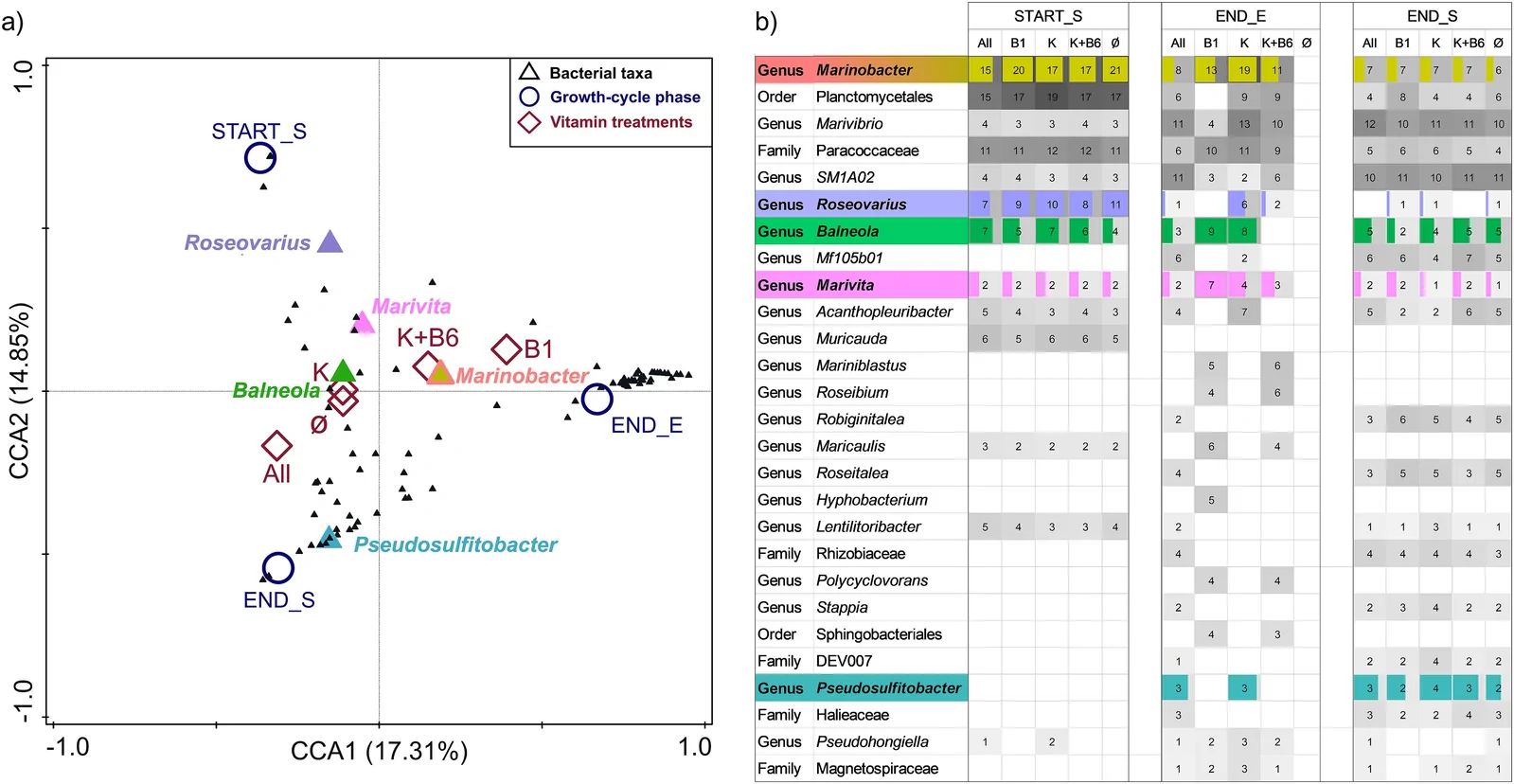
Bacterial community analysis across vitamin treatments and experimental phases. (a) CCA performed on the full 16S rRNA ASV-derived bacterial taxon table. Black triangles represent bacterial taxa included in the analysis, colored triangles indicate selected MAG-resolved focal genera, blue open circles represent growth-cycle phase classes, and burgundy open diamonds represent treatment classes. For readability, only selected MAG-resolved genera are labeled. (b) SIMPER contribution table showing bacterial taxa contributing ≥3% to within-treatment Bray–Curtis similarity in at least one phase × treatment group. Analyses were performed separately for the “Start” stationary phase (START_S), “End” exponential phase (END_E), and “End” stationary phase (END_E). Cell values indicate SIMPER contribution % values; blank cells indicate taxa not retained within the 90% cumulative contribution cut-off, except for the no-vitamin treatment at END exponential, where within-treatment SIMPER similarity was not calculated because only one replicate was available. Taxonomic labels correspond to the lowest reliable 16S rRNA assignment and therefore include multiple taxonomic ranks. Colored highlights indicate taxa corresponding to MAG-resolved bacterial lineages for which vitamin biosynthetic potential was reconstructed: *Marinobacter*, *Balneola*, *Marivita*, *Roseovarius*, and *Pseudosulfitobacter*.

**Microbiome dynamics.** Bacterial community composition was compared among the stationary phase of the initial “Start” growth-cycle and the exponential and stationary phases of the final “End” growth-cycle. CCA performed on the full 16S rRNA ASV-derived bacterial taxon abundance table showed that community composition was structured primarily by growth-cycle phase (Fig. 5a). The explanatory variables accounted for 38.5% of total variation, and CCA1 and CCA2 explained 17.3% and 14.9% of total variation, respectively, together representing 83.5% of the fitted variation. The constrained model was significant based on unrestricted permutation testing (all constrained axes: pseudo-*F* = 3.6, *P* = .0002; first constrained axis: pseudo-*F* = 7.1, *P* = .0002), and variance inflation factors were <2 for all explanatory variables. Term tests showed that growth-cycle phase was the dominant structuring factor, whereas treatment effects were weaker and treatment-specific, with only B1 showing a significant association with bacterial community composition.

Post hoc SIMPER analysis showed high within-treatment community similarity during both stationary phases and greater variability during the “End” exponential phase (Fig. 5b). Similarity ranged from 87.5% to 91.6% during the “Start” stationary phase and from 84.5% to 88.4% during the “End” stationary phase, but was lower in the “End” exponential phase for K and B1 treatments (44.6% and 42.1%), intermediate for K + B6 (55.1%), and high for the All treatment (84.6%); the no-vitamin treatment could not be evaluated at this phase because only one replicate was available. Recurrent contributors included *Marinobacter*, Planctomycetales, Paracoccaceae, *Balneola*, *Marivibrio*, SM1A02, Mf105b01, and other bacterial groups. In the “End” exponential phase, treatment-specific contributors included *Marivita* in the K and B1 treatments, *Pseudosulfitobacter* in the K treatment, *Polycyclovorans* in the B1 and K + B6 treatments, and *Roseitalea* in the All treatment. Thus, community turnover was driven primarily by growth-cycle phase, while vitamin treatment was associated with weaker, treatment-specific shifts within a broader recurrent bacterial assemblage.

## Discussion

This study establishes *C. leptoporus* as a complementary reference to the *G. huxleyi* for coccolithophore microbial ecology. By combining a long-read-based host genome, genome-resolved metagenomics, and a long-term vitamin perturbation experiment, we identify a partitioned B-vitamin network across the holobiont and link predicted metabolic potential to host and community responses under controlled cultivation.

### Genome architecture and metabolic potential in *C. leptoporus*

The haploid *C. leptoporus* assembly encodes 29 419 protein-coding genes, comparable to published *G. huxleyi* genomes [24]. Telomeric arrays with the canonical TTAGGG motif occurred at multiple contig termini, consistent with haptophyte telomere organization [89, 90]. However, TTAGGG also appeared as interstitial telomeric sequences, complicating chromosome inference and scaffolding, possibly reflecting lineage-specific rearrangement dynamics [91–93]. These features provide testable hypotheses for future chromosome-scale scaffolding and structural-variant analyses.

Comparative pathway reconstruction suggests that *C. leptoporus* differs from *G. huxleyi* not only in calcification traits [24, 29, 30], but also in predicted carbon-storage and carbohydrate-remobilization potential. Relative to the *G. huxleyi* metabolic context [94, 95], *C. leptoporus* contains enzymes associated with starch synthesis and degradation, suggesting a more elaborate starch/glycan metabolism that could support physiological buffering under fluctuating resource availability. However, direct measurements of storage compounds and carbon fluxes will be needed to test whether storage metabolism differs functionally between the two species.

Pantothenate biosynthesis revealed another genomic signature: a fused *panB–panC* gene. Comparative screening indicates that such fusions are rare and sporadically distributed across marine eukaryotes, consistent with repeated gains/losses and pathway remodeling in complex algal lineages [96–98]. Its absence from Pavlovophyceae shows that vitamin pathways can diverge within haptophytes, reinforcing the value of broader genomic sampling beyond current models.

Genome-based inference supports phago-mixotrophic potential in RCC1151, consistent with reported bacterivory in another *C. leptoporus* strain [99] and broader evidence for mixotrophy across haptophytes [100]. If expressed, phagotrophy could add prey–predator dynamics to phycosphere interactions, but its ecological role in *C. leptoporus* remains to be tested directly.

### Culture-associated bacteria and partitioned vitamin biosynthesis

Several bacterial lineages corresponding to the MAG-resolved taxa were repeatedly detected in the 16S rRNA dataset across growth-cycle phases and vitamin treatments, indicating that these taxa represented recurrent members of the culture-associated bacterial community. These lineages overlap with reported coccolithophore-associated communities [101, 102], but represent a within-culture core rather than a definitive in situ microbiome. Genome-wide DRAM/KEGG KO annotations indicate carbohydrate-related metabolic potential in these MAGs, while manual pathway reconstruction showed partitioned vitamin-biosynthesis capacity. Together, these predicted functions are consistent with adaptation to algal-derived substrates and potential cofactor provisioning, echoing other phytoplankton–bacteria associations [21, 101, 103, 104].

Several recovered lineages have ecological parallels in other phytoplankton systems: Roseobacters with *G. huxleyi* [9, 105, 106], *Marinobacter* and *Marivita* with algal-derived carbon pools [101, 103, 107], *Balneola* with *Phaeocystis globosa* colonies [108], and Planctomycetota with particle-associated, bloom-responsive communities [109]. Their recurrence across *C. leptoporus* cultures and wild associations [101, 102] is consistent with a host-filtered, culture-associated consortium with potential metabolic complementarity.

Metabolic reconstruction indicates a modular vitamin network: the host retained complete pathways for B2, B5/CoA, B6, and B9, but lacked de novo capacity for B1, B3, B7, and B12, which were distributed among bacterial partners. No single MAG encoded all host-missing vitamins, consistent with partitioned biosynthetic potential and possible functional complementarity within the phycosphere. Similar partitioning has been reported in other marine consortia and may stabilize associations when vitamins are scarce but essential [21, 104].

### B-vitamin availability and community turnover

The long-term perturbation experiment provided functional support for this genomic model. Cultures grown without vitamins showed reduced host growth and electron transport capacity, while supplementation patterns indicated that B-vitamin availability constrained host performance. B1 alone provided partial rescue, whereas K-containing and broader vitamin-replete treatments showed equal or stronger responses, suggesting that thiamine contributed to limitation but that additional vitamins may also have supported long-term host performance.

Stable PSII maximum efficiency but reduced electron transport suggests intact light harvesting with constrained downstream metabolism, consistent with thiamine-dependent carbon flux [110]. These results support the interpretation that genome-inferred vitamin gaps affect host performance under our experimental conditions, while avoiding the assumption that vitamins are the sole limiting factor in nature.

More broadly, vitamin biosynthetic potential does not necessarily translate into effective metabolite exchange. Natural marine microbial communities show mosaic patterns of B-vitamin synthesis and utilization [111], and auxotroph co-cultures in particle-associated communities can remain vitamin-limited despite complementary pathways [112]. This context supports a cautious interpretation of our results, in which vitamin-dependent responses reflect both metabolic dependencies and constraints on vitamin availability within the holobiont.

In contrast to the strong host response, bacterial community turnover was structured primarily by growth-cycle phase, with vitamin treatment acting as a weaker, specific modifier of community composition. This pattern is consistent with successional dynamics in phytoplankton-associated microbiomes, where shifts in host physiological state and algal-derived organic matter can select for distinct heterotrophic assemblages [18, 113, 114]. In our experiment, however, organic substrates and exudates were not measured directly, so the drivers of this phase-associated turnover remain unresolved. The higher within-treatment similarity observed during stationary phases, and greater variability during the final exponential phase, suggest that active host growth generated more variable phycosphere conditions. Recurrent taxa, including *Marinobacter*, Roseobacter-lineage taxa, Planctomycetales, Bacteroidota-related groups, and SM1A02, are consistent with algal-associated heterotrophy, including siderophore-mediated interactions, phytoplankton-derived metabolite use, and complex organic-matter processing [115–118].

Because these patterns are based on relative community composition, they are best interpreted as bacterial community restructuring across growth-cycle phases rather than as direct evidence for changes in vitamin supply. This interpretation is further limited by the non-axenic design, the lack of time-resolved vitamin measurements, and the absence of absolute bacterial abundances. Axenic, defined-media, or synthetic-community experiments will be needed to distinguish direct vitamin effects from host-mediated responses and to test exchange mechanisms more directly.

### Outlook

Together, our results position *C. leptoporus* as a genomic and experimental reference for a calcified coccolithophore holobiont, revealing a partitioned B-vitamin network in which vitamin availability can constrain long-term host performance, while phycosphere composition is structured primarily by succession with vitamin supply tuning late-stage niches. Next, this framework should be extended beyond a single culture by testing the consistency of host-associated consortia across strains and environments, resolving causality, exchange routes, and potential competition for shared vitamin pools with synthetic-community experiments, and coupling holobiont dynamics to direct measurements of calcification and carbonate chemistry to test whether microbiome-mediated effects extend to calcification-related traits. In the future, integrating these data into constraint-based models of host metabolism and microbial cofactor exchange could help quantify how vitamin treatments influence coccolithophore physiology and host-associated microbial interactions.

## Supplementary Material

Supplementary_material_ycag222

## Data Availability

The genomic datasets and raw 16S rRNA amplicon sequencing reads generated in this study are available through NCBI under BioProject PRJNA1235139 (https://www.ncbi.nlm.nih.gov/bioproject/PRJNA1235139). The *C. leptoporus* nuclear genome is associated with BioSample SAMN47336967, and the six bacterial metagenome-assembled genomes are associated with BioSamples SAMN47336961–SAMN47336966. The metabolic reconstruction of *C. leptoporus* is available through PatMeDB at https://www.patmedb.org/protists/calcidiscus. The analysis code used in this study is available as a versioned GitHub release at https://github.com/bbvukovic/Calcidiscus_Genome_Phycosphere_Interactions_2025/releases/tag/v1.0.0 and is permanently archived in Zenodo under the DOI https://doi.org/10.5281/zenodo.21620085.
