# Supplementary material for: The Calcidiscus leptoporus genome reveals vitamin-mediated holobiont interactions
Source: ISME Commun. 2026 Aug 23;6(1):ycag222. doi: 10.1093/ismeco/ycag222 (PMC13518057; doi:10.1093/ismeco/ycag222)
Supplement: Supplementary file 1 [file supplementary_material_ycag222.zip › Chaux_Supplementary_Materials_ISMEComm.pdf]

Supplementary Materials for

Reference genome of the calcifying phytoplankton *Calcidiscus* reveals vitamin B–mediated phycosphere interactions

Frédéric Chaux *et al.*

*Corresponding author. Email: jelena.godrijan@irb.hr

This file includes:

- Supplementary Text

1. Methodology
2. Results

- Supplementary Figures: S1 to S18

S1: Comparison of cell counts vs F0 (minimal fluorescence yield) of *Calcidiscus leptoporus* cultures.
S2: The chloroplast genome of *Calcidiscus leptoporus*.
S3: The mitochondrial genome of *Calcidiscus leptoporus*.
S4: Contigs distribution and initial metagenome binning.
S5: Identification of telomere motif and its distribution in the nuclear genome.
S6: Length distribution of genomic features.
S7: KEGG orthology (KO) distribution.
S8: Trophic mode predictions from *C. leptoporus* genome
S9: Original bins and re-assignment
S10: Major steps of the thiamine biosynthesis.
S11: Major steps of the riboflavin biosynthesis.
S12: Major steps of the niacin biosynthesis.
S13: Major steps of the pantothenate and coenzyme A biosynthesis.
S14: The gene fusion of panB and panC domains displays an intricate distribution among eukaryotes.
S15: Major steps of the pyridoxal biosynthesis.
S16: Major steps of the biotin biosynthesis.
S17: Identification of a novel member of the bioK family in *Balneolota* bacteria
S18: Major steps of the folate and molybdopterin cofactor biosynthesis.
S19: Major steps of the cobalamin biosynthesis.
S20: Growth and photosynthetic performance of *C. leptoporus* under various vitamin regimes.

S21: Metabolic reconstruction of *C. leptoporus*

- Supplementary Tables ST1 to ST6

ST1: Sequencing statistics
ST2: Repeat detection outputs using RepeatMasker on *Calcidiscus* genome (MAG_1000).
ST3: Statistics of mapping RNA-seq onto *Calcidiscus* genome (MAG_1000).
ST4: Gene detection in *Calcidiscus* genome (MAG_1000) statistics using braker3.
ST5: Putative horizontal gene transfers in *Calcidiscus* genome (MAG_1000).
ST6: CheckM statistics for bacterial MAGs.

- Supplementary Datasets – <u>full captions</u>

S1. Phylogenetic distribution and curation of panB and panC homologues.
S2 Best-scoring RAxML trees of inferred *Calcidiscus* HGTs. (SD1-SD23)
S3. Whole-proteome assessment of bacterial MAGs. (SD24-SD30).

Supplementary Text

1. Methodology

Organelle manual curation.

The chloroplast and mitochondrial assemblies of *Calcidiscus leptoporus* underwent targeted manual curation to correct gene models, as most automated annotation tools are tailored to green algal/plant plastids or animal mitochondria[1]. The plastid assembly initially produced was circular but the sequence was cut in the middle of petD, preventing correct protein annotation, so we rearranged the contig to start with rrns. Regarding protein-coding genes, based on homology with G. oceanica and some other haptophytes, the longest model (TTG as start codon) is considered for the gene atpI, which encodes an important ATP synthase subunit; the protein model is annotated 11 amino acids shorter in some other haptophytes (e.g. Phaeocystis globosa, Chrysochromulina parva), which seems truncated as compared to AtpI in diatoms and green algae (accession numbers: UPY84296.1, WKK50204.1, YP_009459221.1, YP_008145496.1, YP_010455844.1, YP_005089700.1). Regarding another crucial protein, PsbC (CP43), the start codon remains uncertain because several predictions have been proposed in haptophytes as well as in diatoms and the green lineage. Indeed, psbC and psbD (coding for subunit D2) form a co-transcribed operon in most species and the beginning of the open reading frame containing psbC overlaps with the end of psbD. However, in the green alga C. reinhardtii, psbC and psbD are not direct neighbours and CP43 starts with amino acids METLFN (Gallaher et al, Plant Journal 2017), and in tobacco, translation of psbC starts with GUG codon (valine) followed by ETLFN (Kuroda et al, Plant Cell Physiol 2007). We thus assume that the most likely psbC gene model for C. leptoporus (and haptophytes in general) starts with GUG and translates into METPFN.

Telomere and ITS analyses.

To identify telomeric repeats in *C. leptoporus*, we used Teloreader[2], a Python-based tool that scans for a specified motif and its circular permutations across sliding windows. We applied Teloreader to both the assembled genome and the full set of raw Nanopore reads, using the canonical *C. leptoporus* telomeric motif TTAGGG as input[3]. Default parameters were used for initial detection. For the genome assembly, we additionally conducted a more stringent search by setting the min_mean_telo parameter to 5.8 and min_mean_window to 6 to filter for high-confidence telomeric arrays. Figures were generated using the Seaborn and Matplotlib Python libraries. For visualisation of ITS, we performed multiple sequence alignment using the MUSCLE algorithm and visualised the results with Jalview.

Manual bin curation and reassignment

Detailed inspection of bins, identification of plasmids/artefactual binning, reassignment of contigs, treatment of complex bins, and handling of lower-quality MAGs.

Binning of the contigs (Metabat2) revealed the presence of short sequences, such as full, circular plasmids and partial plasmid, putatively associated to bacterial chromosomes. However, to avoid artefactual binning, we manually checked the largest bins and, in some cases, we re-attributed sequences to the final metagenome-assembled genomes (MAGs). With this curation step, we obtained checkm scores with both >98% completeness and <1% contamination for five out of the six well-resolved MAGs presented in this work. Moreover, extensive comparisons to reference and/or closely related species, i.e. whole-genome protein

alignment, assessment of the overlap of KO annotations, highlights the quality of these long read-based MAGs and the completeness of protein prediction.

bin.568

The largest bacterial bin (bin.568) contained four sequences: two large circular chromosomes (4.4 Mb and 4.1 Mb) and two small linear pieces (<30 kb) (Figure 1c). Assessing bins with Checkm indicated 100% completeness but also 100% contamination (Suppl. Figure S9; Suppl. Table ST6). Similarities between the two circular chromosomes covered only 46% of the nucleotide sequences with an average of 90% identity, and a large (2 Mb) inversion was detected, suggesting that these two chromosomes effectively belong to two distinct species. The two small fragments displayed 5-times lower sequencing depth than the circular chromosomes, partially aligned to *Alcanivorax* sp. and *Marinobacter salarius* (highest similarity >97% identity, e-values = 0), and one of them showed partial similarity to some regions on both circular chromosomes (85% identity) but not the other. For further investigation, the four sequences of bin.568 were hence treated separately (MAGs _01, _02, _101 and _102). Checkm scores suggest that MAG_01 and MAG_02 are near-complete (>99%) gammaproteobacterial genomes and contamination score (<0.5%) are due to a single marker gene duplication in both genomes.

bin.470 and bin.369

The second largest bacterial bin (bin.470) contained 41 AT-rich sequences: one large circular chromosome (3.8 Mb) and 40 smaller fragments with 5-fold less sequencing depth. In the assembly graph, these sequences were not connected together nor to the *Calcidiscus* genome cluster and Checkm contamination score was very high (84%). This bin was hence split into i) the chromosome (MAG_03) and ii) a new bin (MAG_103, total: 3.5 Mb), decreasing contamination to 0% while preserving 100% completeness for MAG_03 (Suppl. Figure S9; Suppl. Table ST9).
On the other hand, exploratory analyses using GTDBtk place MAG_103 within the **Pleioneaceae** (Gammaproteobacteria). Based on the distribution of contigs (Suppl. Figure S4), MAG_103 gathered at low GC and low depth together with only one other bin, bin.369. Grouping these two sets of sequences improved completeness (88%) but also increased contamination (26%), suggesting that it may contain several more or less phylogenetically distant species. For this reason and the overall low genome abundance, completion and contiguity, MAG_103 and bin.369 were not retained for the further investigations in this paper.

bin.564

The fourth largest bacterial chromosome (3.6 Mb, circular) was found in bin.564, together with another circular sequences of 296 kb with similar sequencing depth. Moreover, two regions of 6.1 kb and 1.3 kb displayed >99.8% identity between both sequences, suggesting that they belong to the same species. Hence, bin.564 was conserved as such and only renamed MAG_04. This MAG scored 99% completeness using *Rhodobacteraceae* marker sets and >1% contamination (Suppl. Figure S9; Suppl. Table ST9). A contig attributed to another bin (bin.493) displayed sequence similarity and a large KO overlap specifically with *P. pseudonitzschiae*. However, in preliminary analyses, grouping together MAG_04 and MAG_104 did not improve completeness as compared to MAG_04 alone and rather increased contamination and heterogeneity.

bin.469

The third largest bin (bin.469) contained two large circular sequences (3.4 Mb and 216 kb) and 26 smaller sequences with 10-fold lower depth, but Checkm score indicated high contamination (25%). Both large circular sequences showed some similarity to *Roseobacteraceae* members and where hence grouped into a separate new bin (MAG_05), yielding a 98% completeness score (*Rhodobacteraceae* markers) and very low contamination.
On the other hand, in the assembly graph, the smaller sequences from bin.469 were not connected together nor to the *Calcidiscus* genome cluster, and were grouped into a new bin (MAG_105, total: 1.8 Mb, GC *ca.* 62%, completion 27% of the bacterial markers). Exploratory analyses using GTDBtk and KO place it most specifically with the *Roseovarius* clade, suggesting that another *Roseovarius* species may be present in low abundance in the phycosphere.

bin.493

The last large bacterial bin (bin.493) contained three linear sequences (contigs _2025, _2026 and _2630) of similar depth, totalizing 4 Mb, and a 4-fold more abundant circular sequence of 661 kb (contig_2905). A portion of the later displayed >99.9% to a *Pseudosulfitobacter pseudonitzschiae* plasmid; it was thus renamed MAG_104 and excluded from this bin. The other three were kept in a bin renamed MAG_06, scoring 93% completeness in terms of checkm markers for *Rhodobacteraceae* and very limited contamination.

Putative poorly-assembled bacteria: Planctomycetota and Alphaproteobacteria

Content of smaller bins was also checked using Checkm, revealing 4 poorly assembled bacterial metagenomes (each 15-25 contigs and 1-2.8 Mb), with completeness ranging 10-70% and low contamination scores (Suppl. Figure S9; Suppl. Table ST9). In the attempt of forming more complete MAGs, complementarity of the marker genes found in these small bins was assessed, but all combinations yielded overlaps in marker sets, increasing contamination rather than completeness scores.
The inferred taxonomy for these incomplete MAGs suggests that 4 additional bacterial taxa may populate the phycosphere at relatively lower abundance and/or high species diversity that hindered chromosome-level assembly.

- MAG_228 (bin.228) relates to the phylum Planctomycetota (likely within the order Phycisphaerales), which was found abundant in 16S analyses (Figure 4). It has been proposed that Planctomycetes species are relatively less documented than other bacterial phyla owing to their metabolic interplay with host cells [11].
- MAG_66 (bin.66) likely relates to genus ***Mameliella***, another Roseobacteraceae (together with the well-assembled MAG_04, MAG_05 and MAG_06); in 16S analyses of early cultures, Roseobacteraceae generally dominated over other Alphaproteobacteria.
- MAG_369 (bin.369) relates to the genus *Lentilitoribacter* (**Hyphomicrobiales**); this and other members of this order were among the Alphaproteobacteria found in uniform low abundance in 16S sequencing.
- MAG_169 (bin.169) relates to the genus *Nisea* (**Rhodospirillales**), not found in 16S analyses but close to the genus *Marivibrio*, which was the predominant Pseudomonadota in late cultures.

Genome annotation and functional prediction

Initial BRAKER3/TSEBRA runs produced fewer gene models; parameter sets were tested and the final annotation (34,225 transcripts) used intron_support=0.2 and sta_sto_support=0.5. Disabling --filter_single_exon_genes increased gene counts without improving BUSCO and was not used.

Computational prediction of potential trophic phagocytotic, photosynthetic, and prototrophic capabilities of *C. leptoporus* was assessed using a gene-based model: predictTrophicMode[4]. The code is available at https://github.com/burnsajohn/predictTrophicMode and was run in the default mode after scoring the test genome against the hidden Markov models (HMMs) that forms the core of the predictTrophicMode tool. Model predictions were visualized by projecting phagocytosis, photosynthesis, prototrophy, and the complementary "absence" dimension into color space (scripts adapted from the R package pavo) and displayed on a 2D circular Mollweide projection[5].

Metabolic model

A metabolic reconstruction of the *C. leptoporus* was generated by parsing the genome annotations through the KEGG and IUBMB databases [12,13]. The resulting dataset comprised reactions, genes, metabolites, and pathways spanning major metabolic processes, including fatty acid and lipid metabolism, amino acid metabolism, carbohydrate and energy metabolism, nucleotide metabolism, vitamin metabolism, and the biosynthesis of secondary metabolites.

The draft network was assembled into a systems biology model (SBML Level 3, XML format) using LibSBML, COPASI, and COBRApy [14-15]. The metabolic reconstruction consisted of 2,102 reactions, 478 metabolites, 460 genes, and 129 annotated pathways. Additional manual refinements were performed using COPASI and MetaNetX, leading to further improvements in consistency and annotation [15,17] and to enable interoperability with multiple databases such as KEGG, MetaCyc, and BiGG [12,18,19].

Finally, a *C. leptoporus* metabolic map was created with the Escher tool and the simulated SBML metabolic model [20].

The reconstructed *C. leptoporus* metabolic model is available in the BioModels and PatMeDB repositories [21,22].

Details on taxonomic assignments of bacterial MAGs

Taxonomic assignments of bacterial MAGs were performed using GTDB-Tk v2.3.2[6], which assigns taxonomy based on a concatenated set of 120 bacterial and 53 archaeal marker genes and places genomes into a reference tree derived from the Genome Taxonomy Database (GTDB release 207). A species-level phylogenetic tree including all MAGs was generated using KBase SpeciesTreeBuilder v0.1.4. The resulting tree was visualized with FigTree v1.4.4 and graphically refined in CorelDraw. The bacterial MAGs were named according to their closest phylogenetic relatives, followed by a strain identifier "CL_" indicating the isolate number (e.g., *Balneola* sp. CL_03).

Bacterial MAGs were annotated using the kb_DRAM tool[7], which generated nucleotide and protein sequence predictions along with functional annotations. Kyoto Encyclopedia of Genes and Genomes (KEGG) orthology (KO) identifiers were grouped into pathway categories based on KEGG annotations (accessed 2024–2025)[8]. KO profiles were analyzed and

visualized using the R package tidyverse (https://www.tidyverse.org/). Additional annotations were performed using GhostKOALA[9] and supplemented by homology-based searches, including BLAST and multiple sequence alignment with MAFFT.

Details on exclusion of uncompleted bacterial MAGs

In addition to the six high-quality MAGs, several bins exhibited low completeness and were excluded from downstream analyses. MAG_103 and MAG_105 aligned most closely with *Pleionea mediterranea* and *Roseovarius mucosus*, respectively, suggesting the presence of additional low-abundance taxa. Six other partial bins (25–75% completeness, <2% contamination) may represent additional phycosphere members (Table S4). Even smaller bins (<0.5 Mb, <5% completeness) were excluded due to insufficient assembly quality. No other primary producers were detected in the metagenome, indicating that *C. leptoporus* was the sole carbon fixer in the system.

Details on vitamin biosynthesis gene phylogenies methods

For pantothenate, the *C. leptoporus* panBC sequence was searched against a composite Tree of Life sequence library using a relaxed E-value threshold of 1×10^{-5} for initial candidate recovery. Retrieved sequences were reciprocally searched against the *C. leptoporus* proteome, and only candidates recovering the initial panBC query as the best hit were retained as potential homologues. Candidate sequences were further screened for PanBC-associated Pfam domains (PF02569 and PF02458) using HMMER/hmmsearch with relaxed thresholds of 1×10^{-15} and 1×10^{-10}, respectively, and classified as containing both domains, a single domain, or neither domain. Domain-containing sequences were aligned with MAFFT (--auto), trimmed with trimAl (-gt 0.5), and inspected in Geneious v10.0.9 using preliminary neighbour-joining trees with 100 bootstrap replicates to remove poorly aligned sequences with <50% overlap to the corresponding *C. leptoporus* domain or likely contaminants based on phylogenetic placement. Curated alignments were analysed with IQ-TREE v2. Retained sequences showed strong Pfam-domain support for both domains, with mean $-\log_{10}$ E-values of 94.5 ± 8.81 and 105.4 ± 10.93. Complete sequences, annotations, inclusion/exclusion rationale, alignments, and tree topologies are provided in Supplementary Dataset S1.

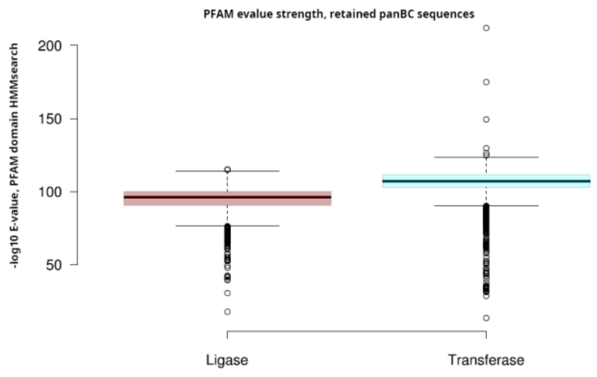

Domain conservation in panB/ C homologues. -log10 PFAM evalue of ligase domains (PF02569, 3404 retained sequences) and pantoate transferase (PF02458, 6156 sequences) identified by reciprocal BLAST best-hit search of the *Calcidiscus leptoporus panBC* sequence, alignment and manual cleaning. Most retained sequences show strong homology to each domain (pantoate ligase: -log10 mean 94.5± 8.81; pantoate synthetase: -log10 mean 105.4 ± 10.93), indicating strong domain conservation.

For biotin homologues, candidate sequences retained after BLASTp searches were inspected in the corresponding amino-acid alignments, and poorly aligned or strongly truncated sequences were excluded before concatenation. Query/reference coverage was calculated from the final retained alignments using the corresponding Balneolaceae CL03 protein as the reference for each gene partition. Across the five biotin protein partitions, coverage ranged from 62.17% to 100%, with median coverage values of 95.51–98.08% per partition. Pairwise amino-acid identity to the corresponding Balneolaceae CL03 reference protein ranged from 31.37–82.61% for bioF, 19.53–78.70% for bioK, 13.65–56.08% for bioC, 24.14–66.99% for bioD, and 33.66–80.81% for bioA.

2. Results

<u>*Detail vitamin pathways results*</u>

Vitamin B1.

A complete thiamine pathway was identified in *M. adhaerens* CL01 and *M. goseongensis* CL02 (Figure 3, Figure S10). *De novo* synthesis requires precursor formations by (i) *ThiC* and (ii) the *ThiG*-dependent thiazole pathway. This is then followed by coupling by *ThiE* (often a *ThiDE* fusion) and phosphorylation by *ThiD* and ThiL, with additional enzymes contributing downstream for salvage. In *M. adhaerens* CL01, *ThiC* appeared disrupted due to local assembly artefacts. Other MAGs encoded only partial salvage pathways. The *C. leptoporus* nuclear genome lacks *ThiC*, which prevents *de novo* thiamine synthesis; while the presence of downstream enzymes points to dependence on salvage/recycling of this vitamin.

Vitamin B2.

All members of the holobiont encoded a complete riboflavin pathway, with the sole gap being a hydrolysis step for which no prokaryotic enzyme is known (Figure 3, Figure S10).

Vitamin B3.

Marinobacter strains CL01 and CL02 encoded the full *nadABC* operon for *de novo* nicotinate biosynthesis (Figure 3, Figure S11). In contrast, *Balneolaceae* CL03 lacked *nadB*, while all three Rhodobacterales MAGs (CL04–CL06) and *C. leptoporus* lacked the complete gene set, indicating dependence on salvage pathways. The *NaMN permease PnuC*, which mediates uptake of nicotinate mononucleotide from the environment, was identified in *Balneolaceae* CL03 and *P. pseudonitzschiae* CL04. A *niaP*-like transporter, possibly involved in nicotinate uptake, was detected *in C. leptoporus* (gene model anno1.g6394.t1).

Vitamin B5.

With the exception of *P. pseudonitzschiae* CL04, every MAG encoded a complete pantothenate (B_5) biosynthesis pathway, including *panB* and *panC*. (Figure 3, Figure S13). Despite this upstream gap, *P. pseudonitzschiae* CL04 retained the downstream coenzyme A (CoA) pathway, and all six MAGs encoded the full complement of CoA biosynthetic enzymes. In *C. leptoporus* we found that a single gene encoding fused *panB/panC* domains, supported by intron presence and genomic context completes the pantothenate pathway. Additionally, alternative KO assignments for several steps (*panK*, COASY) indicate a complete coenzyme A (CoA) biosynthetic pathway.

We further performed a comparative genomic survey across 5,728 taxa and identified 1,678 species with both *panB* and *panC* homologues, but only 65 taxa with validated *panB/panC* fusions (Figure S13). These fusions were absent from bacteria, green algae, and diatoms and were confined to marine eukaryotes, including haptophytes, dinoflagellates, chromerids, apicomplexans, amoebozoa, and a few others. Phylogenies of the *panB* and *panC* domains placed fusion proteins together within clades, though not as a strictly monophyletic group. Within haptophytes, fusions occurred across major prymnesiophyte orders but were absent from *Pavlovophyceae*, which retained separate *panB* and *panC* genes.

Vitamin B6.

All six bacterial MAGs encoded the deoxyxylulose 5-phosphate (DXP)-dependent pathway for pyridoxal and pyridoxine biosynthesis (Figure 3, Figure S14). Apparent gaps in *pdxA* for *Balneolaceae* CL03 and *M. cryptomonadis* CL06 were resolved by targeted searches. The

upstream enzymes *epd* and *pdxB*, which catalyze early steps in the pathway, were also detected, albeit with scores slightly below standard thresholds. In *C. leptoporus*, the DXP-independent *pdxS–pdxT* pathway was present, indicating also the host's capacity for *de novo* B6 synthesis.

Vitamin B7.

M. adhaerens CL01 and *M. goseongensis* CL02 encoded the complete *bioC–bioH* pathway for biotin synthesis via fatty-acid intermediates (Figure 3, Figure S16). In contrast, all three *Rhodobacterales* MAGs (CL04–CL06) lacked biotin biosynthesis genes, indicating auxotrophy. Although KEGGs initially suggested that *Balneolaceae* CL03 lacked *bioC* and b*ioH*, GhostKOALA and manual curation identified a candidate operon containing *bioC* and a methylester esterase with a conserved catalytic triad, similar to cyanobacterial *bioK*, consistent with bioH function (Figure 3, Figure S17). Phylogeny and synteny support a horizontal transfer of this operon into Balneolota: the *bioF–bioK–bioC–bioD–bioA* cluster mirrors cyanobacterial gene order, and concatenated protein trees place CL03 with Cyanobacteria rather than with FCB bacteria (Supplementary Fig. S17). In contrast, *bioB* resides elsewhere in the Balneolota genomes and groups with typical FCB homologues. For uptake, the high-affinity biotin transporter *bioY* was present in Rhodobacterales MAGs, whereas the low-affinity *yigM* transporter was detected only in *M. adhaerens* CL01.

Vitamin B9.

All six MAGs and *C. leptoporus* encoded folate biosynthesis pathways (Figure 3, Figure S18). Among bacteria, *Roseovarius* sp. CL05 and *M. cryptomonadis* CL06 lacked the canonical alkaline phosphatases *phoA* or *phoD* involved in liberating folate precursors, however, *M. cryptomonadis* CL06 encoded *phoX*, an alternative phosphatase[10] not captured by KEGG. In the host genome, *FolB* was initially missed by KEGG annotation but identified as gene model anno1.g31798.t1.

The molybdenum cofactor (MoCo) pathway, which enables pterin-dependent redox enzymes, was missing in *C. leptoporus* and the *Balneolaceae* CL03, but present in all other bacterial MAGs.

Vitamin B12.

Only two Rhodobacterales strains—*P. pseudonitzschiae* CL04 and *Roseovarius* sp. CL05—encoded cobalamin biosynthesis (Figure 3, Figure S19). In *P. pseudonitzschiae* CL04, we detected a canonical *cobI–cobJ–cobK* aerobic cluster which, coupled with the absence of *cbiD* (an anaerobic-pathway gene), indicative of the aerobic biosynthesis pathway. By contrast, *Roseovarius* sp. CL05 lacks the O_2-dependent enzymes *cobF* and *cobG*, indicating the anaerobic route. The adenosyltransferase *pduO*, which activates environmental cobalamin, was present in all bacterial MAGs. The outer membrane cobalamin transporter *btuB* was detected in *M. adhaerens* CL01 and *M. goseongensis* CL02, whereas other uptake components (*btuCDF, cblT, cbrT*) were not detected. Only the *Marinobacter* MAGs encoded the cobalamin-independent methionine synthase *MetE*, implying reduced dependence on cobalamin.

In *C. leptoporus,* the presence of *pduO* indicates capacity to activate imported cobalamin; and together with the absence of *de novo* cobalamin biosynthesis genes and the presence of trafficking factors *bacA* and LMBRD1, this supports cobalamin auxotrophy, consistent with patterns reported for many haptophytes.

REFERENCES

1. Tillich M, Lehwark P, Pellizzer T, Ulbricht-Jones ES, Fischer A, Bock R et al. GeSeq—versatile and accurate annotation of organelle genomes. Nucleic Acids Res 2017;45:W6–W11. https://doi.org/10.1093/nar/gkx391

2. Chaux F, Agier N, Garrido C, Fischer G, Eberhard S, Xu Z. Telomerase-independent survival leads to a mosaic of complex subtelomere rearrangements in Chlamydomonas reinhardtii. Genome Res 2023;33:1582–98. https://doi.org/10.1101/gr.278043.123

3. Fulnečková J, Ševčíková T, Fajkus J, Lukešová A, Lukeš M, Vlček Č et al. A broad phylogenetic survey unveils the diversity and evolution of telomeres in eukaryotes. Genome Biol Evol 2013;5:468–83. https://doi.org/10.1093/gbe/evt019

4. Burns JA, Pittis AA, Kim E. Gene-based predictive models of trophic modes suggest Asgard archaea are not phagocytotic. Nat Ecol Evol 2018;2:697–704. https://doi.org/10.1038/s41559-018-0477-7

5. Jimenez V, Burns JA, Le Gall F, Not F, Vaulot D. No evidence of phago-mixotrophy in Micromonas polaris (Mamiellophyceae), the dominant picophytoplankton species in the Arctic. J Phycol 2021;57:435–46. https://doi.org/10.1111/jpy.13125

6. Chaumeil P-A, Mussig AJ, Hugenholtz P, Parks DH. GTDB-Tk v2: memory friendly classification with the Genome Taxonomy Database. Bioinformatics 2022;38:5315–16. https://doi.org/10.1093/bioinformatics/btac672

7. Shaffer M, Borton MA, Bolduc B, Faria JP, Flynn RM, Ghadermazi P et al. kb_DRAM: annotation and metabolic profiling of genomes with DRAM in KBase. Bioinformatics 2023;39:btad110. https://doi.org/10.1093/bioinformatics/btad110

8. Aramaki T, Blanc-Mathieu R, Endo H, Ohkubo K, Kanehisa M, Goto S et al. KofamKOALA: KEGG Ortholog assignment based on profile HMM and adaptive score threshold. Bioinformatics 2020;36:2251–52. https://doi.org/10.1093/bioinformatics/btz859

9. Kanehisa M, Sato Y, Morishima K. BlastKOALA and GhostKOALA: KEGG tools for functional characterization of genome and metagenome sequences. J Mol Biol 2016;428:726–31. https://doi.org/10.1016/j.jmb.2015.11.006

10. Sebastian M, Ammerman JW. The alkaline phosphatase PhoX is more widely distributed in marine bacteria than the classical PhoA. ISME J 2009;3:563–72. https://doi.org/10.1038/ismej.2009.10

11. Kaboré OD, Godreuil S, Drancourt M. Planctomycetes as host-associated bacteria: a perspective that holds promise for their future isolations, by mimicking their native

environmental niches in clinical microbiology laboratories. Front Cell Infect Microbiol 2020;10:519301. https://doi.org/10.3389/fcimb.2020.519301

12. Kanehisa M, Furumichi M, Sato Y, Ishiguro-Watanabe M, Tanabe M. KEGG: integrating viruses and cellular organisms. Nucleic Acids Res 2021;49:D545–D51. https://doi.org/10.1093/nar/gkaa970

13. International Union of Biochemistry and Molecular Biology. Recommendations on biochemical and organic nomenclature, symbols and terminology. 2022. https://iubmb.qmul.ac.uk/

14. Bornstein BJ, Keating SM, Jouraku A, Hucka M. LibSBML: an API library for SBML. Bioinformatics 2008;24:880–81. https://doi.org/10.1093/bioinformatics/btn051

15. Hoops S, Sahle S, Gauges R, Lee C, Pahle J, Simus N et al. COPASI—a COmplex PAthway SImulator. Bioinformatics 2006;22:3067–74. https://doi.org/10.1093/bioinformatics/btl485

16. Ebrahim A, Lerman JA, Palsson BO, Hyduke DR. COBRApy: COnstraints-Based Reconstruction and Analysis for Python. BMC Syst Biol 2013;7:74. https://doi.org/10.1186/1752-0509-7-74

17. Moretti S, Tran VDT, Mehl F, Ibberson M, Pagni M. MetaNetX/MNXref: unified namespace for metabolites and biochemical reactions in the context of metabolic models. Nucleic Acids Res 2021;49:D570–D74. https://doi.org/10.1093/nar/gkaa992

18. Caspi R, Altman T, Billington R, Dreher K, Foerster H, Fulcher CA et al. The MetaCyc database of metabolic pathways and enzymes and the BioCyc collection of pathway/genome databases. Nucleic Acids Res 2014;42:D459–D71. https://doi.org/10.1093/nar/gkt1103

19. Norsigian CJ, Pusarla N, McConn JL, Yurkovich JT, Dräger A, Palsson BO et al. BiGG Models 2020: multi-strain genome-scale models and expansion across the phylogenetic tree. Nucleic Acids Res 2020;48:D402–D6. https://doi.org/10.1093/nar/gkz1054

20. King ZA, Dräger A, Ebrahim A, Sonnenschein N, Lewis NE, Palsson BO. Escher: a web application for building, sharing, and embedding data-rich visualizations of biological pathways. PLoS Comput Biol 2015;11:e1004321. https://doi.org/10.1371/journal.pcbi.1004321

21. Malik-Sheriff RS, Glont M, Nguyen TVN, Tiwari K, Roberts MG, Xavier A et al. BioModels—15 years of sharing computational models in life science. Nucleic Acids Res 2020;48:D407–D15. https://doi.org/10.1093/nar/gkz1055

22. Bannerman BP, Oarga A, Júlvez J. Mycobacterial metabolic model development for drug target identification. GigaByte 2023;2023:gigabyte80. https://doi.org/10.46471/gigabyte.80

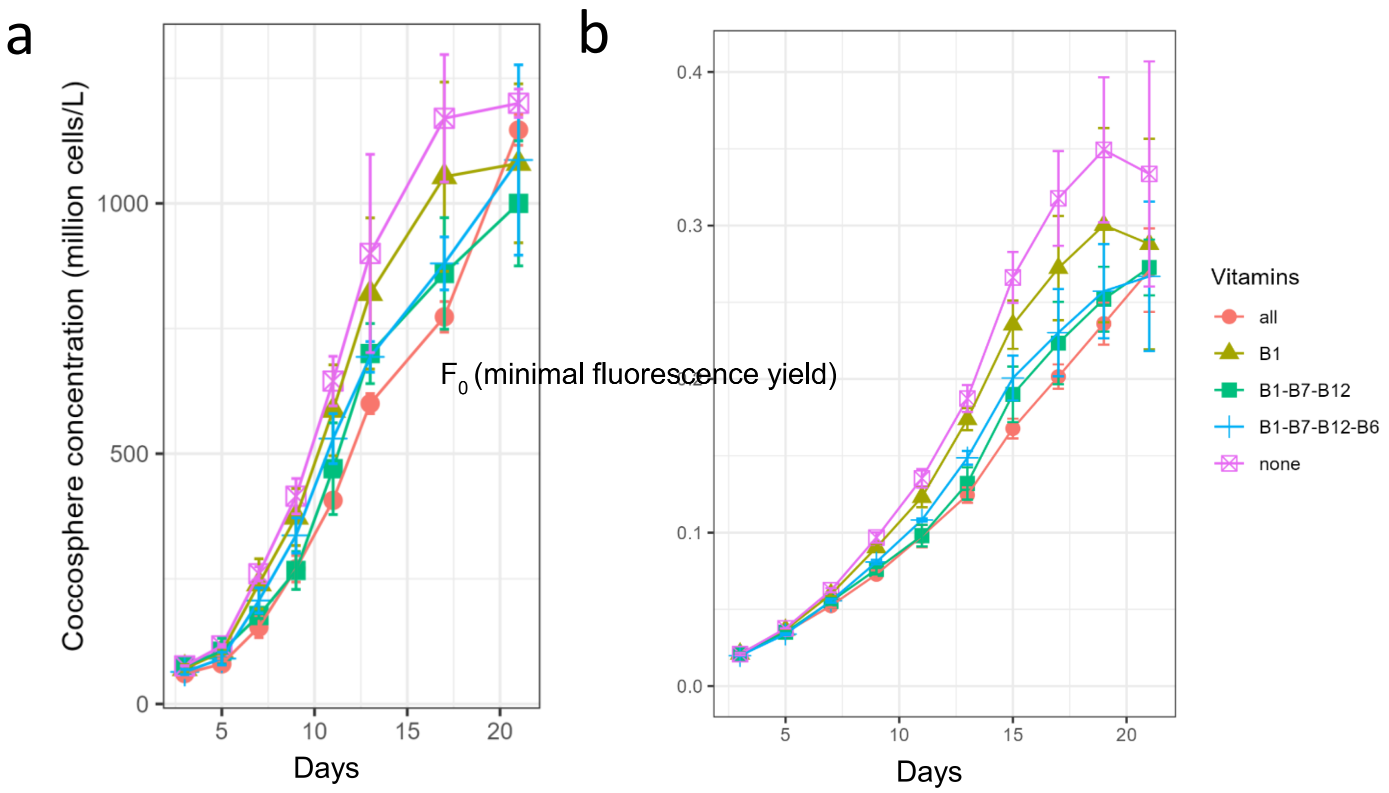

Figure S1.
Comparison of cell counts vs F_0 (minimal fluorescence yield) of *Calcidiscus leptoporus* cultures.

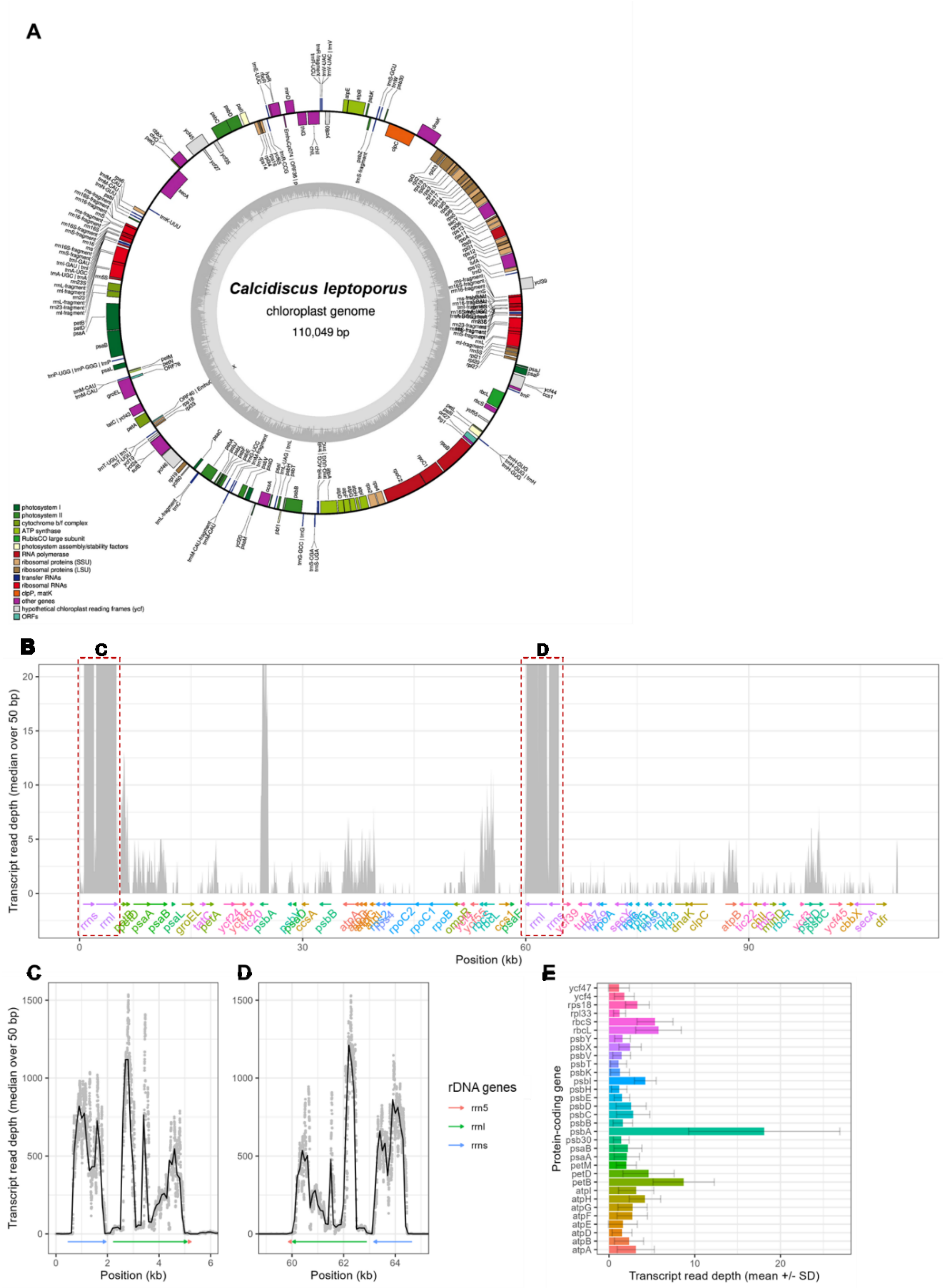

Figure S2.

The chloroplast genome of *Calcidiscus leptoporus*.

A. The contig MAG_1001 was annotated using online GeSeq tool (Tillich et al. 2017) (https://chlorobox.mpimp-golm.mpg.de/geseq.html, parameters: Plastid (algae); Annotate IR; Keep best annotation only; Protein search identity: 25; rRNA, tRNA, DNA search identity:25; Annotate: CDS, tRNA, rRNA; ARAGORN v1.2.38; tRNAscan-SE v2.0.7; BLAT reference sequences: haptophyte plastids). The initial contig (contig_3648) was edited to start with the small ribosomal RNA (16S).

B-E. RNA-seq data (SRR1300371; Keeling et al., 2014) were mapped to the plastid genome to compute expression level. Read depth is shown for rRNA clusters (C,D) and the most expressed protein-coding genes (E). High expression of *psbA* likely reflects the sustained synthesis of the photosystem II subunit D1, generally observed in plastid transcriptomes (Janouškovec et al, 2013), and is primarily damaged by photoinhibition and continuously replaced.

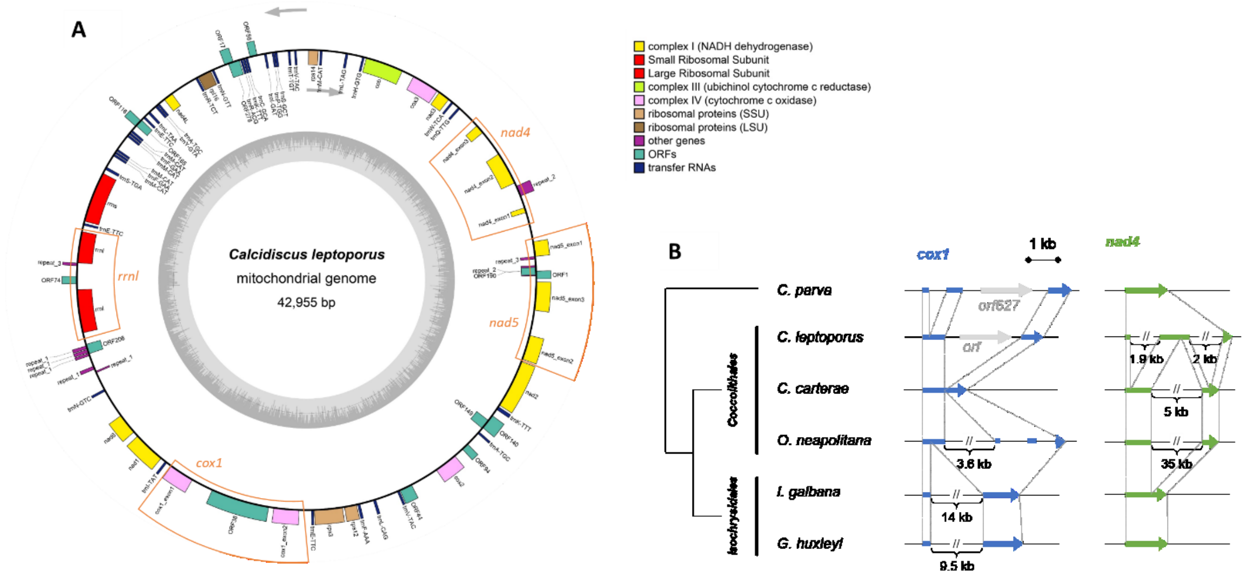

Figure S3.

The mitochondrial genome of *Calcidiscus leptoporus*.

A. The contig MAG_1002 was annotated using online GeSeq tool (Tillich et al. 2017) (parameters: Mitochondrial; Keep best annotation only; Protein search identity: 25; rRNA, tRNA, DNA search identity:25; Annotate: CDS, tRNA, rRNA; ARAGORN v1.2.38; tRNAscan-SE v2.0.7; BLAT reference sequences: haptophyte mitogenomes). The initial contig (contig_3646), which was an 86-kb tandem repeat representing two identical copies of mitochondrial genome, was simplified to 43kb and the annotation file was manually curated to account for the trans-splicing of *cox1*, *nad4* and *nad5* and rRNA.
B. Structure of trans-spliced genes *cox1* and *nad5* among haptophytes.

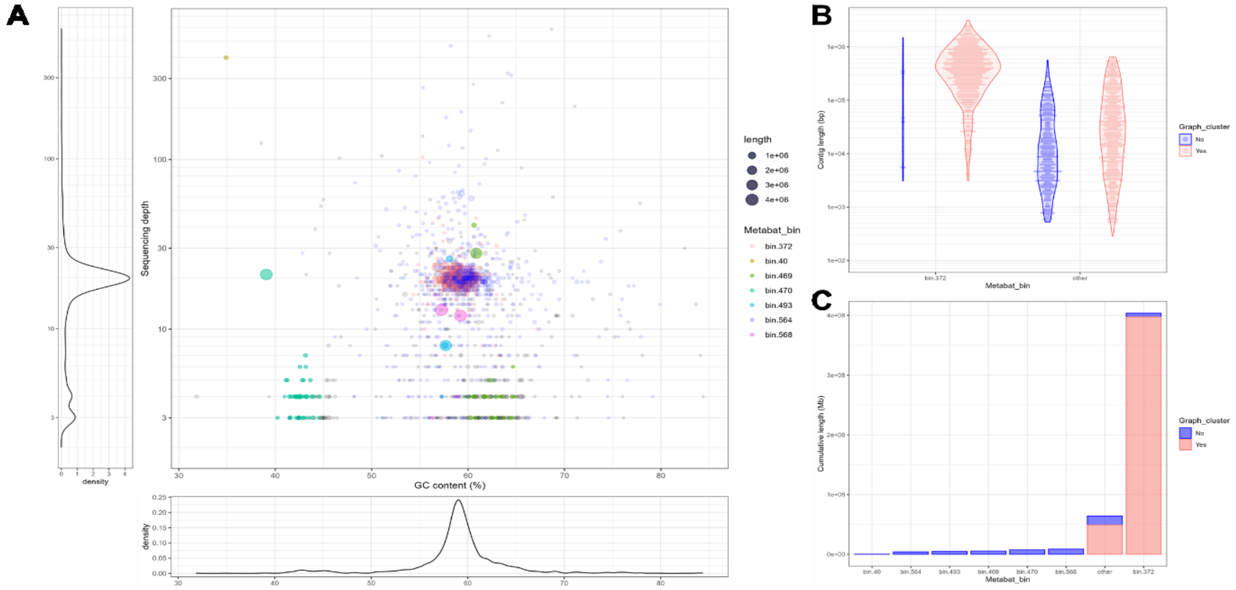

Figure S4.

Contigs distribution and initial metagenome binning.

Contigs were binned using Metabat (initial binning) before manual curation.

A. Contig sequencing depth (y-axis, log scale) and GC content (x-axis, %). Contig length (in bp) is depicted by data point size. Colors according to Metabat binning (main bins). Dark blue data points depict contigs represented in the graph assembly cluster (Fig. 1A) but absent from the bin.372. Black data points depict contigs absent from the main bins (unattributed bins). Subpanels display overall distributions of sequencing depth and GC content.

B. Length distribution (log scale) of contigs from bin.372 (*Calcidiscus)* and unbinned.

C. Cumulative length of contigs in the main Metabat bins. (In B and C, colors depict contigs represented in the graph assembly cluster).

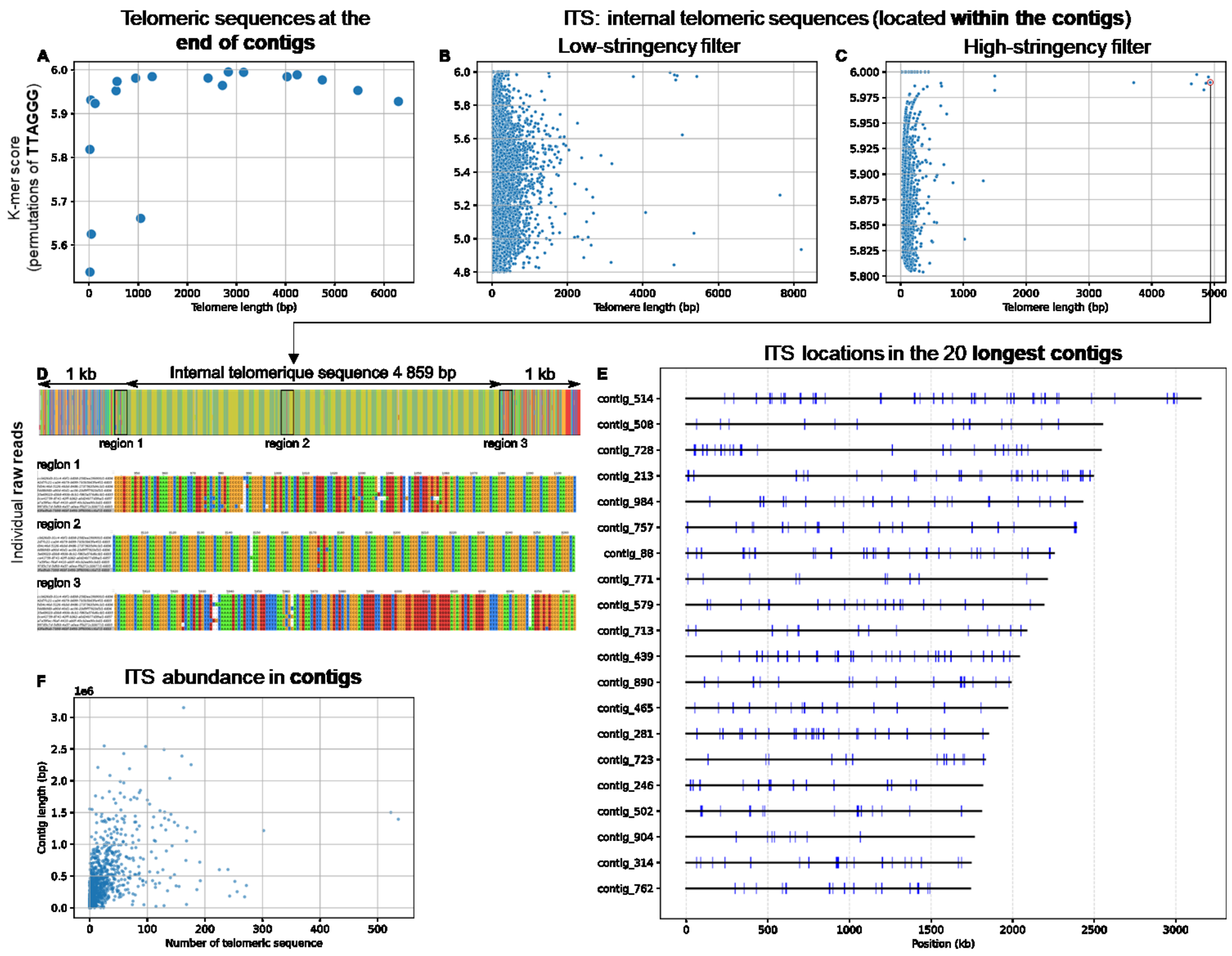

Figure S5.

Internal and terminal telomere sequences in *Calcidiscus leptoporus* genome.

A. Length and quality score of telomere sequences found at the end of the assembled contigs, which most likely represent terminal telomeres, retrieved using Teloreader. The K-mer score indicates the average deviation from the perfect motif and its circular permutations, with 6 representing a perfect sequence and 5.8 representing one mutation every 5 motifs on average.

B-C. Length and quality score of telomere sequences found within the assembled contigs (> 50 bp away from the extremities), i.e. internal telomeric sequences (ITS). (B) A lower threshold of 4.8 for the average quality score was used to detect the ITSs, allowing the inclusion of slightly degenerated telomere motifs. (C) With a more stringent lower threshold value of 5.8 for the average quality score, thus retrieving ITSs with near-perfect telomeric motifs. A few ITSs were longer than 1 kb, one of which is analyzed further in (D).

D. Inspection of raw Nanopore reads to verify that an ITS of ~5 kb did not correspond to a misassembled telomere or another artifact. We found several reads (9 are shown) that traverse the ITS, thus supporting the assembly. The boundary regions (regions 1 and 3) and a central region (region 2) are presented at higher magnification.

E. The localizations of the ITSs detected in (C) are presented for the 20 longest contigs, showing that they are widespread within the genome.

F. The number of ITSs present in a contig correlates with the contig size. Identification of telomere motif and its distribution in the nuclear genome.

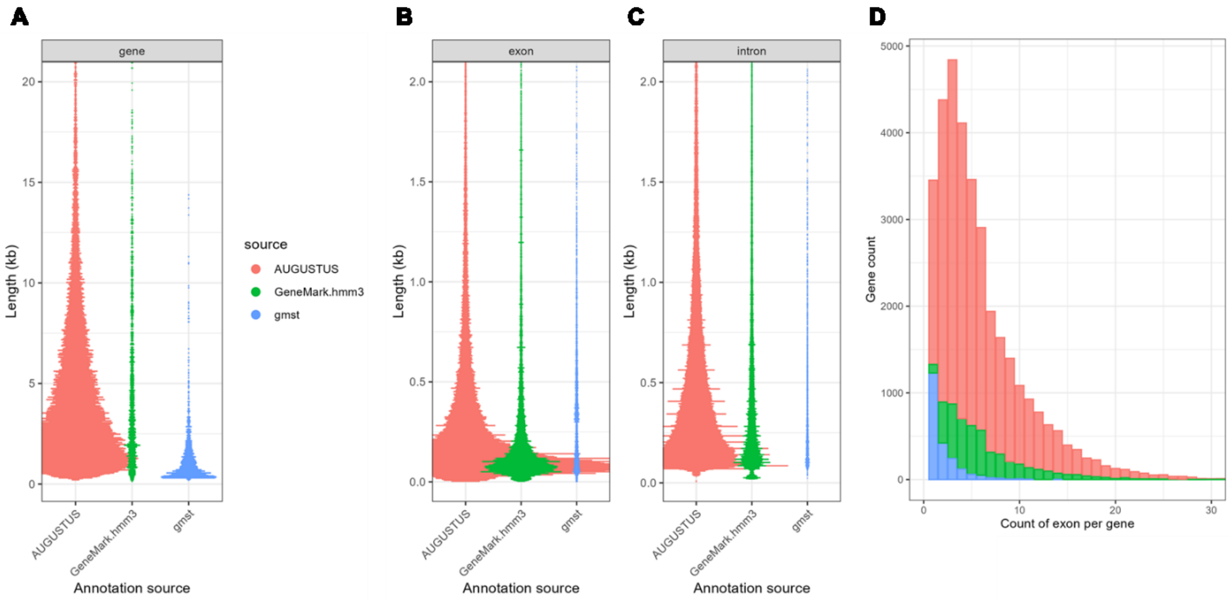

Figure S6.

Length distribution of genomic features.

The genome of *C. leptoporus* was annotated using Braker3 (Gabriel et al.2024) which encompasses 3 algorithm, shown on x-axis. Displayed are the length distribution of genes (A), exons (B) and introns (C), and count of exons per genes (D).

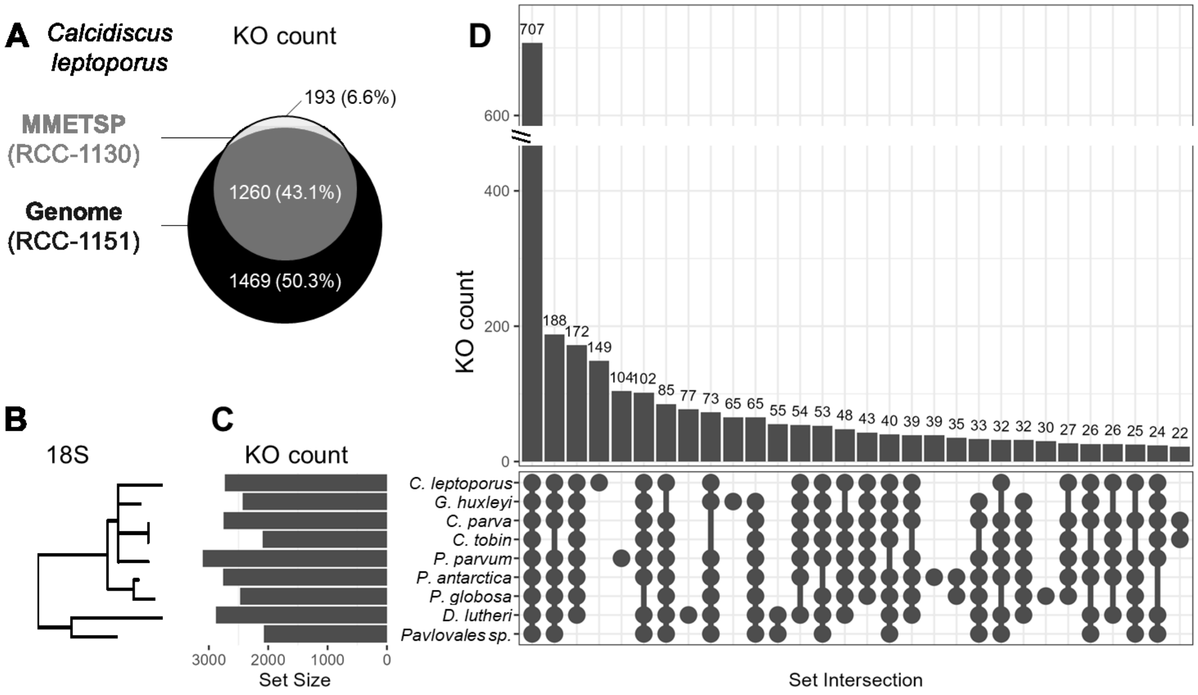

Figure S7.
KEGG orthology (KO) distribution.

A. Overlap of KOs in *C. leptoporus* between transcriptome-based (MMETSP) and genome-based (this work) assemblies.
B. Schematic phylogenetic distance between haptophyte species, based on 18S rDNA.
C. Total of KO counts in *C. leptoporus* and other haptophytes.
D. Overlap of KO found in haptophytes. Protein models were obtained from Phycocosm (https://phycocosm.jgi.doe.gov/phycocosm/home; accessed October 2024). Pparv12B1: *Prymnesium parvum*, Chrpa1 _1 *Chrysochromulina parva*; Chrsp _1: *Chrysochromulina tobin*; Pavlov2436: Pavlovales sp.); Emihu1: *Gephyrocapsa* (previously *Emiliania*) *huxleyi*; Phaant1: *Phaeocystis antarctica*; Phaglo: *Phaeocystis globosa*; Dialut1: *Diacronema* (previously *Pavlova*) *lutheri*). KO annotations were assigned using Kofamscan.

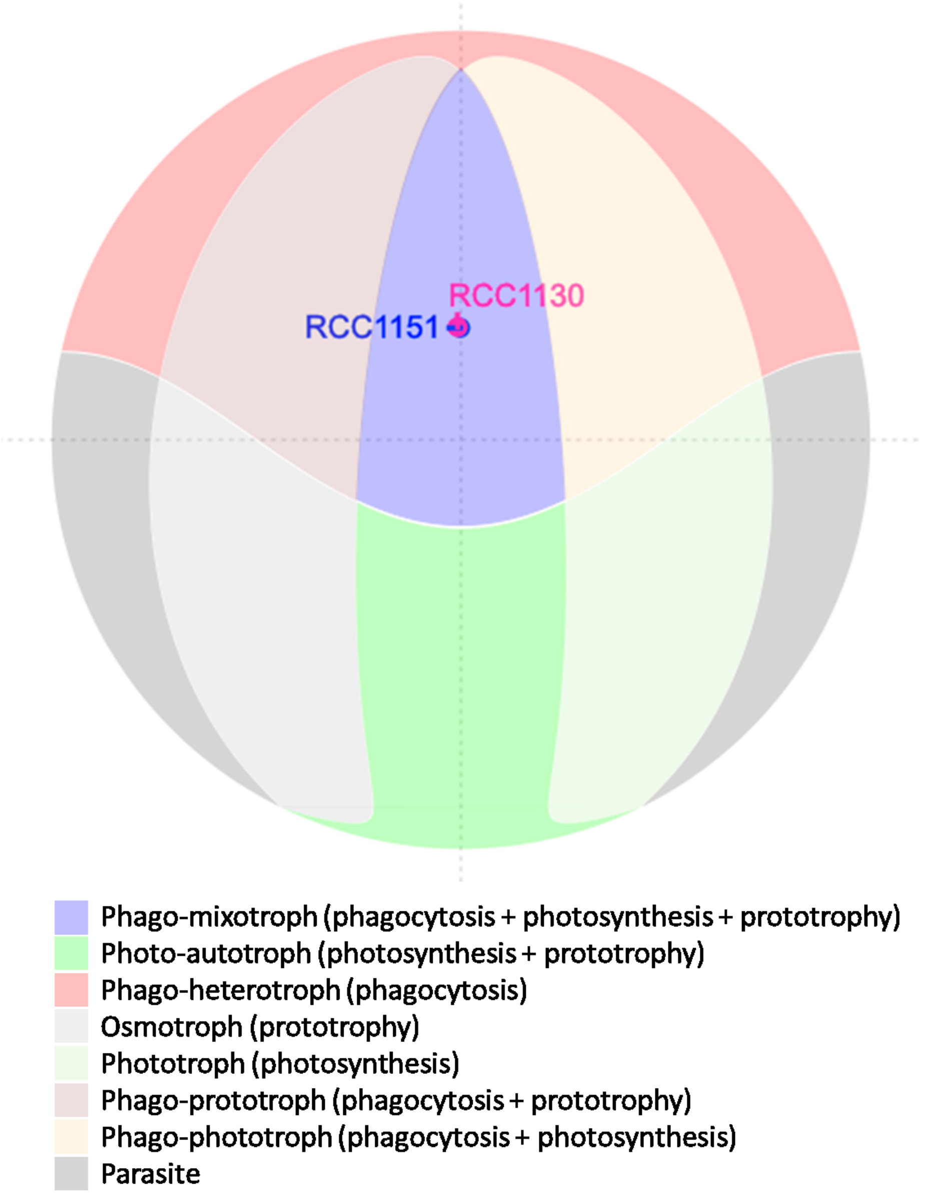

Figure S8.
Trophic mode predictions from *C. leptoporus* genome. Computational prediction of potential trophic phagocytotic, photosynthetic, and prototrophic capabilities of *C. leptoporus* was assessed using a gene-based model: predictTrophicMode (Burns et al. 2018, Jimenez et al. 2021 Book et al. 2021). The code is available at https://github.com/burnsajohn/predictTrophicMode and was run in the default mode after scoring the test genome against the hidden Markov models (HMMs) that forms the core of the predictTrophicMode tool. Model predictions were visualized by projecting phagocytosis, photosynthesis, prototrophy, and the complementary "absence" dimension into color space (scripts adapted from the R package *pavo*) and displayed on a 2D circular Mollweide projection (Jimenez et al. 2021).

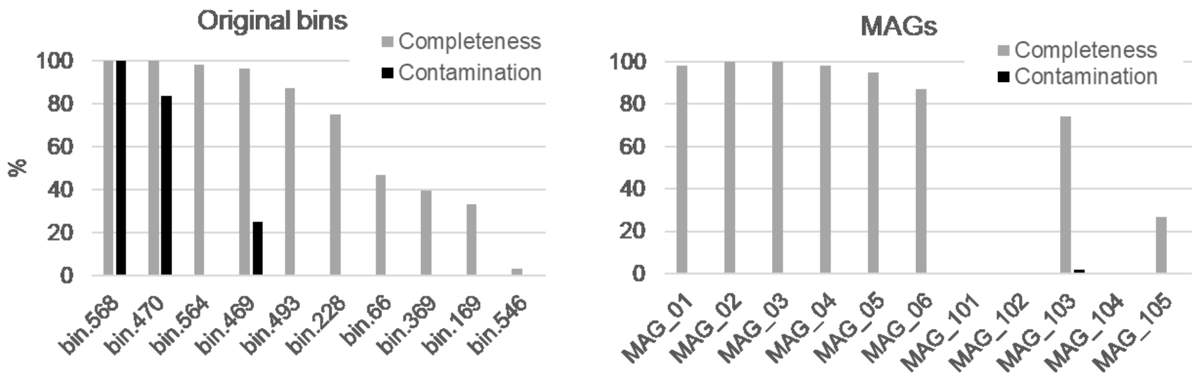

Figure S9.
Checkm assessment of bacterial metagenomes.

Completeness and contamination assessed at the kingdom level (“k__bacteria”).

A. Initial bins (Metabat2, Kang et al. 2019).
B. Final metagenome-assembled genomes (MAGs).

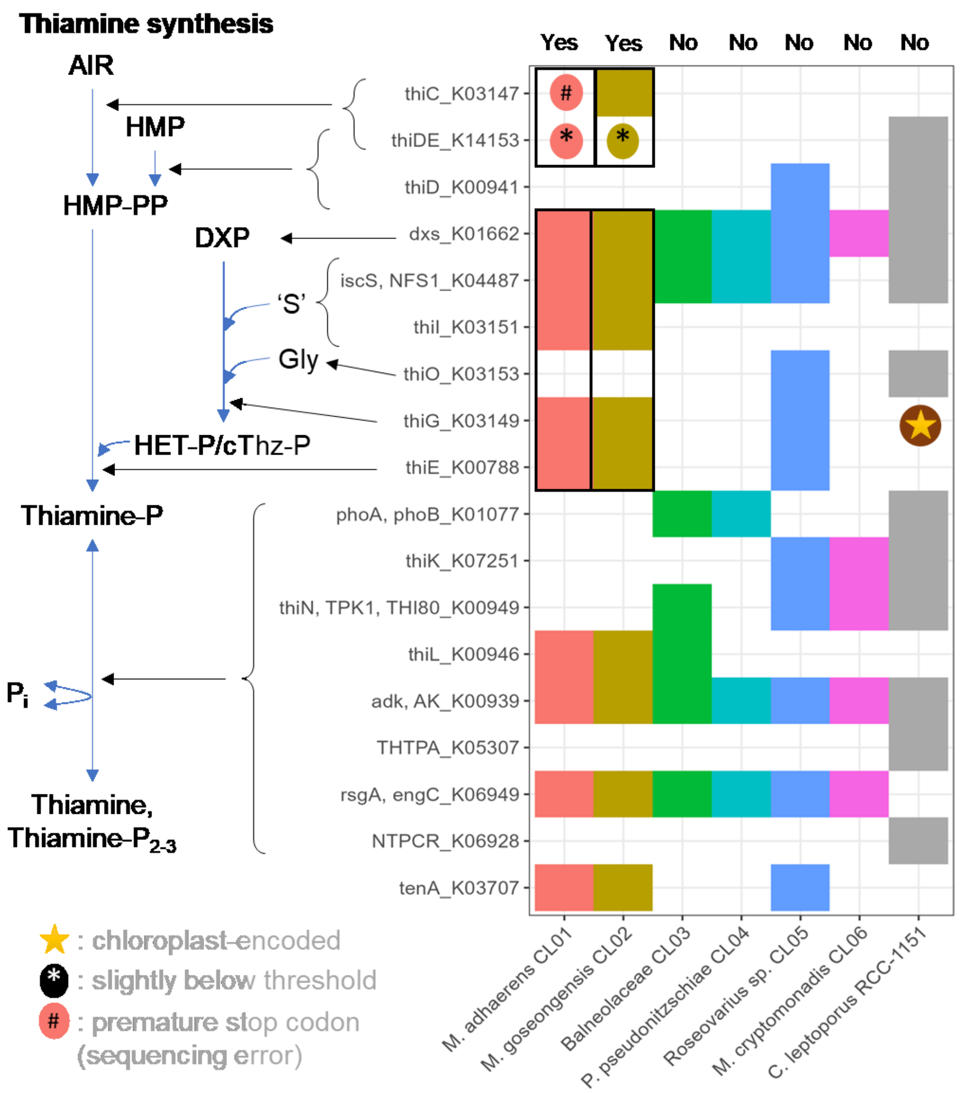

Figure S10.

Major steps of the thiamine (B1) biosynthesis .

Based on KEGGS map00730 and complementary literature (Croft et al. 2006, Helliwell 2017, Gutowska et al. 2017), the main intermediates and reactions (left) together with related genes and/or KO number (right) are presented. Colored tiles indicate detected KOs in the genomes and complete pathways are outlined in black. Star indicates a gene (*thiG*) absent from nuclear genome but found in the chloroplast genome. Blast searches and manual analyses of below-threshold KO scores allowed to retrieve missing genes indicated by "*" and "#". By comparison to *Marinobacter adherens* reference genome (NZ_CP076686.1), the premature stop codon found in this genome is assumed to derive from an assembly error. **AIR**: Aminoimidazole ribotide; **HMP**: 4-Amino-5-hydroxymethyl-2-methylpyrimidine; **HMP-PP**: HMP diphosphate; **DXP**: 1-Deoxy-D-xylulose 5-phosphate; **'S'**: Sulfur-carrier protein; **Gly**: Iminoglycine; **HET-P**: 4-Methyl-5-(2-phosphonooxyethyl)thiazole; **cThz-P**: 4-Methyl-5-[2-(phosphonooxy)ethyl]-1,3-thiazole-2-carboxylate; **Thiamine-P(P_2,P_3)**: Thiamine mono-(di-, tri-)phosphate, P_i: phosphate.

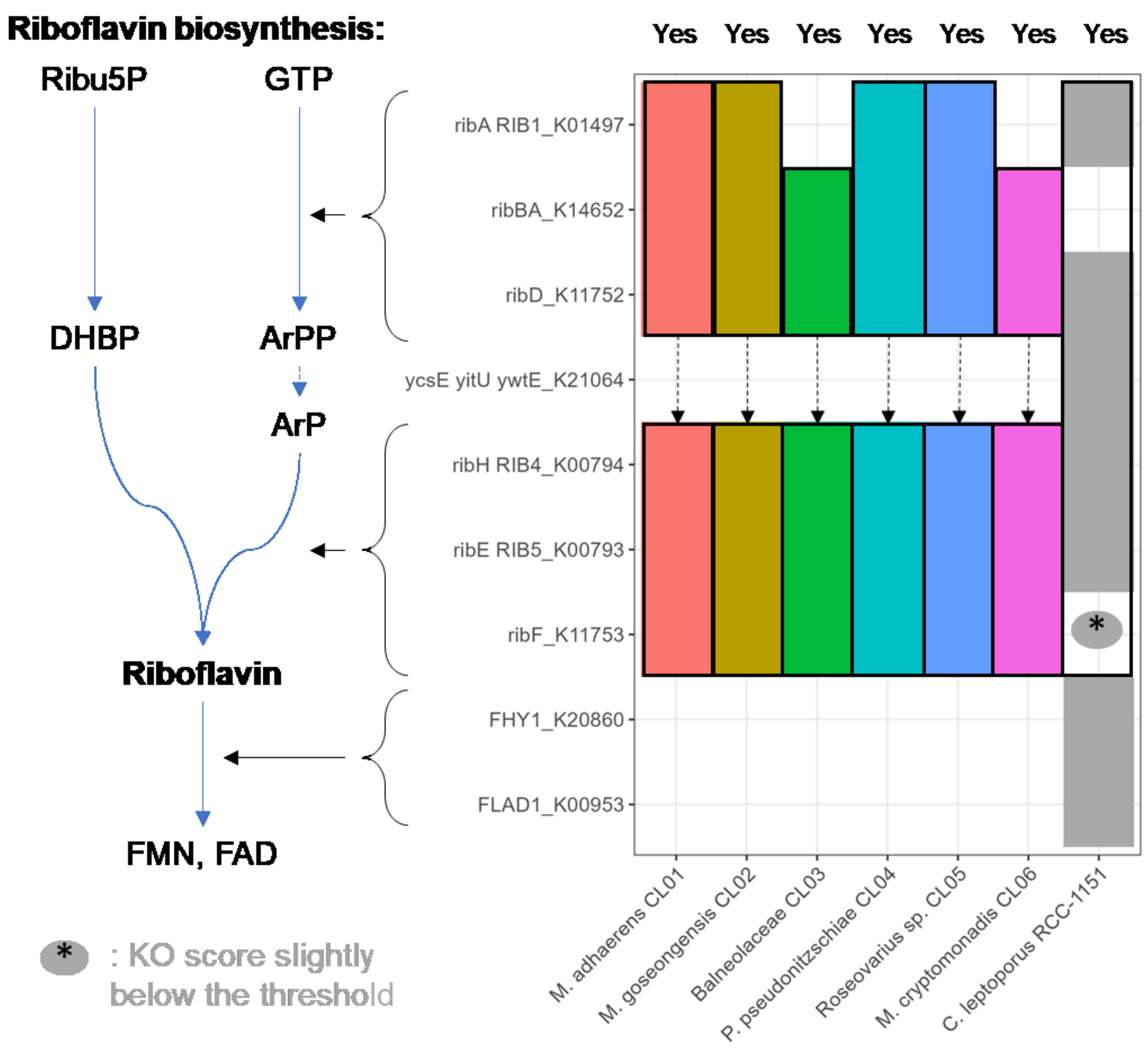

Figure S11.

Major steps of the riboflavin (B2) biosynthesis.

Based on KEGGS map00740 and complementary literature (Cummings et al. 2014, Sa et al. 2016, Averianova et al. 2020), the main intermediates and reactions (left) together with related genes and/or KO number (right) are presented. Colored tiles indicate detected KOs in the genomes and complete pathways are outlined in black. Blast searches and manual analyses of below-threshold KO scores allowed to retrieve missing genes indicated by "*". In *C. leptoporus* the putative riboflavin kinase ribF/RFK (K11753/K00861) is encoded by anno1.g60456.t1. The specific ArPP phosphatase is still unknown and shown as dotted arrow. **ArPP**: 5-Amino-6-(5'-phospho-D-ribitylamino)uracil; **ArP**:5-Amino-6-(D-ribitylamino)uracil; **Ribu5P**: D-Ribulose 5-phosphate; **GTP**: Guanosine triphosphate; **DHBP**: 3,4-Dihydroxybutan-2-one 4-phosphate; **FMN**: Flavin mononucleotide; **FAD**: Flavin adenine dinucleotide.

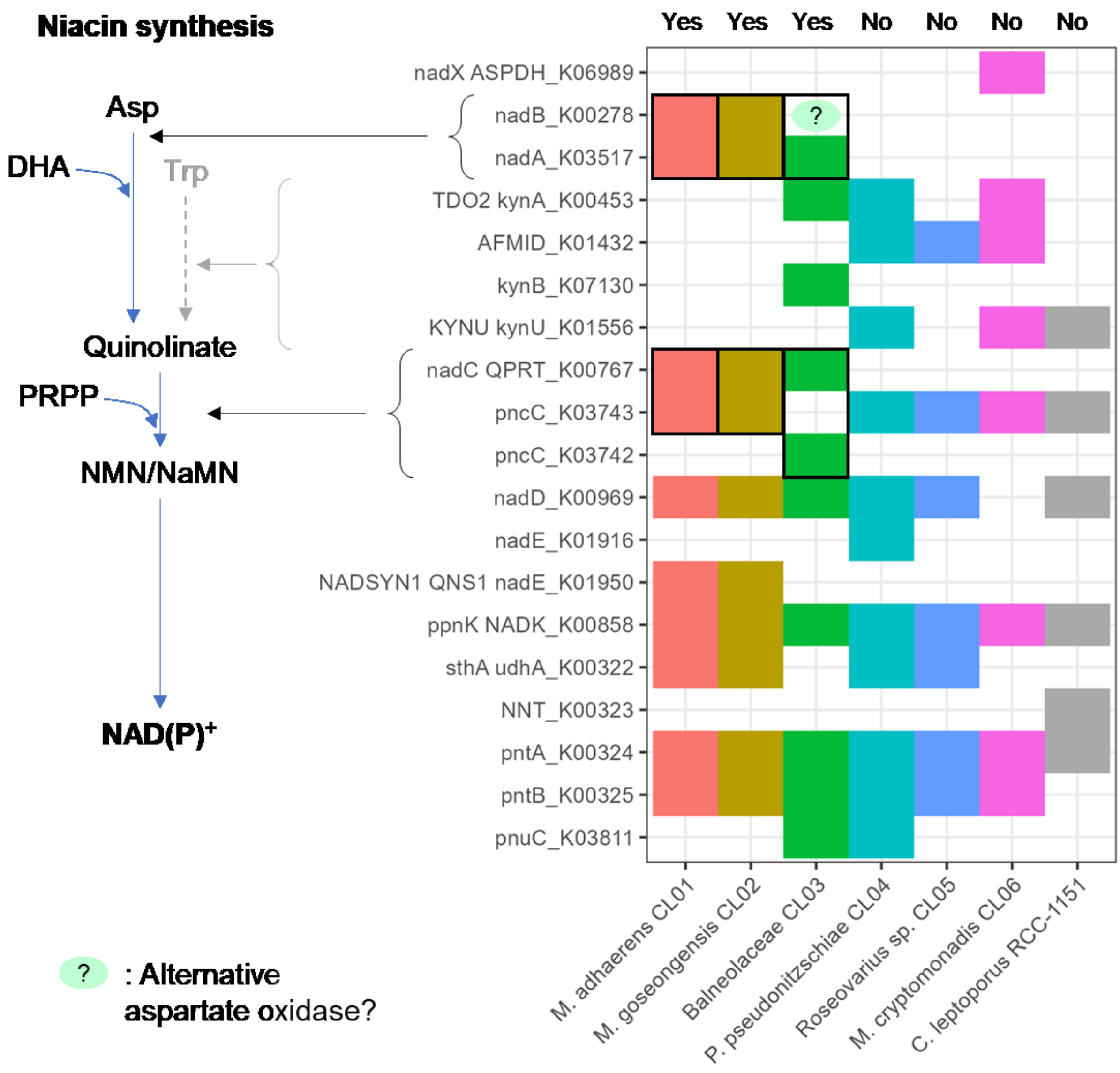

Figure S12.

Major steps of the niacin (B3) biosynthesis.

Based on KEGGS map00760 and complementary literature (Roussin & Salcedo 2021), the main intermediates and reactions (left) together with related genes and/or KO number (right) are presented. Colored tiles indicate detected KOs in the genomes and complete pathways are outlined in black. In grey is an alternative synthesis of quinolinate. The symbol "?" denotes a putative alternative aspartate oxidase: blast searches and manual analyses of below-threshold KO scores excluded presence of *nadB* but point to a related oxidase (contig_3314_1444), annotated as succinate dehydrogenase. **Asp**: L-aspartate, **Trp**: Tryptophan; **DHA**: Dihydroxyacetone phosphate; **PRPP**: 5-Phosphoribosyl diphosphate; **NaMN**: nicotinic acid mononucleotide; **NMN**: nicotinamide mononucleotide ; **NAD(P)**: Nicotinamide adenine dinucleotide (phosphate).

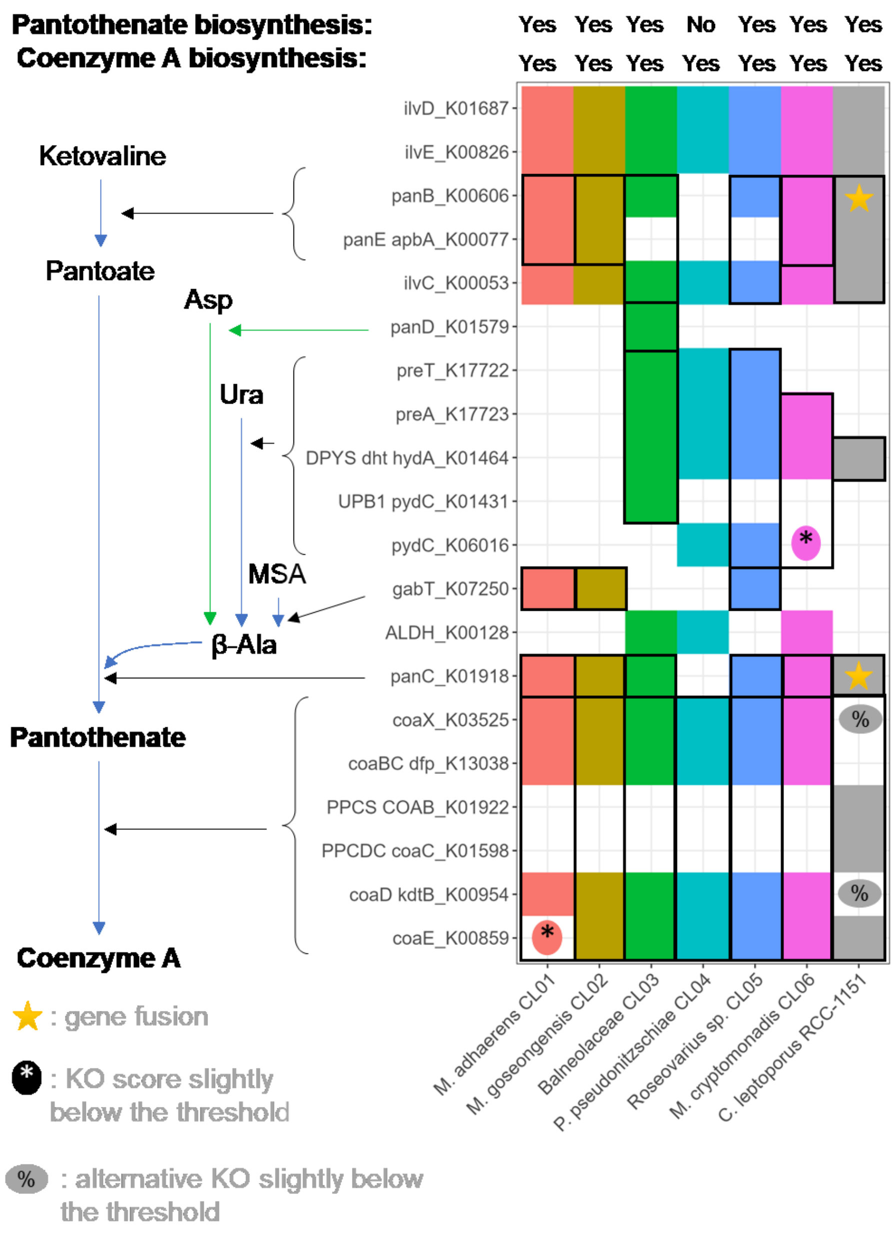

Figure S13.

Major steps of the pantothenate (B5) and coenzyme A biosynthesis.
Based on KEGGS map00770 and complementary literature (Leonardi and Jackowski 2007, Merkamm et al. 2003), the main intermediates and reactions (left) together with related genes and/or KO number (right) are presented. Colored tiles indicate detected KOs in the genomes and complete pathways are outlined in black. In green is an alternative synthesis of β-Ala used by the Balneolaceae strain CL03. Blast searches and manual analyses of below-threshold KO scores allowed to retrieve missing genes indicated by "*". In the *C. leptoporus* genome, panK_K09680 (anno1.g23784.t3) instead of CoaX, and COASY_K02318 (anno1.g55330.t1) instead of coaD were detected slightly below the threshold (marked by "%"). The stars point to a single gene which displays both panB and panC domains. **Asp**: L-aspartate, **Ura**: Uracil, **MSA**: Malonate semialdehyde; **β-Ala**: beta-Alanine.

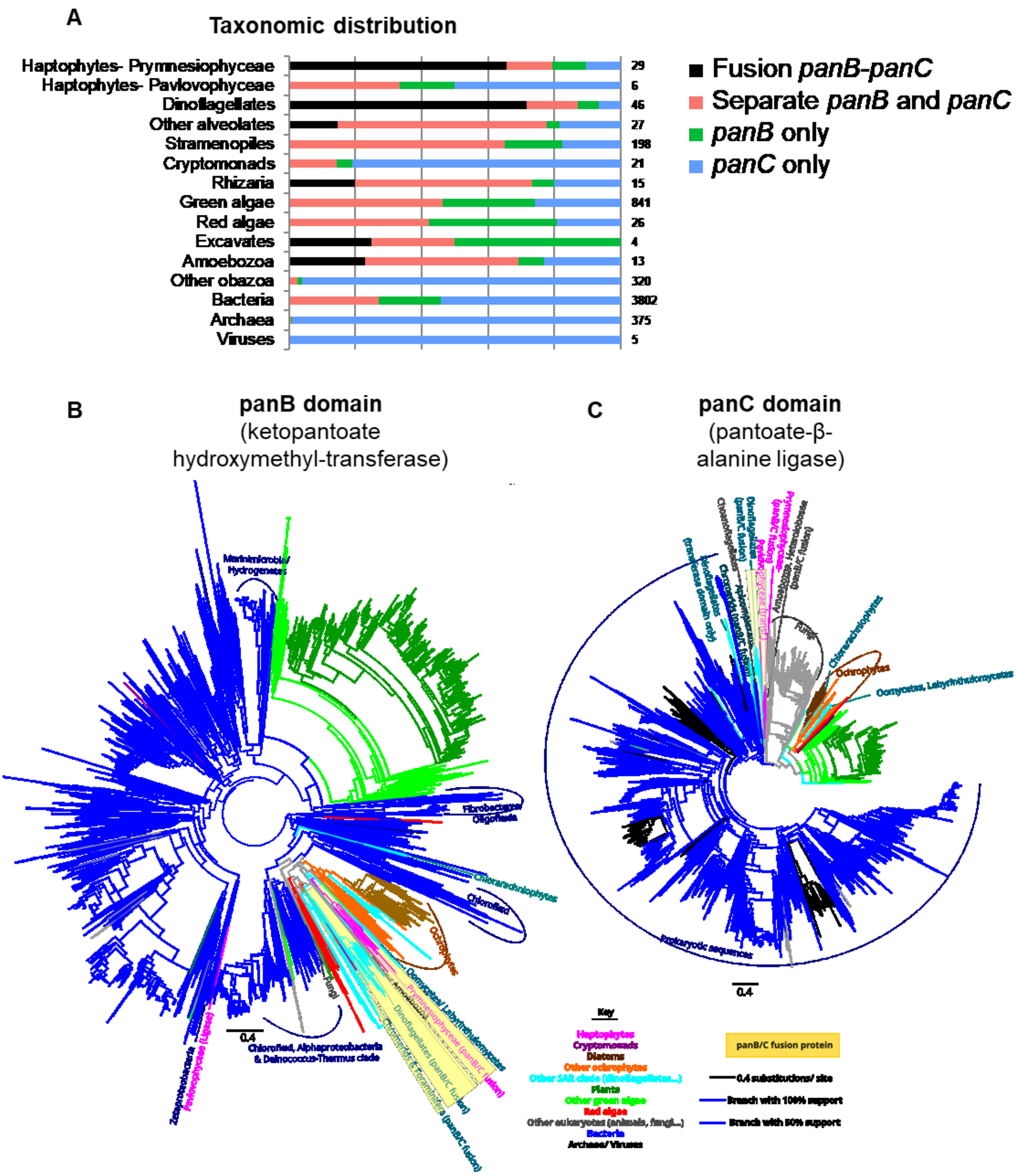

Figure S14.

The gene fusion of *panB* and *panC* domains displays an intricate distribution among eukaryotes.

Homologues to *panB* and *panC* proteins were identified by reciprocal BLASTp/BLASTp best-hit search of the *C. leptoporus panBC* fusion peptide sequence, PFAM annotation, alignment and single-gene tree across a composite library of uniref, jgi Phycocosm, onekp and MMETSP sequences, following Dorrell et al (2024).

A. Bar charts of the relative number of species for which *panB* and *panC* homologues were identified as a verified *panB panC* gene fusion (black), separate *panB* and *panC* genes (red) or only *panB* (green) or *panC* (blue) sequences were detected. Species are arbitrarily split by

taxonomy to highlight clades in which *panB/C* fusions are detected. The total number of species with at least one *panB* or *panC* homologue are shown on the right hand side.
B, C. Consensus IQtree topologies of a curated 3433 branch x 277 amino-acid alignment of panB; and 6408 branch x 274 amino-acid alignment of panC. Branches are shaded by taxonomy, and clades containing a majority of *panB/C* fusion sequences are highlighted in yellow. Key taxa related to the evolution of haptophyte *panB*, *panC* and *panB/C* genes across the eukaryotic tree of life are labelled.

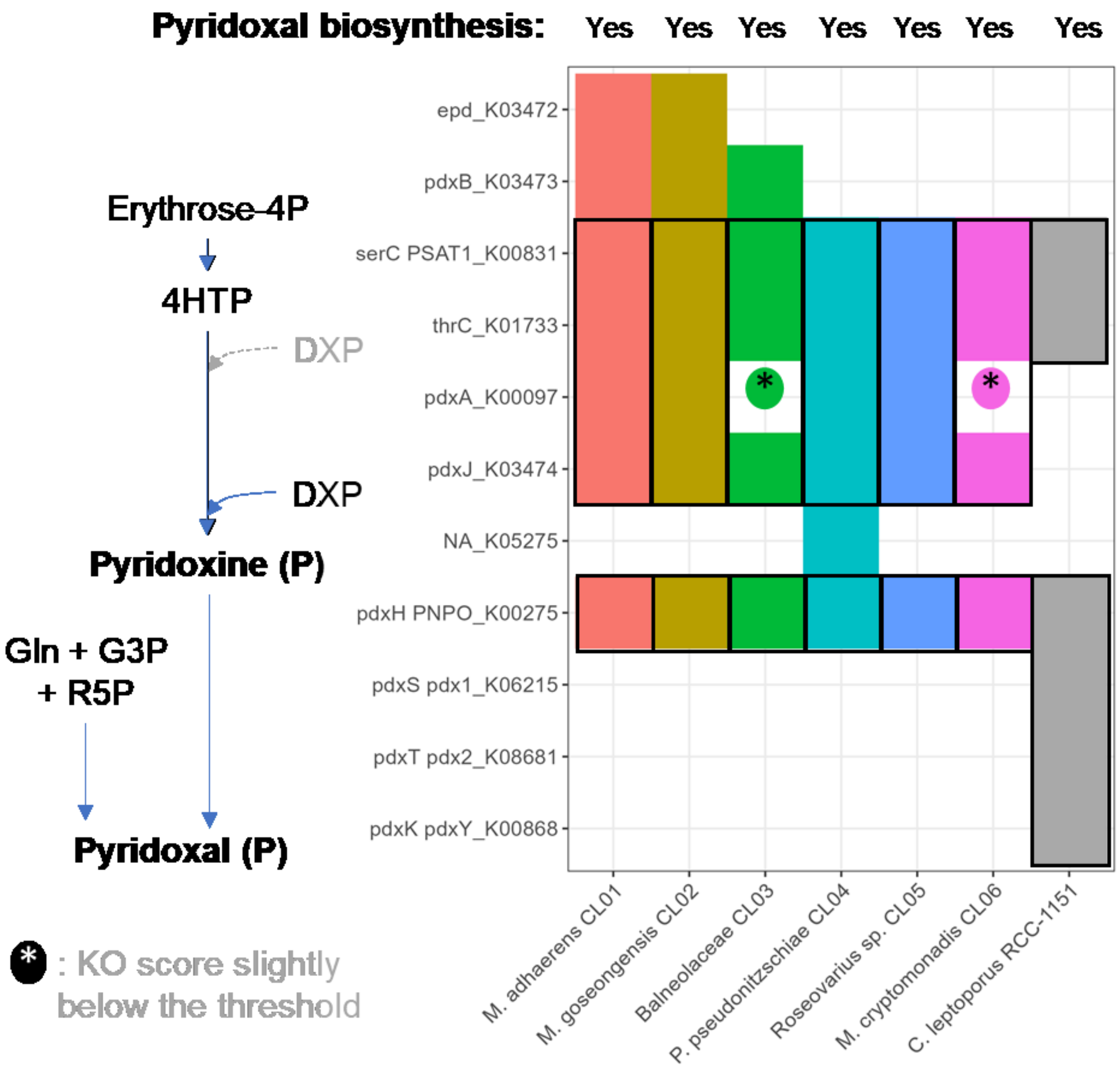

Figure S15.

Major steps of the pyridoxal (B6) biosynthesis.

Based on KEGGS map00770 and complementary literature (Parra et al. 2018, Richts and Commichau 2021, Kim et al. 2019), the main intermediates and reactions (left) together with related genes and/or KO number (right) are presented. Colored tiles indicate detected KOs in the genomes and complete pathways are outlined in black. In grey is an alternative synthesis route of pyridoxine synthesis. Blast searches and manual analyses of below-threshold KO scores allowed to retrieve missing genes indicated by "*". **Erythrose-4P**: D-Erythrose-4-phosphate; **4HTP**: 4-hydroxy-threonine-phosphate; **DXP**: Deoxyxylulose-5-phosphate; **Gln**: Glutamine, **G3P**: Glyceraldehyde-3-phosphate; **R5P**: D-ribose-5-phosphate. (P) denotes the phosphorylated forms of pyridoxine/pyridoxal.

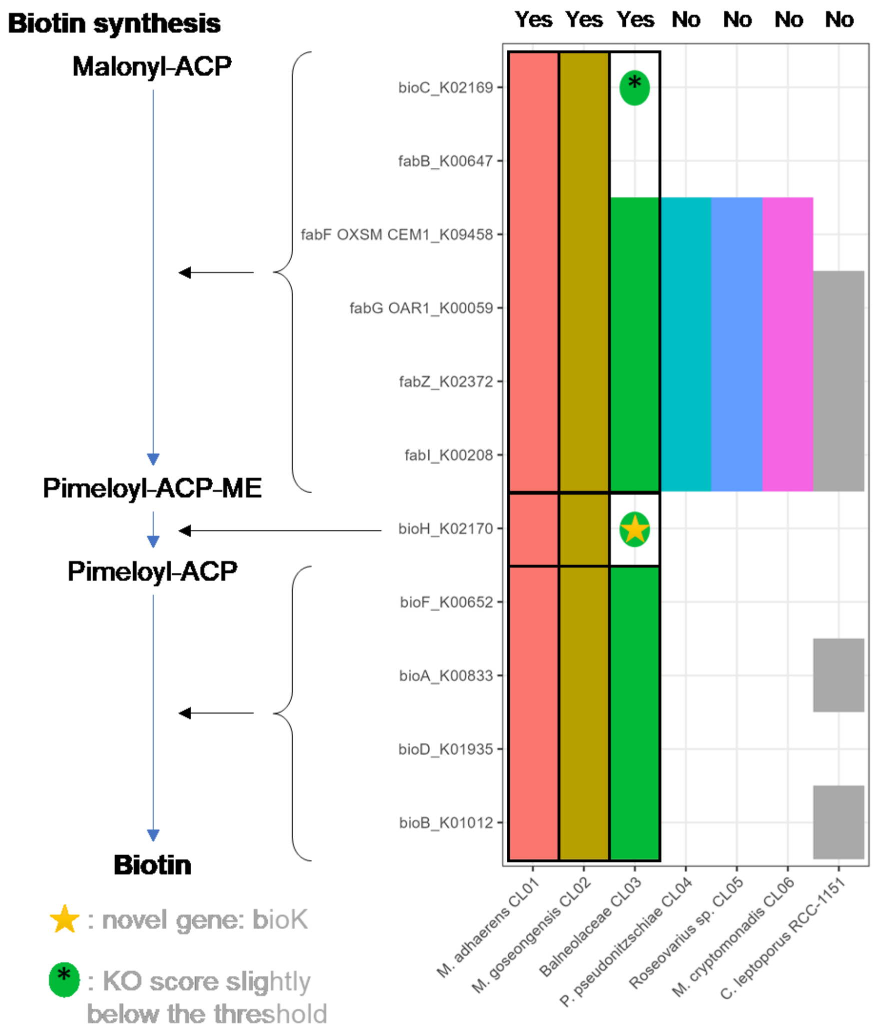

Figure S16.

Major steps of the biotin (B7) biosynthesis.

Based on KEGGS map00770 and complementary literature (Shapiro et al. 2012, Sirithanakorn & Cronan 2021), the main intermediates and reactions (left) together with related genes and/or KO number (right) are presented. Colored tiles indicate detected KOs in the genomes and complete pathways are outlined in black. Blast searches and manual analyses of below-threshold KO scores allowed to retrieve missing genes indicated by "*" and a star. The latter points to a putative esterase related to *bioK* (Supplemental Figure S15), an alternative to bioH. **ACP**: acyl-carrier protein; **Pimeloyl-ACP-ME**: Pimeloyl-acp methyl ester.

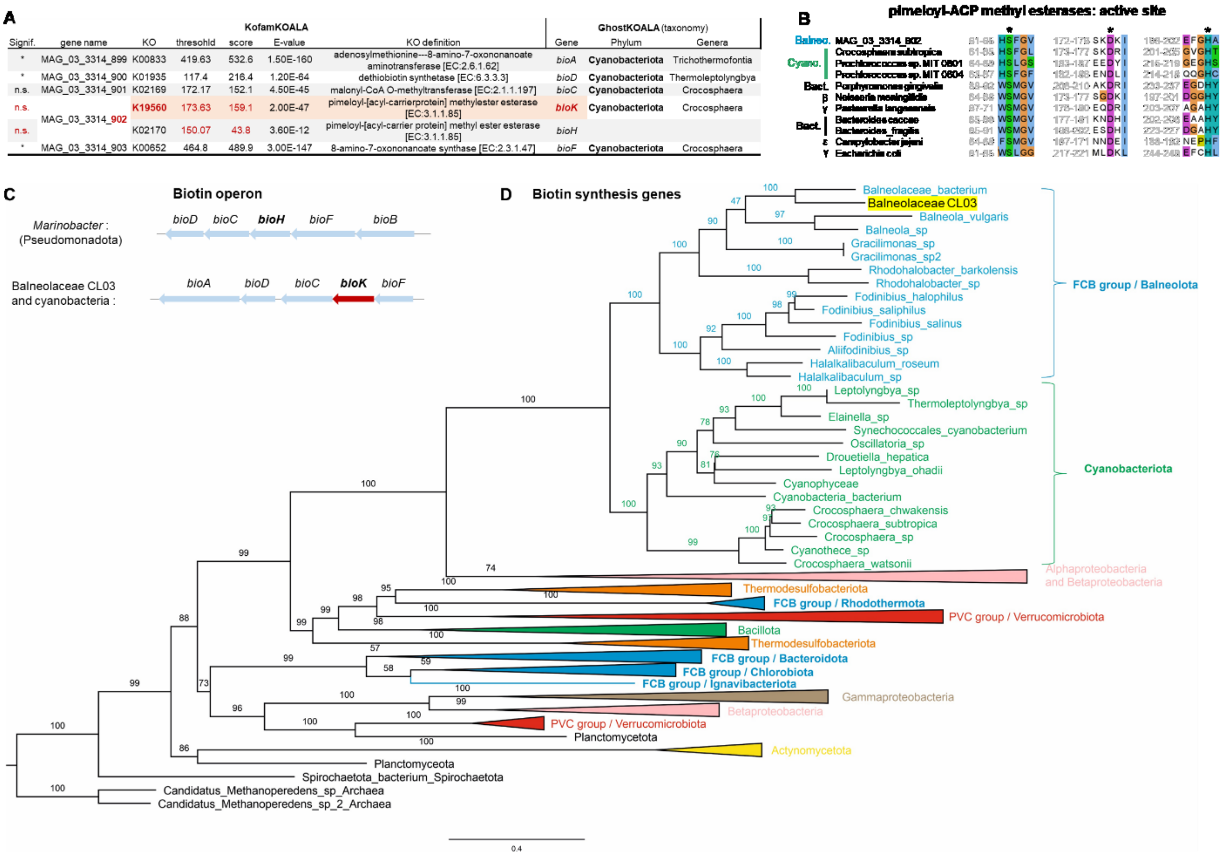

Figure S17.

Identification of a novel member of the bioK family in Balneolota bacteria

A. KO and taxonomy assignment by KofamKOALA and GhostKOALA for the gene models located between *bioA* and *bioF* in Balneolaceae strain CL03. Note that genes are named after their order in the genome assembly, suggesting that they form an operon. Symbol "*" and n.s. indicate significant and not significant, respectively, according to default threshold. Previous work on biotin operon in Balneolota did not detect *bioK* (Galisteo et al. 2023)
B. Pimeloyl-acyl-carrier-protein esterase enzymes from representative bacteria (Balneo: balneolota; Cyano: cyanobacteria; Bact: bacteroidota; β/γ/ε: beta-/gamma-/epsilon-proteobacteria) were aligned using MAFFT (version 7, --reorder --maxiterate 0 --globalpair input; Katoh et al. 2019) and visualized using JalView v2 (Waterhouse et al, 2009). Shown are the active site of bioH bioG bioK enzymes (Shapiro et al. 2012), with "*" indicating the crucial conserved residues.
C. Organization of the biotin synthesis genes (top) in *Marinobacter* strains CL01 and CL02 (conserved among Pseudomonadales) and (bottom) in Balneolaceae strain CL03 (conserved in Balneolota and Cyanobacteria), highlighting the conserved position of the pimeloyl-ACP-esterase genes *bioH* and *bioK* upstream of *bioC-bioD*. Note that *bioB* is located on distant genomic locations in Balneolota and Cyanobacteria genomes.
D. Maximum-likelihood tree inferred from the concatenated matrix of five proteins *bioF-bioK-bioC-biD-bioA* from Balneolaceace CL03 and representative sequences from NCBI. Branch support is expressed as ultrafast bootstrap (UFBoot) values (well-supported at a threshold of 95% (Minh et al., 2013)). While Balneolota is considered a sister phylum with Rhodothermota, Bacteroidota and Chlorobiota (García-López et al. 2019), proteins of the biotin operon display higher similarity between Balneolota and

the taxonomically distant phylum Cyanobacteria, pointing to an ancient horizontal transfer of the whole operon.

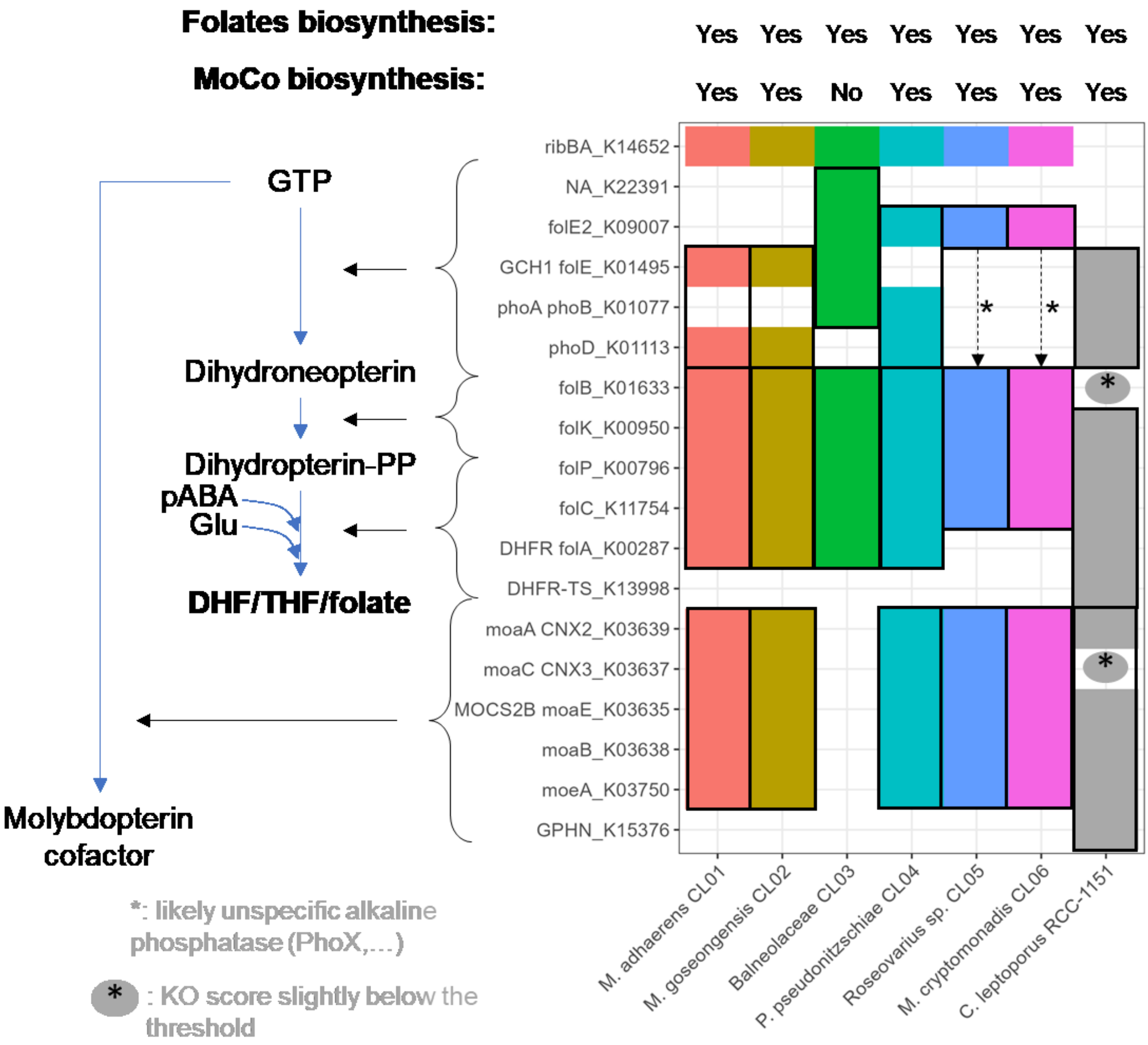

Figure S18.

Major steps of the folate (B9) and molybdopterin cofactor biosynthesis.

Based on KEGGS map00790 and complementary literature (Crécy-Lagard et al. 2007), the main intermediates and reactions (left) together with related genes and/or KO number (right) are presented. Colored tiles indicate detected KOs in the genomes and complete pathways are outlined in black. Blast searches and manual analyses of below-threshold KO scores allowed to retrieve missing genes indicated by "*". In *C. leptoporus* the 7,8-dihydroneopterin aldolase/epimerase/oxygenase (folB) is putatively encoded by anno1.g31798.t1. The dotted arrows point to putative alternative phosphatase (Sebastian & Ammerman 2009). **GTP**: Guanosine 5'-triphosphate; **PP**: pyrosphosphate; **pABA**: 4-Aminobenzoate; **Glu**: glutamate, **DHF**: dihydrofolate; **THF**: tetrahydrofolate.

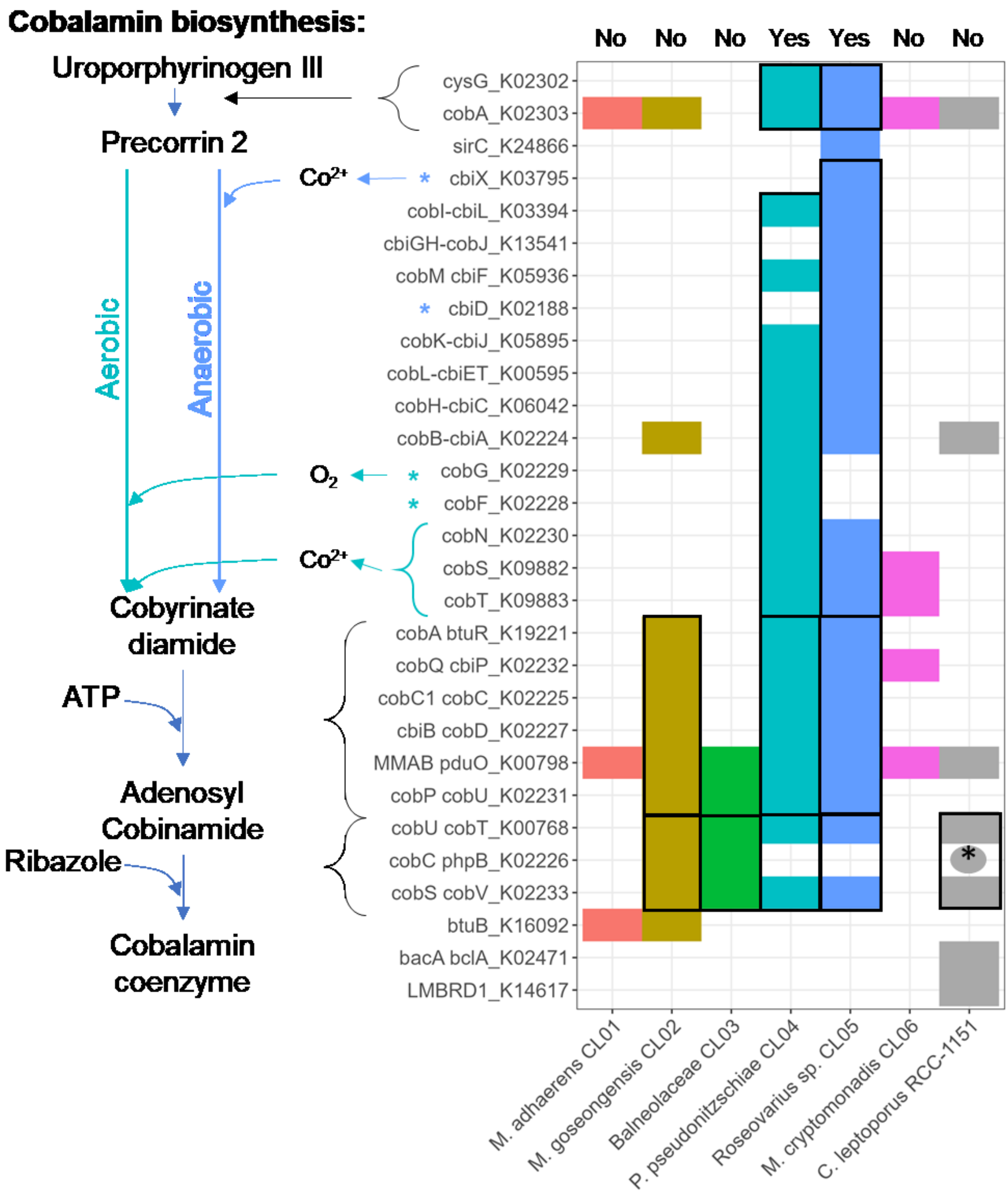

Figure S19.

Major steps of the cobalamin (B12) biosynthesis.

Based on KEGGS map00860 and complementary literature (Helliwell 2017), the main intermediates and reactions (left) together with related genes and/or KO number (right) are presented. Colored tiles indicate detected KOs in the genomes and complete pathways are outlined in black. Light or dark blue arrows highlight the aerobic pathway *P. pseudonitzschiae* CL04 and anaerobic pathway in *Roseovarius sp.* CL05, respectively. Similarly, symbols "*" indicate the genes specific to the aerobic or anaerobic pathways. **Uro..gen III**: Uroporphyrinogen III; **Co^{2+}** : cobalt (II); **O_2**, dioxygen; **ATP**: Adenosine-tri-phosphate.

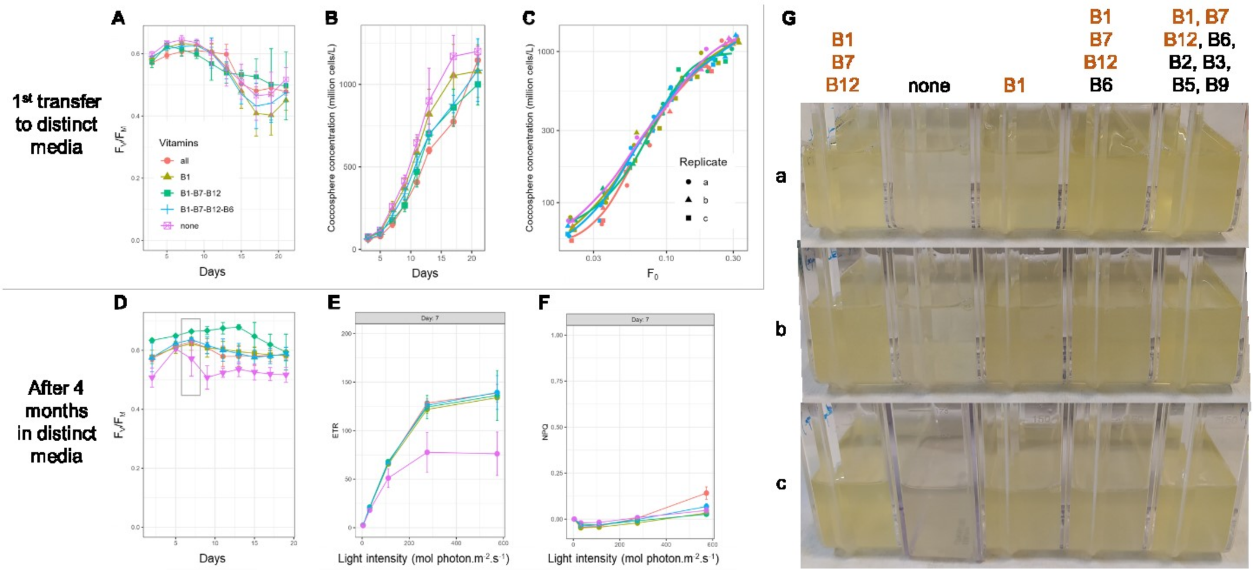

Figure S20.

Growth and photosynthetic performance of C. leptoporus under various vitamin regimes.

Cells were pre-cultured in K/2 medium then transferred to distinct media which were renewed every three weeks in each replicate.

A-C. Upon this first transfer, photosystem II maximum yield (F_V/F_M, panel A) and cell number (B) were monitored. The minimal fluorescence (Figure 5A) correlated well with cell concentration (C).

D-G. After four months, cells were monitored again (Figure 5B) and displayed little difference in PSII maximum yield (D). During the exponential phase (day 7), the relative electron transfer rate (ETR, panel E) and non-photochemical quenching (NPQ, panel F) were recorded after 1 min at various light intensities. Growth delay in absence of vitamin supplementation was clearly visible (G).

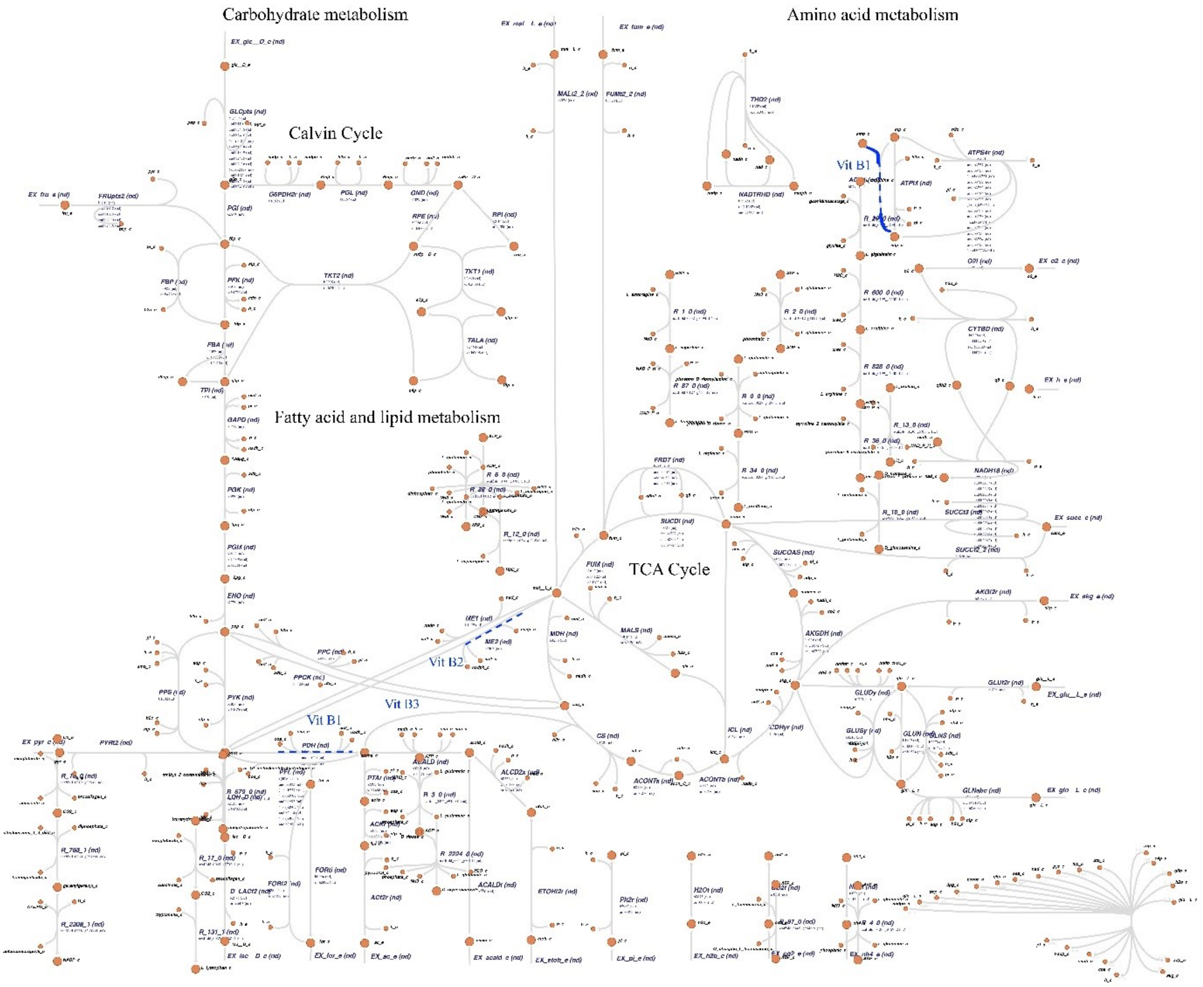

Figure S21.

Metabolic reconstruction of *Calcidiscus*

A metabolic reconstruction was generated by parsing the genome annotations of *Calcidiscus* through the KEGG and IUBMB databases (Kanehisa et al., 2021, UBMB, 2022). The draft network was assembled into a systems biology model (SBML Level 3, XML format) using LibSBML, COPASI, and COBRApy (Bornstein et al., 2008, Hoops et al., 2006, Ebrahim et al., 2013). The metabolic reconstruction consisted of 2,102 reactions, 478 metabolites, 460 genes, and 129 annotated pathways. Additional manual refinements were performed using COPASI and Metanext, leading to further improvements in consistency and annotation (Hoops et al., 2006, Moretti et al., 2021) and to enable interoperability with multiple databases such as KEGG, METACYC and BIGGS (Kanehisa et al., 2021, Caspir et al., 2014, Norsigian et al., 2020).
The metabolic map was created with the Escher tool and the simulated SBML metabolic model *(*Zachary et al., 2015*)* and is available in the Biomodels, and PatMeDB repositories (Malik-Sheriff et al., 2020, Bannerman et al., 2023).

Table S1.
Sequencing statistics.

Set	Sequences	Total length (Gb)	Mean length (kb)	Quartile length (kb)			N50	Quality (%)	
				Q1	Q2	Q3		Q20	Q30
Pass	1,687,664	10.3	6.1	1.225	3.121	8.052	13.136	86.37	75.6
Fail	584,798	3.10	5.3	0.883	2.673	6.532	11.802	5.98	0.88

Table S2.

Repeat detection outputs using RepeatMasker on *Calcidiscus* genome (MAG_1000).

```
==================================================
file name: MAG_1000.fa
sequences:          1441
total length:  452936211 bp  (452936211 bp excl N/X-runs)
GC level:         59.12 %
bases masked:   51893926 bp ( 11.46 %)
==================================================
               number of      length   percentage
               elements*    occupied  of sequence
--------------------------------------------------
Retroelements            0            0 bp    0.00 %
   SINEs:                0            0 bp    0.00 %
   Penelope              0            0 bp    0.00 %
   LINEs:                0            0 bp    0.00 %
    CRE/SLACS            0            0 bp    0.00 %
     L2/CR1/Rex          0            0 bp    0.00 %
     R1/LOA/Jockey       0            0 bp    0.00 %
     R2/R4/NeSL          0            0 bp    0.00 %
     RTE/Bov-B           0            0 bp    0.00 %
     L1/CIN4             0            0 bp    0.00 %
   LTR elements:         0            0 bp    0.00 %
     BEL/Pao             0            0 bp    0.00 %
     Ty1/Copia           0            0 bp    0.00 %
     Gypsy/DIRS1         0            0 bp    0.00 %
       Retroviral        0            0 bp    0.00 %

DNA transposons          0            0 bp    0.00 %
   hobo-Activator        0            0 bp    0.00 %
   Tc1-IS630-Pogo        0            0 bp    0.00 %
   En-Spm                0            0 bp    0.00 %
   MuDR-IS905            0            0 bp    0.00 %
   PiggyBac              0            0 bp    0.00 %
   Tourist/Harbinger     0            0 bp    0.00 %
   Other (Mirage,        0            0 bp    0.00 %
    P-element, Transib)

Rolling-circles          0            0 bp    0.00 %

Unclassified:            0            0 bp    0.00 %

Total interspersed repeats:           0 bp    0.00 %


Small RNA:               0            0 bp    0.00 %

Satellites:              1          514 bp    0.00 %
Simple repeats:     433168     47762975 bp   10.55 %
Low complexity:      33756      4130437 bp    0.91 %
==================================================

* most repeats fragmented by insertions or deletions
  have been counted as one element


The query species was assumed to be haptista
RepeatMasker version 4.1.2-p1 , sensitive mode

run with rmblastn version 2.10.0+
FamDB: CONS-Dfam_3.3
```

Table S3.

Statistics of mapping RNA-seq onto *Calcidiscus* genome (MAG_1000)

Number of input reads	22202199
Average input read length	100
UNIQUE READS:	
Uniquely mapped reads number	18128812
Uniquely mapped reads %	81.65%
Average mapped length	98.62
Number of splices: Total	3261052
Number of splices: Annotated (sjdb)	0
Number of splices: GT/AG	2444000
Number of splices: GC/AG	739176
Number of splices: AT/AC	283
Number of splices: Non-canonical	77593
Mismatch rate per base, %	0.54%
Deletion rate per base	0.02%
Deletion average length	2.05
Insertion rate per base	0.01%
Insertion average length	1.71
MULTI-MAPPING READS:	
Number of reads mapped to multiple loci	2159197
% of reads mapped to multiple loci	9.73%
Number of reads mapped to too many loci	232069
% of reads mapped to too many loci	1.05%
UNMAPPED READS:	
Number of reads unmapped: too many mismatches	0
% of reads unmapped: too many mismatches	0.00%
Number of reads unmapped: too short	1615428
% of reads unmapped: too short	7.28%
Number of reads unmapped: other	66693
% of reads unmapped: other	0.30%
CHIMERIC READS:	
Number of chimeric reads	0
% of chimeric reads	0.00%

Table S4.
Gene detection in *Calcidiscus* genome (MAG_1000) statistics using braker3.

Feature	Source	Count	Length (bp)		
			Median	Mean	SD
Gene	AUGUSTUS	26032	3047	4255	3812
	GeneMark.hmm3	1616	3191	4666	4289
	gmst	1771	738	1185	1583
	**Total**	**29419**			
Exon	AUGUSTUS	170336	123	278	530
	GeneMark.hmm3	29944	111	256	463
	gmst	4399	240	369	471
	**Total**	**204679**			
Intron	AUGUSTUS	142868	316	512	614
	GeneMark.hmm3	25418	293	550	778
	gmst	2212	244.5	480	1023
	**Total**	**170498**			

Table S5.

Putative horizontal gene transfers in *Calcidiscus* genome (MAG_1000).

Supplemental Dataset	GeneID	Support	Lineage	Subgroup	BLASTp nr	Localization	KEGG	PFAM	
SD1	g8749.t1	100	Amorphea	Animals	Retrotransposon-like protein 1 [Dissostichus eleginoides]	Other	K03281	N/A	N/A
SD2	g4189.t1	80	Bacteria	Alpha-proteobacteria	D-amino-acid transaminase [Thermohalobaculum xanthum]	Mitochondrion	K00824	PF01063	Amino-transferase class IV
SD3	g4189.t2	92	Bacteria	Alpha-proteobacteria	D-amino-acid transaminase [Thermohalobaculum xanthum]	Other	K00824	PF01063	Amino-transferase class IV
SD4	g5418.t1	67	Bacteria	Planctomycetes	esterase family protein [Verrucomicrobiales bacterium]	Mitochondrion	N/A	PF00756	Putative esterase
SD5	g2706.t1	63	Alveolates	Gonyaulacales	ucpB [Symbiodinium pilosum]	Plastid	N/A	PF00153	Mitochondrial carrier protein
SD6	g6478.t1	53	Alveolates	Gonyaulacales	RDH14 [Symbiodinium microadriaticum]	Other	N/A	N/A	N/A
SD7	g6477.t1	69	Alveolates	Gonyaulacales	RDH14 [Symbiodinium necroappetens]	Other	N/A	PF00106	short chain dehydrogenase
SD8	g3969.t1	96	Alveolates	Peridiniales	bath-38 [Symbiodinium sp. CCMP2592]	Other	N/A	PF00651	BTB/POZ domain
SD9	g10292.t1	59	Alveolates	Syndiniales	L-xylulose reductase [Pogona vitticeps]	Other	K03331	PF13561	Enoyl-(Acyl carrier protein) reductase
SD10	g2453.t1	100	Alveolates	Gonyaulacales	GIP [Symbiodinium sp. KB8]	Other	N/A	PF01323	DSBA-like thioredoxin domain
SD11	g7423.t1	100	Alveolates	Gonyaulacales	FAD-dependent oxidoreductase [Chloroflexota bacterium]	Other	N/A	PF07992	Pyridine nucleotide-disulphide oxidoreductase
SD12	g6416.t1	100	Alveolates	Gonyaulacales	serine carboxypeptidase-like family S10 [Achlya hypogyna]	Plastid	K09645	N/A	N/A
SD13	g4716.t1	100	Alveolates	Gonyaulacales	glutathione s-transferase-like protein [Chrysochromulina tobinii]	Other	N/A	N/A	N/A
SD14	g8853.t1	100	Alveolates	Peridiniales	tri1 [Symbiodinium pilosum]	Other	N/A	PF03747	ADP-ribosylglycohydrolase
SD15	g9687.t1	100	Alveolates	Gymnodiniales	SDR family NAD(P)-dependent oxidoreductase [Magnetovibrio sp.]	Other	N/A	PF00106	short chain dehydrogenase
SD16	g2480.t1	99	Stram.	Bolidophytes	TPA: enoyl-CoA hydratase/isomerase family protein [Myxococcales bacterium]	Mitochondrion	K13239	PF00378	Enoyl-CoA hydratase/isomerase
SD17	g2480.t2	99	Stram.	Bolidophytes	TPA: enoyl-CoA hydratase/isomerase family protein [Myxococcales bacterium]	Mitochondrion	K13239	PF00378	Enoyl-CoA hydratase/isomerase
SD18	g2560.t1	80	Stram.	Diatoms/ Pinguiophytes	hypothetical protein HDU76_012710 [Blyttiomyces sp. JEL0837]	Endomembrane/ Secreted	N/A	N/A	N/A
SD19	g5987.t1	96	Stram.	Pelagophytes	hypothetical protein M885DRAFT_563868 [Pelagophyceae sp. CCMP2097]	Other	N/A	N/A	N/A
SD20	g9274.t1	100	Stram.	Diatoms	FKBP_C-domain-containing protein [Fragilariopsis cylindrus CCMP1102]	Endomembrane/ Secreted	K09577	PF00254	FKBP-type peptidyl-prolyl cis-trans isomerase
SD21	g10036.t1	97	Stram.	Diatoms/ Pelagophytes	aspartyl protease [Nitzschia inconspicua]	Mitochondrion	N/A	PF13650	Aspartyl protease
SD22	g10036.t2	99	Stram.	Diatoms/ Pelagophytes	aspartyl protease [Nitzschia inconspicua]	Endomembrane	N/A	PF13650	Aspartyl protease
SD23	g10564.t1	94	Stram.	Pelagophytes	Glucoside xylosyltransferase 2 [Picochlorum sp. SENEW3]	Other	N/A	PF13578	Methyltransferase domain

Table S6.

CheckM statistics for bacterial MAGs.

MAG	Checkm markers					Marker count						Completeness	Contamination	Strain	GC %	Length	Contig	Classification
	Marker lineage	UID	# genomes	# markers	# marker sets	0	1	2	3	4	5+	%	%	heterogeneity %		(Mb)	count	in GTDBtk
MAG_01	k__Bacteria	(UID203)	5449	104	58	1	103	0	0	0	0	98.28	0	0	57.2	4.44	1	*Marinobacter adhaerens*
MAG_02	k__Bacteria	(UID203)	5449	104	58	0	104	0	0	0	0	100	0	0	59.2	4.14	1	*Marinobacter sp017643965*
MAG_03	k__Bacteria	(UID203)	5449	104	58	0	104	0	0	0	0	100	0	0	39.1	3.80	1	Family: Balneolaceae; Genus: JAUICM01
MAG_04	k__Bacteria	(UID203)	5449	104	58	1	103	0	0	0	0	98.28	0	0	59.8	3.91	2	*Pseudosulfitobacter pseudonitzschiae_A*
MAG_05	k__Bacteria	(UID203)	5449	104	58	3	101	0	0	0	0	94.83	0	0	60.8	3.61	2	*Roseovarius sp913054395*
MAG_06	k__Bacteria	(UID203)	5449	104	58	14	90	0	0	0	0	87.15	0	0	57.7	4.02	3	*Marivita sp947503115*
MAG_228	k__Bacteria	(UID203)	5449	103	57	31	71	1	0	0	0	75.12	0.16	0	61.3	2.82	21	Order: Phycisphaerales; Family: UBA1924
MAG_103	k__Bacteria	(UID203)	5449	104	58	18	84	2	0	0	0	74.14	1.88	50	42.6	3.52	40	*Pleionea mediterranea*
MAG_66	k__Bacteria	(UID203)	5449	104	58	45	59	0	0	0	0	46.87	0	0	64.9	2.83	26	*Mameliella sp019392335*
MAG_369	k__Bacteria	(UID203)	5449	104	58	77	27	0	0	0	0	39.66	0	0	45.2	1.69	26	*Lentilitoribacter sp020628775*
MAG_169	k__Bacteria	(UID203)	5449	104	58	51	52	1	0	0	0	33.41	0.16	0	60.9	1.08	20	Genus: Nisaea
MAG_105	k__Bacteria	(UID203)	5449	104	58	87	17	0	0	0	0	26.72	0	0	62.0	1.80	26	Genus: Roseovarius
MAG_546	k__Bacteria	(UID203)	5449	104	58	102	2	0	0	0	0	3.45	0	0	63.4	0.23	7	Too small
MAG_01	c__Gammaproteobacteria	(UID4444)	263	507	232	4	502	1	0	0	0	99.1	0.09	0	57.2	4.44	1	*Marinobacter adhaerens*
MAG_02	c__Gammaproteobacteria	(UID4444)	263	507	232	1	505	1	0	0	0	99.95	0.43	0	59.2	4.14	1	*Marinobacter sp017643965*
MAG_103	c__Gammaproteobacteria	(UID4442)	899	312	185	74	234	4	0	0	0	71.75	1.24	50	42.6	3.52	40	*Pleionea mediterranea*
MAG_04	f__Rhodobacteraceae	(UID3360)	56	582	313	2	578	2	0	0	0	99.36	0.27	0	59.8	3.91	2	*Pseudosulfitobacter pseudonitzschiae_A*
MAG_05	f__Rhodobacteraceae	(UID3361)	46	654	332	8	644	2	0	0	0	98.24	0.03	0	60.8	3.61	2	*Roseovarius sp913054395*
MAG_06	f__Rhodobacteraceae	(UID3361)	46	654	332	45	607	2	0	0	0	93.63	0.38	0	57.7	4.02	3	*Marivita sp947503115*
MAG_66	f__Rhodobacteraceae	(UID3361)	46	654	332	353	298	3	0	0	0	44.71	0.5	0	64.9	2.83	26	*Marivita sp947503115*
MAG_228	k__Bacteria	(UID2565)	2921	143	88	54	88	1	0	0	0	62.33	0.07	0	61.3	2.82	21	Order: Phycisphaerales; Family: UBA1924
MAG_169	c__Alphaproteobacteria	(UID3305)	564	349	230	237	111	1	0	0	0	23.44	0.04	0	60.9	1.08	20	Genus: Nisaea

Supplementary Dataset S1. (separate file)

Phylogenetic distribution and curation of panB and panC homologues.

This workbook contains the sequence-level data used to identify panB and panC homologues, including fused panB/panC proteins, across UniProt, JGI PhycoCosm, MMETSP, and OneKP-derived sequence libraries. Candidate homologues were identified by reciprocal BLAST best-hit searches using the *Calcidiscus leptoporus* panBC sequence as reference, screened for PanBC-associated Pfam domains with HMMER/hmmsearch, aligned, manually curated, and evaluated by phylogenetic analysis. The workbook includes taxonomic summaries, species-level distributions, candidate sequences and annotations, Pfam-domain results, inclusion/exclusion rationale, curated alignments, and tree topologies. The "Totals" sheet summarizes inferred homologue distributions across algal groups; "Distributions by species" lists verified homologues by taxon; "Sequences" provides sequence identifiers, annotations, Pfam-domain scores, and curation status; "Alignments" provides curated alignments; and "Trees" provides the inferred topologies used to validate panB, panC, and fused panB/panC distributions.

Supplementary Dataset S2. (separate file)

Best-scoring RaxML trees of inferred *Calcidiscus* HGTs
(SD1-SD23).

Trees were generated from a combined set of the top 100 BLASTp hits to the predicted protein model coded by each gene from a reference library of Uniref, jgi genomes, MMETSP and OneKp transcriptomes, per Dorrell et al (2021), alongside the single best hits from 151 taxonomic sub-categories represented by these libraries, as defined in the same study. Trees were generated via two rounds of iterative alignment (mafft, MUSCLE, GeneIOUS) under default conditions, manual and automated (trimal) trimming, and RaxML phylogeny the using PROTGAMMAJTT substitution matrix and 350 bootstrap replicates. Branch thicknesses relate to bootstrap support of the best scoring topology, and bootstrap values for selected nodes that confirm the horizontal origin of each *Calcidiscus* gene are individually labelled. Branches are coloured by taxonomic origin: **magenta**, haptophytes; **purple**, cryptomonads; **orange**, stramenopiles; **dark yellow**, alveolates; **red**, red algae; **green**, green algae and plants: **grey**, other eukaryotes; **dark blue**, prokaryotes. *Calcidiscus* query sequences are labelled in **dark red**.

Supplementary Dataset S3. (separate file)

Whole proteome assessment of bacterial MAGs
(SD24-SD30).

Protein sequences were predicted for the six bacterial genomes assembled in this study. For reference genomes, protein sequences were downloaded from NCBI: *Balneola vulgaris*: DSM_17893; *Gracilimonas amylolytica*: GCF_002911695.1; *Marinobacter adhaerens* HP15: GCF_000166295.1; *Marinobacter goseongensis*: GCA_023156235.1; *Marinobacter lipolyticus* SM19: GCF_000397065.2; *Marivita cryptomonadis*: GCF_002115725.1; *Roseovarius lutimaris*: GCF_900115165.1; *Roseovarius marisflavi*: GCF_900142625.1; *Pseudosulfitobacter pseudonitzschiae*: GCF_000712315.1. Proteomes were compared (circle plots) and annotated with KEGGS Orthology numbers (KOs) using DRAM (https://github.com/WrightonLabCSU/DRAM) and upset diagrams generated using ggVennDiagram (https://github.com/gaospecial/ggVennDiagram).

In figures SD26 to SD30, shown are (A) circle plots of protein similarity and (B) upset diagrams between MAGs and closest relatives.
