# Supplementary material for: The Calcidiscus leptoporus genome reveals vitamin-mediated holobiont interactions
Source: ISME Commun. 2026 Aug 23;6(1):ycag222. doi: 10.1093/ismeco/ycag222 (PMC13518057; doi:10.1093/ismeco/ycag222)
Supplement: Supplementary file 1 [file supplementary_material_ycag222.zip › Suppl Dataset SD2 HGT.pdf]

Supplementary Dataset S2.
Best-scoring RaxML trees of inferred *Calcidiscus* HGTs.
SD1-SD23

Trees were generated from a combined set of the top 100 BLASTp hits to the predicted protein model coded by each gene from a reference library of Uniref, jgi genomes, MMETSP and OneKp transcriptomes, per **(Dorrell et al., 2021)**, alongside the single best hits from 151 taxonomic sub-categories represented by these libraries, as defined in the same study. Trees were generated via two rounds of iterative alignment (mafft, MUSCLE, GeneIOUS) under default conditions, manual and automated (trimal) trimming, and RaxML phylogeny the using PROTGAMMAJTT substitution matrix and 350 bootstrap replicates. Branch thicknesses relate to bootstrap support of the best scoring topology, and bootstrap values for selected nodes that confirm the horizontal origin of each *Calcidiscus* gene are individually labelled. Branches are coloured by taxonomic origin: **magenta**, haptophytes; **purple**, cryptomonads; **orange**, stramenopiles; **dark yellow**, alveolates; **red**, red algae; **green**, green algae and plants: **grey**, other eukaryotes; **dark blue**, prokaryotes. Calcidiscus query sequences are labelled in **dark red**.

Fig. SD1. Phylogeny of a *Calcidiscus* retrotransposon-like/ calcium chloride channel protein (g8749.t1) of apparent animal origin

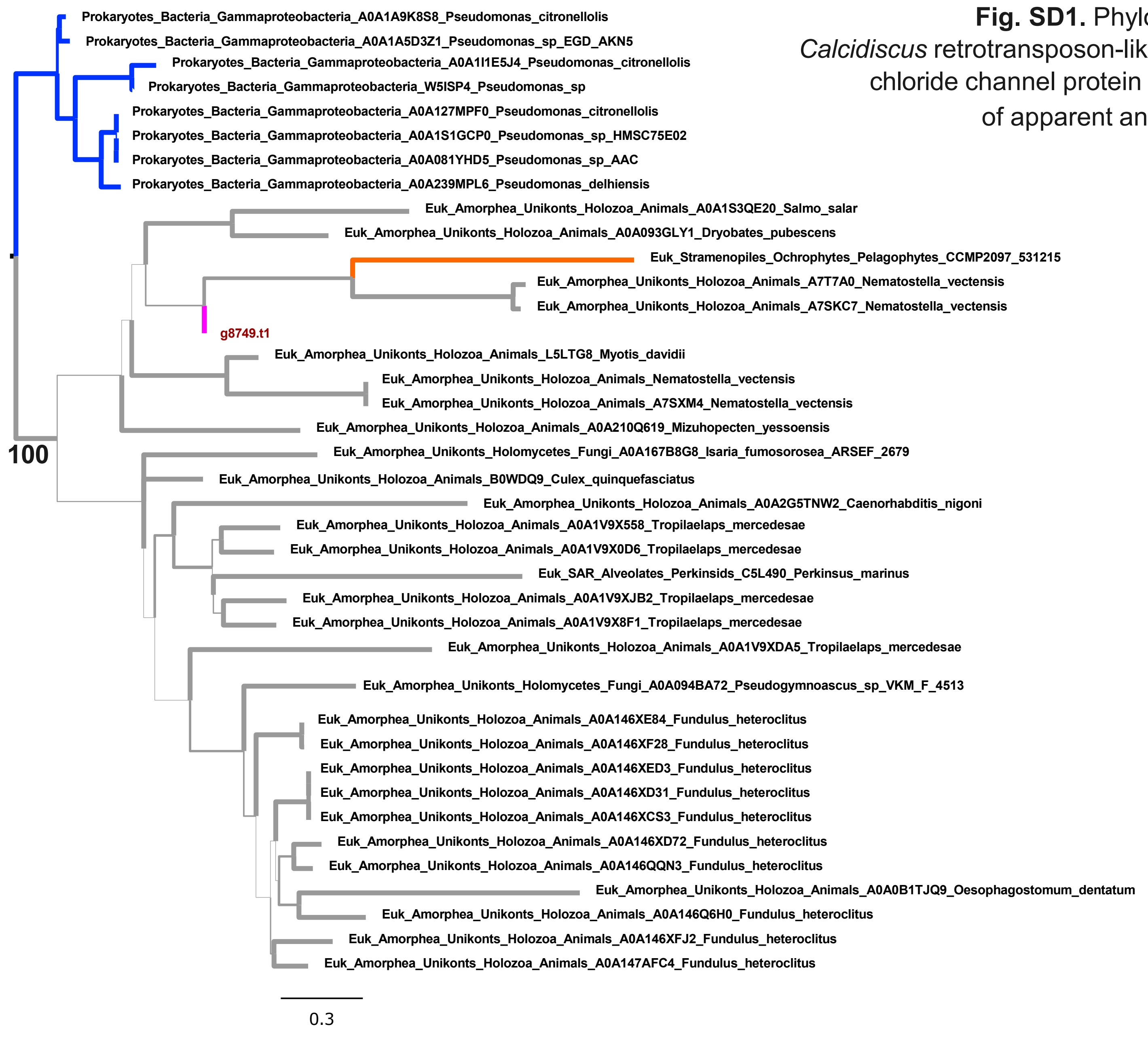

Fig. SD2. Phylogeny of a *Calcidiscus* putatively mitochondria-targeted D-amino acid aminotransferase (g4189.t1), of potential proteobacterial origin.

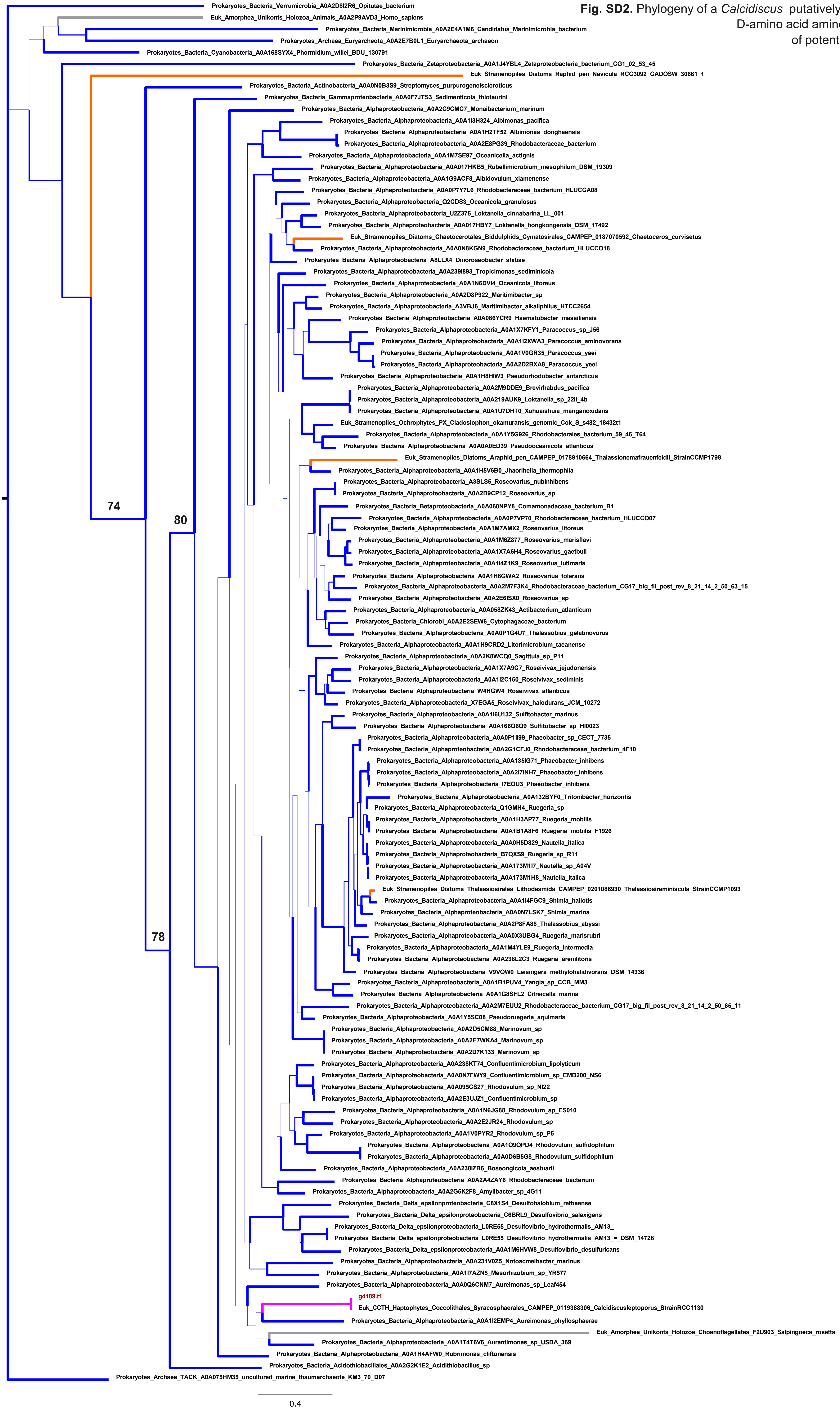

Fig. SD3. Phylogeny of a *Calcidiscus* D-amino acid transaminase (g4189.t2), of potential proteobacterial origin.

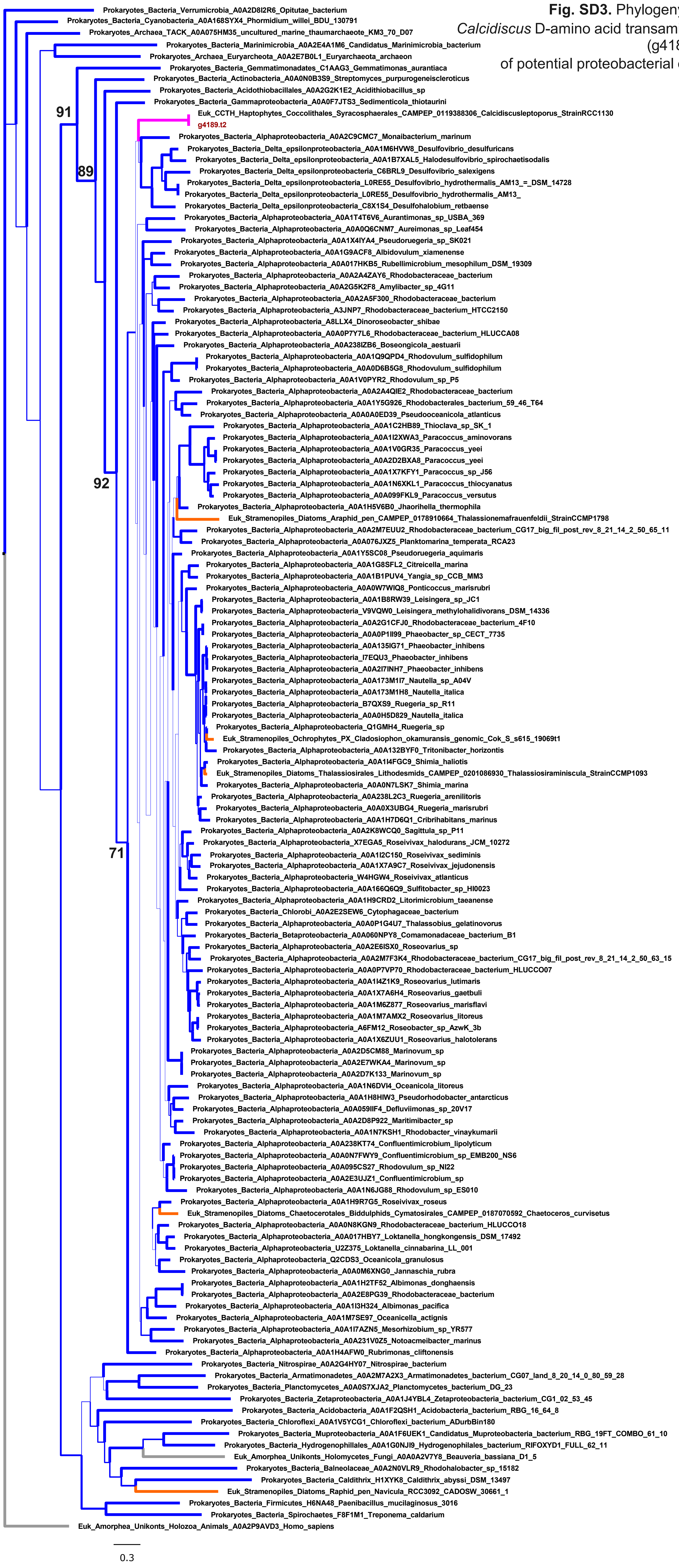

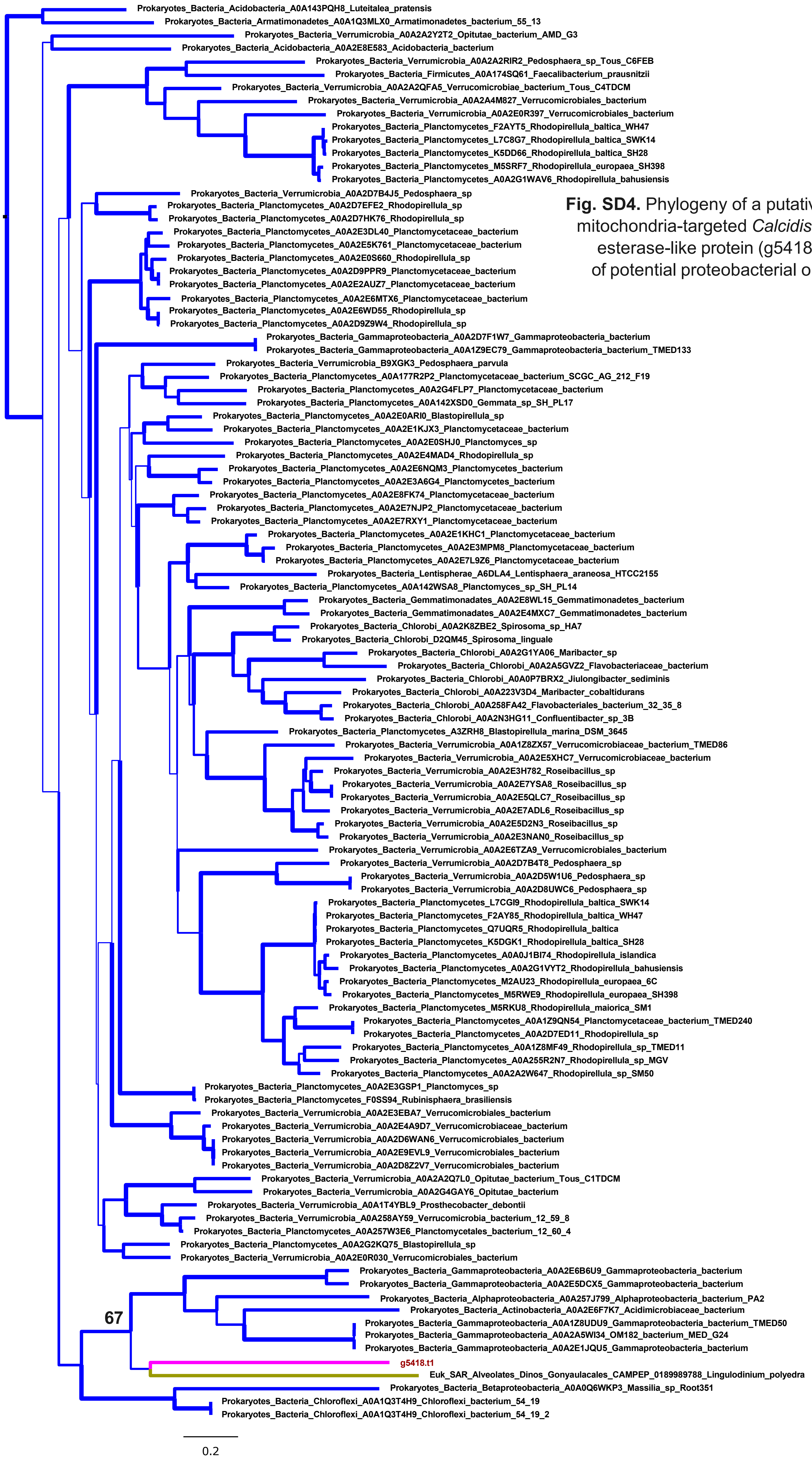

Fig. SD4. Phylogeny of a putatively mitochondria-targeted *Calcidiscus* esterase-like protein (g5418.t1), of potential proteobacterial origin

Fig. SD5. Phylogeny of a putatively plastid or plasma membrane-targeted *Calcidiscus* ucpB/ carrier family protein (g2706.t1), of potential dinoflagellate origin

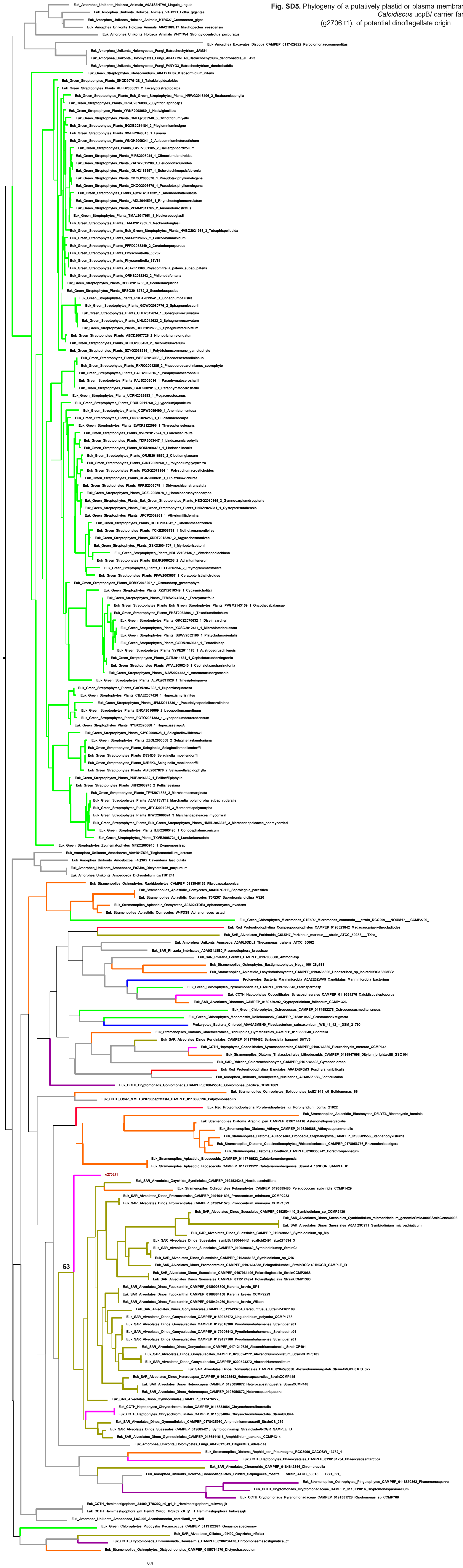

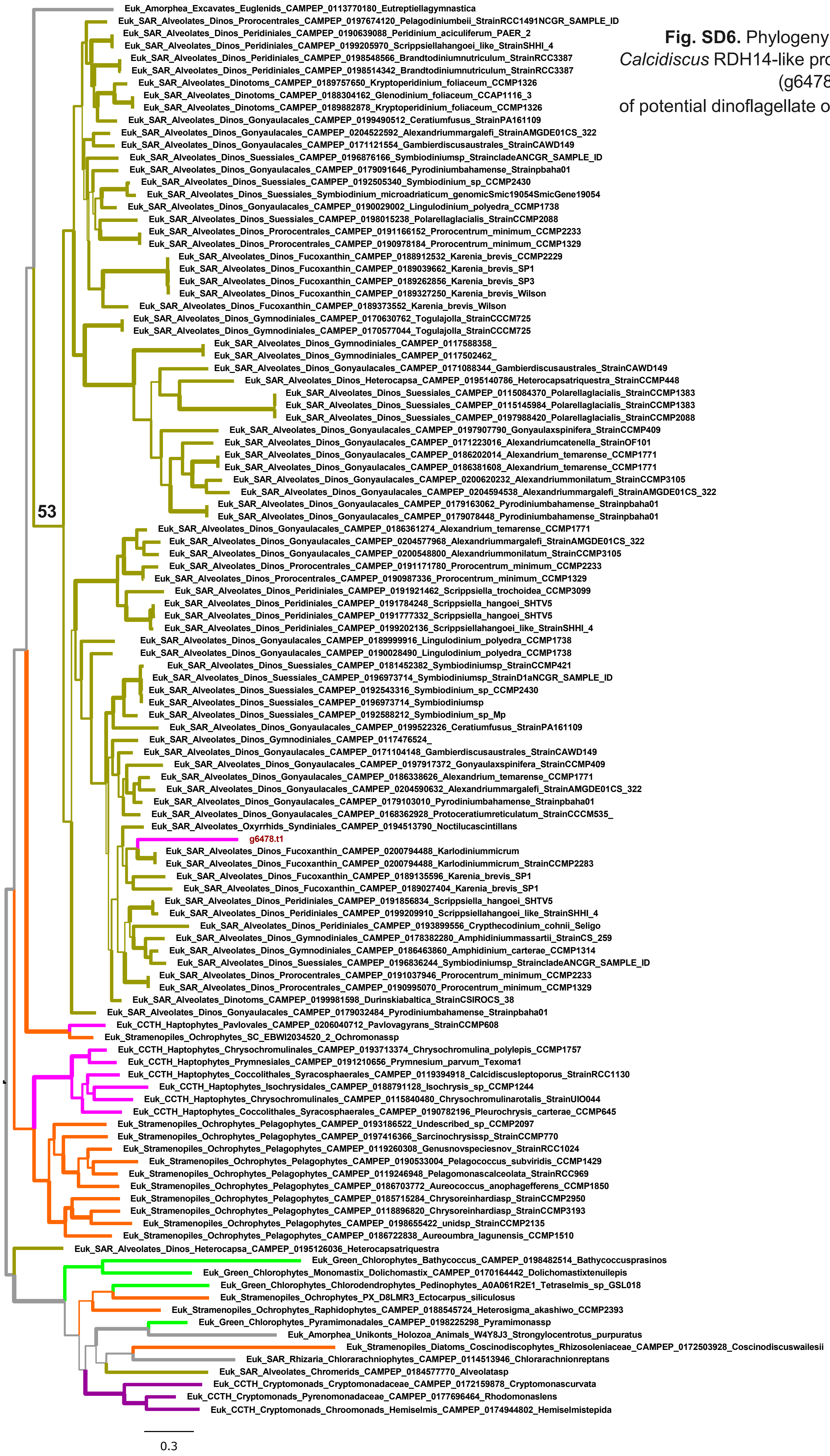

Fig. SD6. Phylogeny of a *Calcidiscus* RDH14-like protein (g6478.t1), of potential dinoflagellate origin.

Fig. SD7. Phylogeny of a *Calcidiscus* RDH14/ short-chain dehydrogenase protein (g6477.t1), of potential dinoflagellate origin.

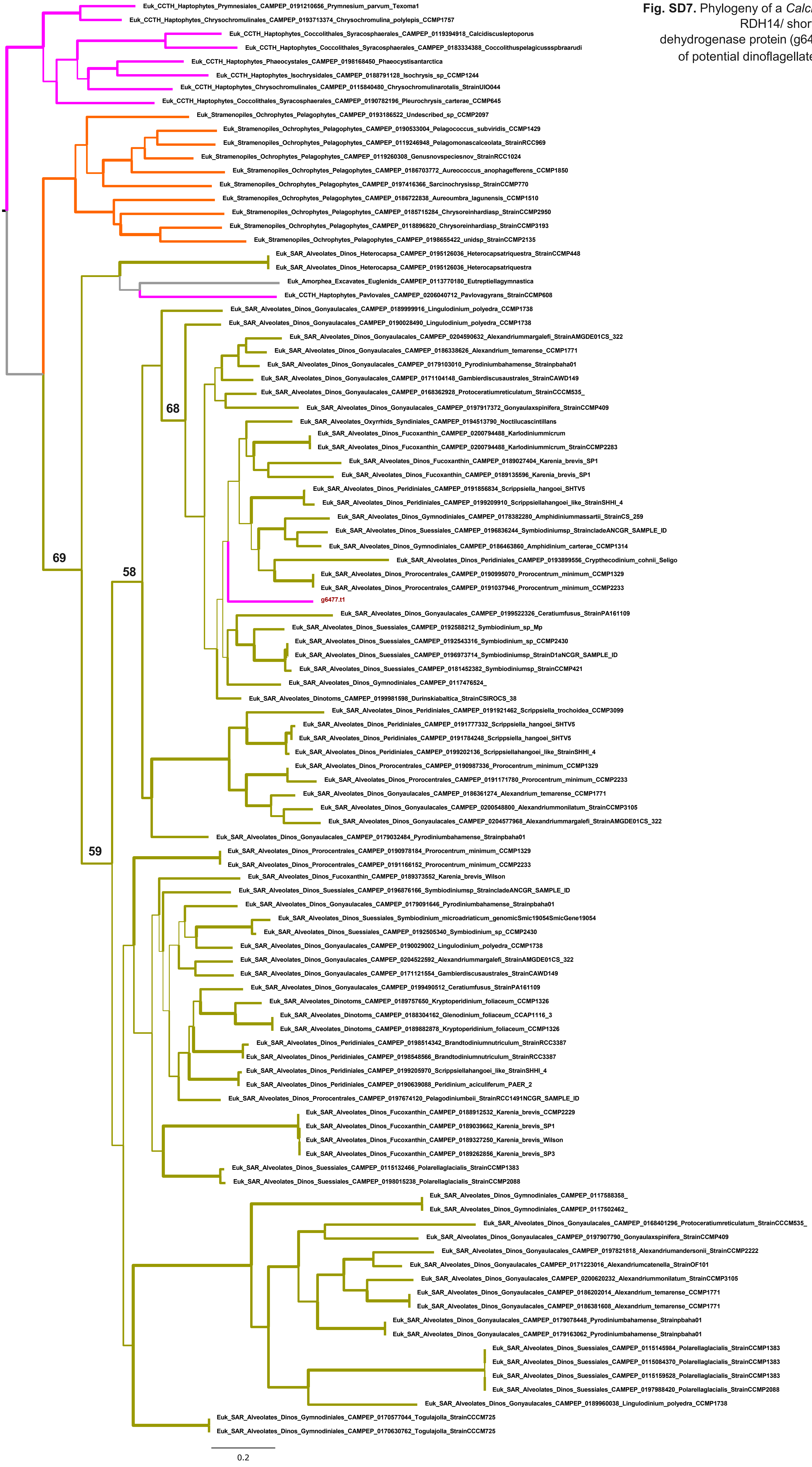

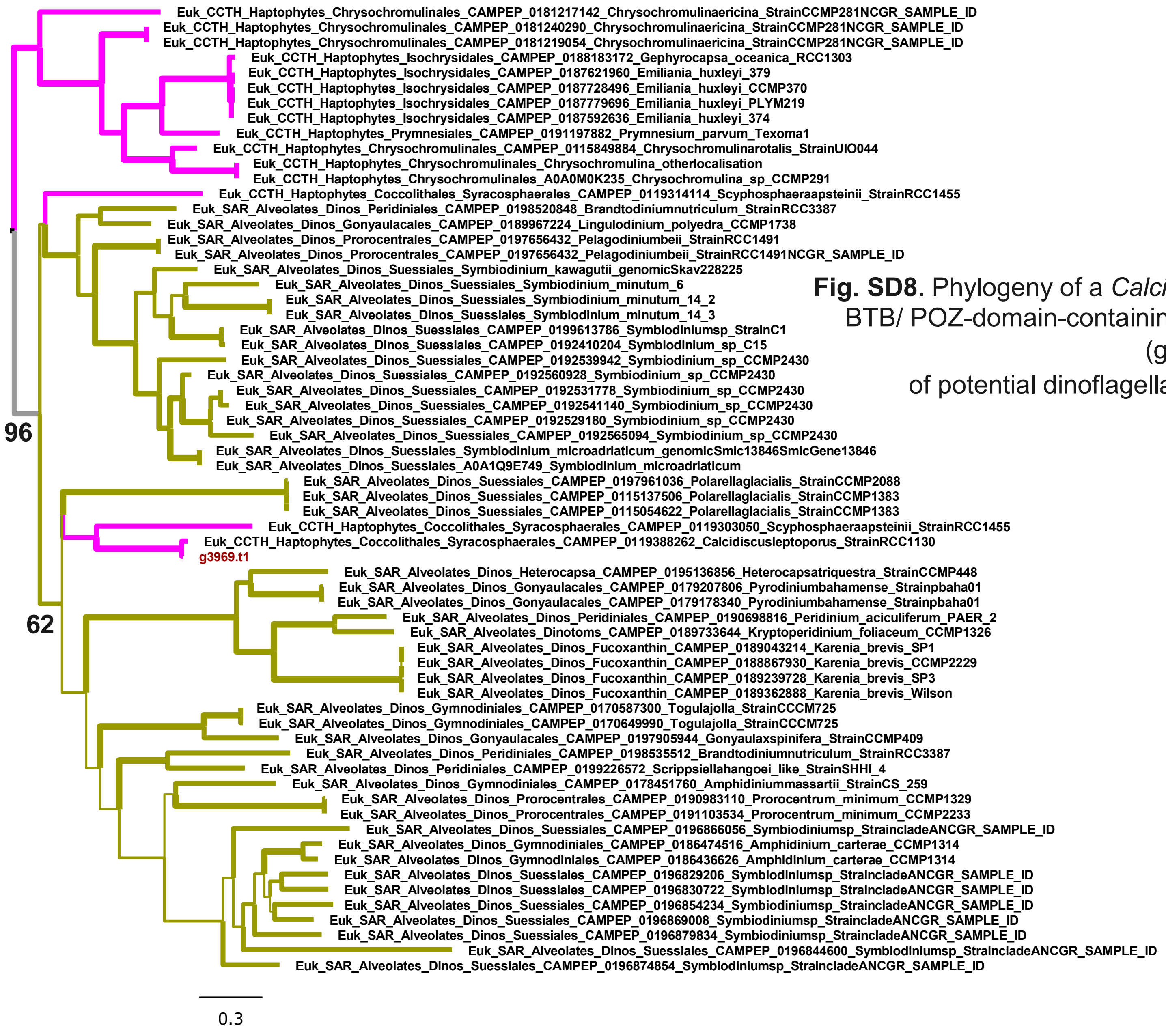

Fig. SD8. Phylogeny of a *Calcidiscus* BTB/ POZ-domain-containing protein (g3969.t1), of potential dinoflagellate origin.

Fig. SD9. Phylogeny of a *Calcidscus* xylulose reductase/ enoyl-ACP reductase protein (g10292.t1), of potential dinoflagellate origin.

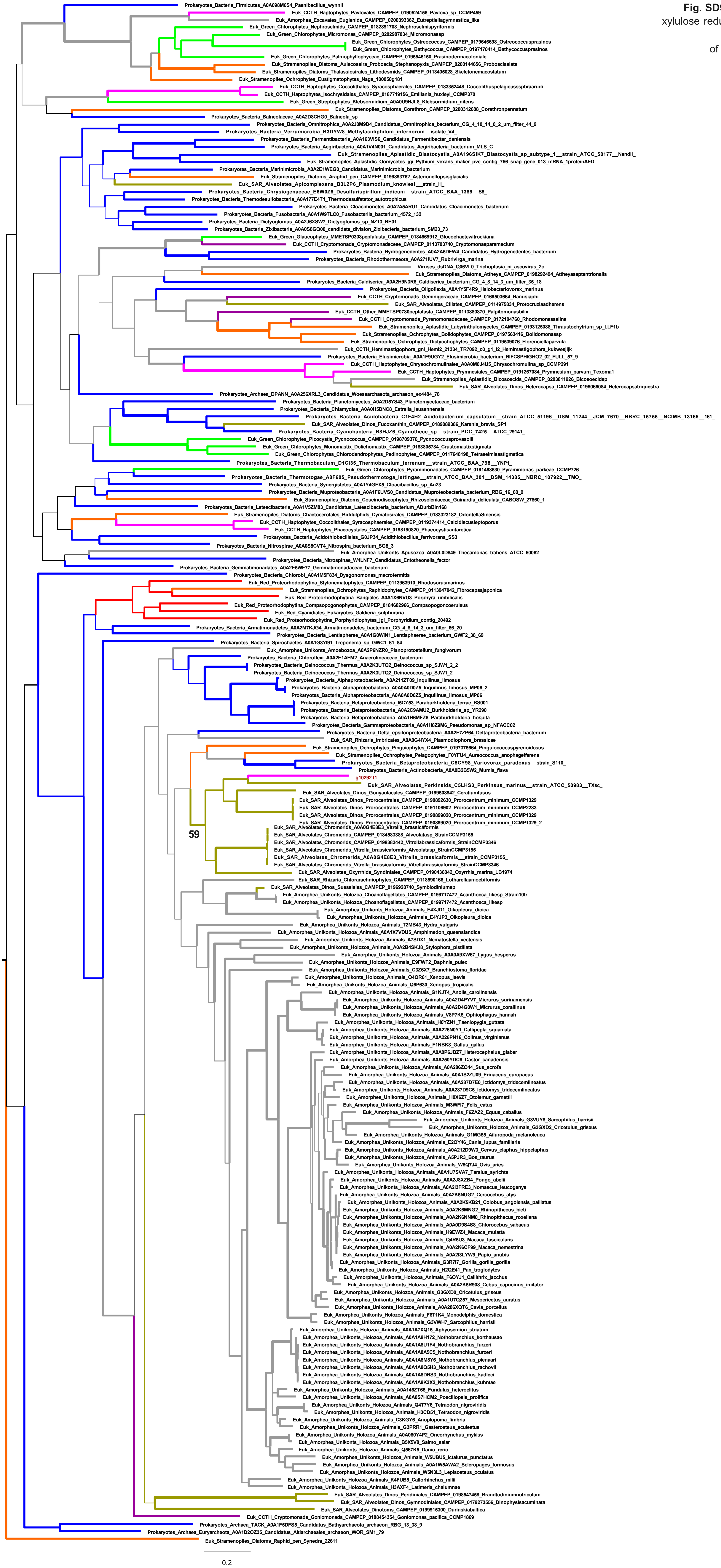

Fig. SD10. Phylogeny of a *Calcidiscus* DSBA-like thioredoxin protein (g2453.t1), of potential dinoflagellate origin.

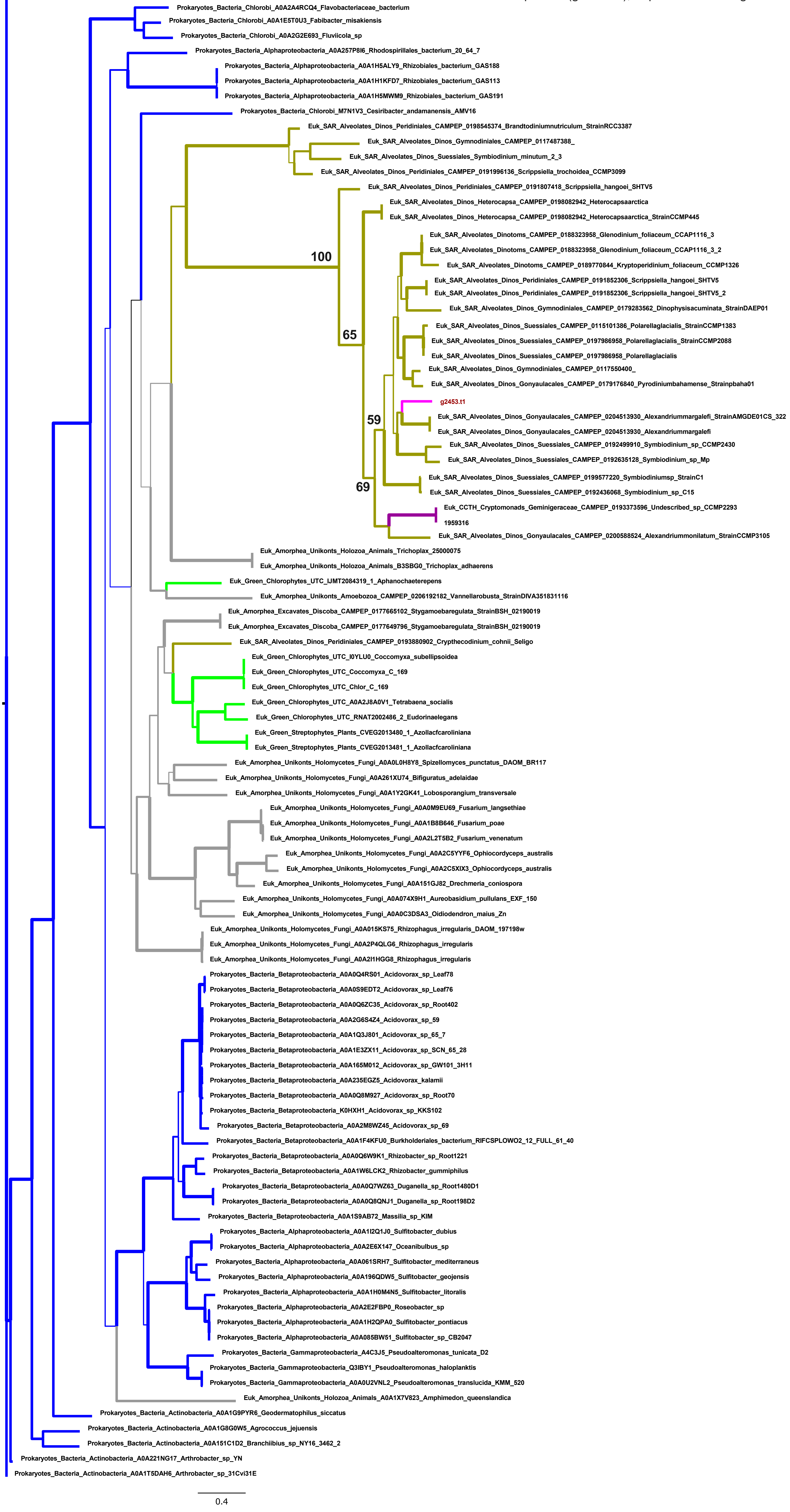

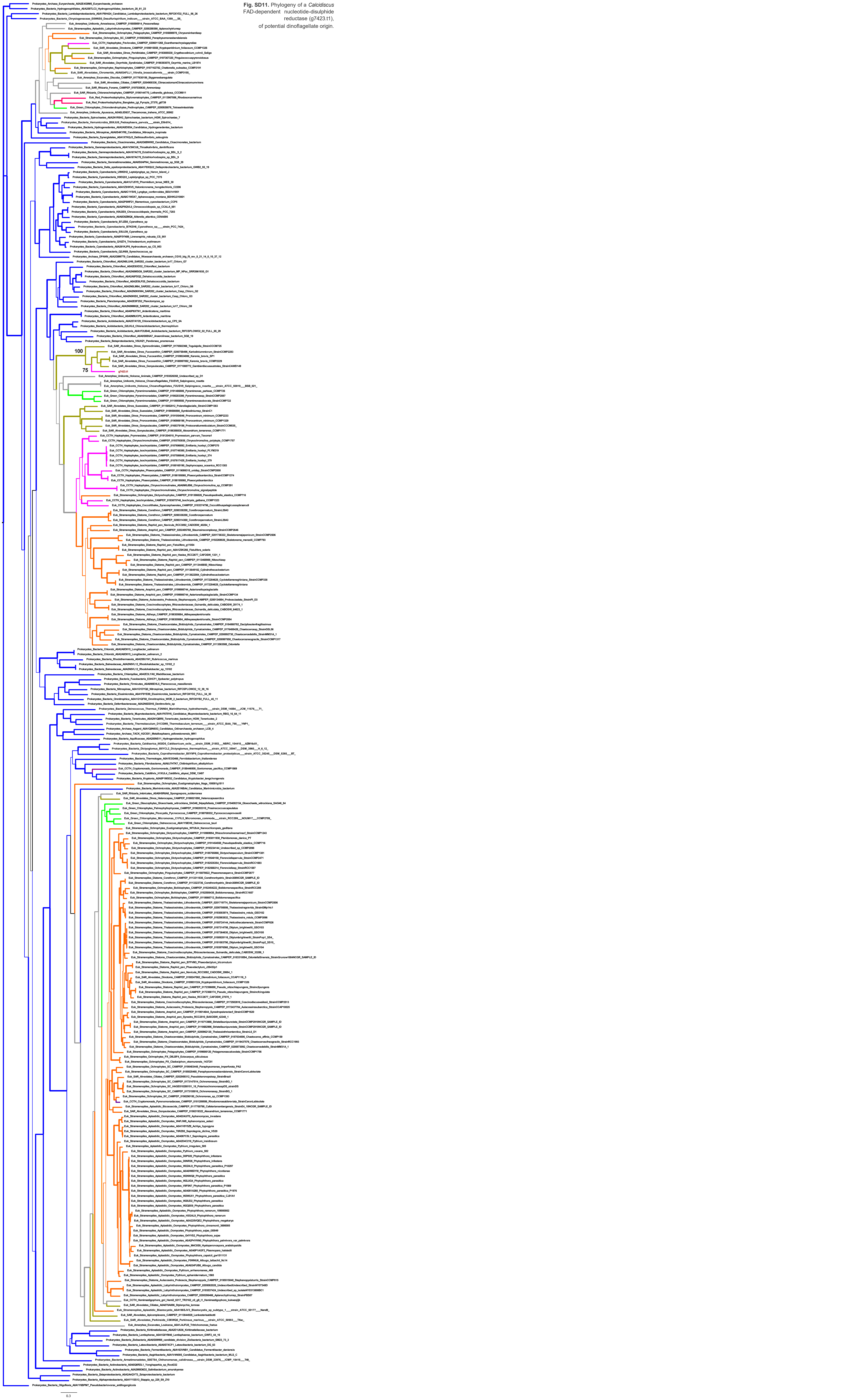

Fig. SD11. Phylogeny of a *Calcidiscus* FAD-dependent nucleotide-disulphide reductase (g7423.t1), of potential dinoflagellate origin.

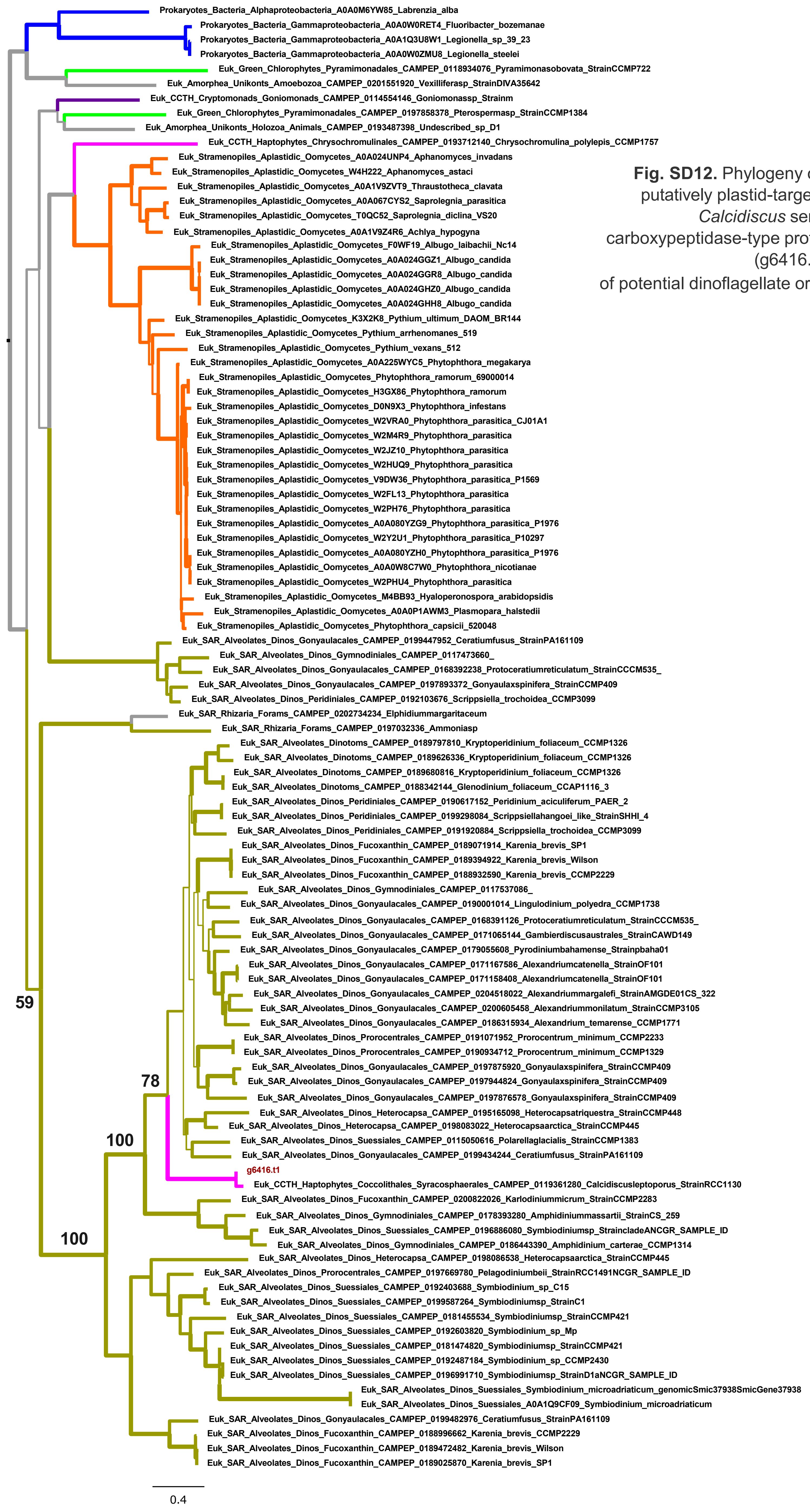

Fig. SD12. Phylogeny of a putatively plastid-targeted *Calcidiscus* serine carboxypeptidase-type protein (g6416.t1), of potential dinoflagellate origin.

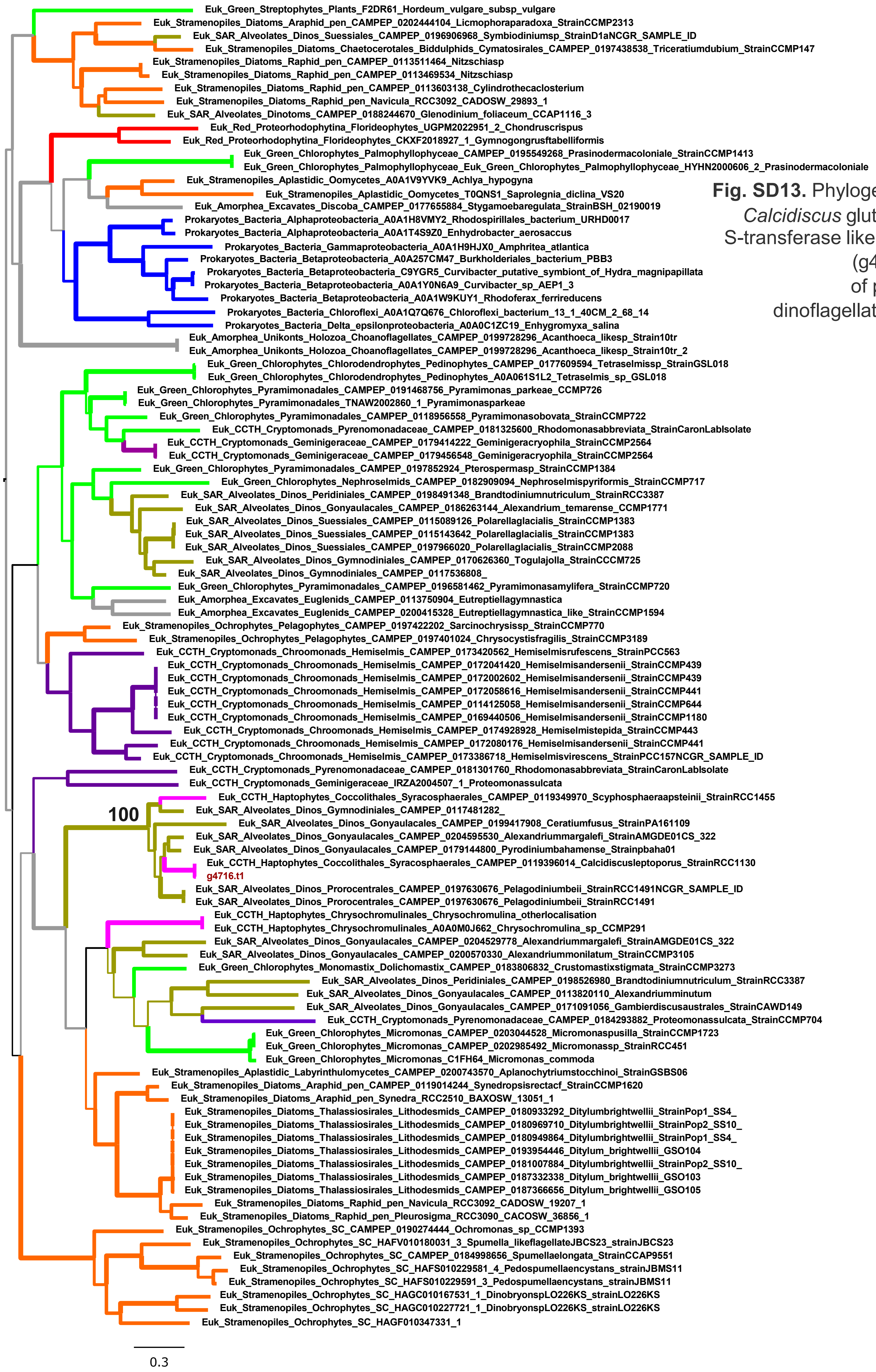

Fig. SD13. Phylogeny of a *Calcidiscus* glutathione S-transferase like protein (g4716.t1) of potential dinoflagellate origin.

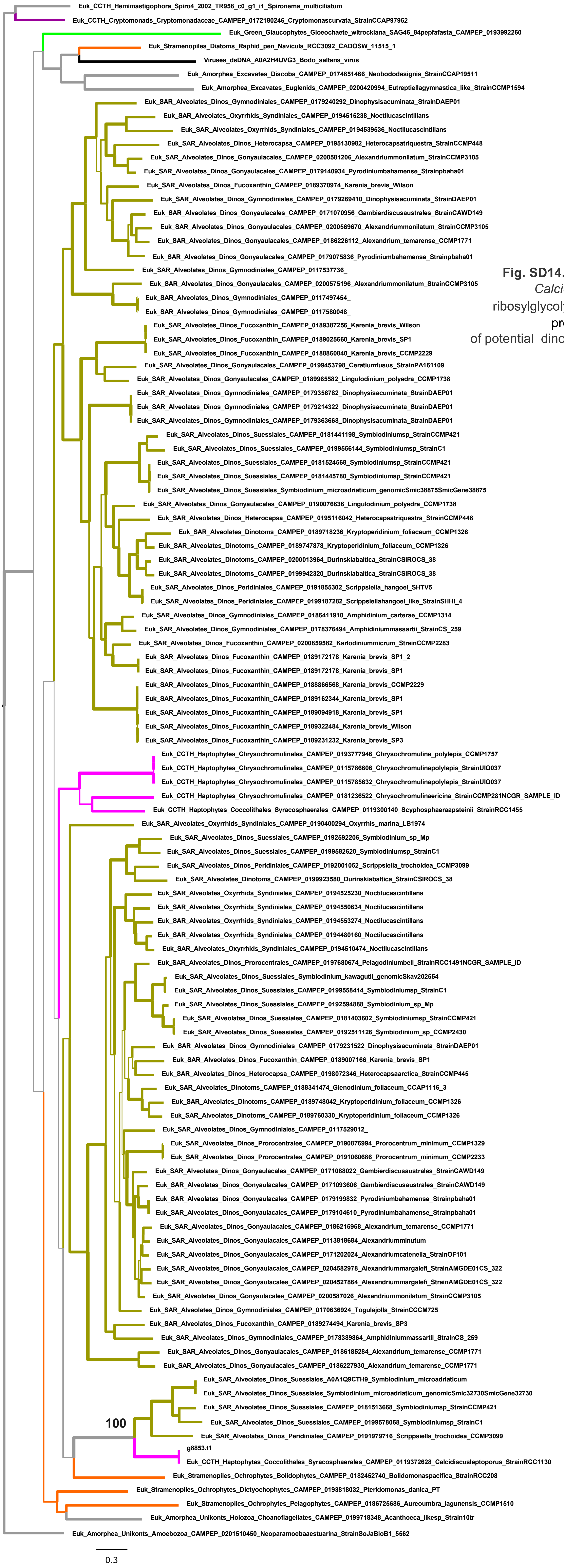

Fig. SD14. Phylogeny of a *Calcidiscus* tri1/ ADP ribosylglycolylhydrolase-like protein (g8853.t1) of potential dinoflagellate origin.

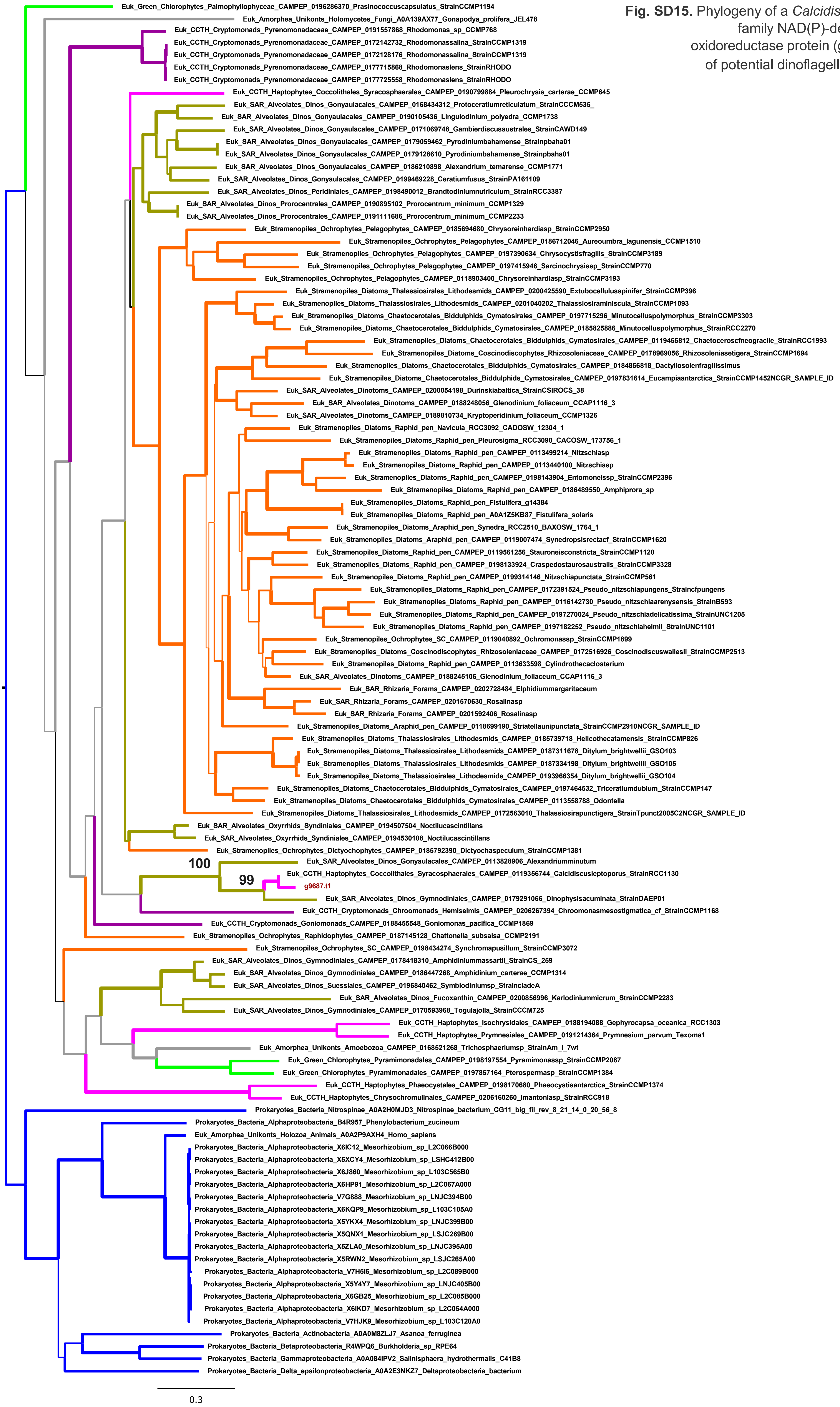

Fig. SD15. Phylogeny of a *Calcidiscus* SDR family NAD(P)-dependent oxidoreductase protein (g9687.t1) of potential dinoflagellate origin.

Fig. S16. Phylogeny of a putative mitochondria-targeted *Calcidiscus* TPA: enoyl-CoA hydratase/isomerase (g2480.t1), of potential Bolidophyte origin.

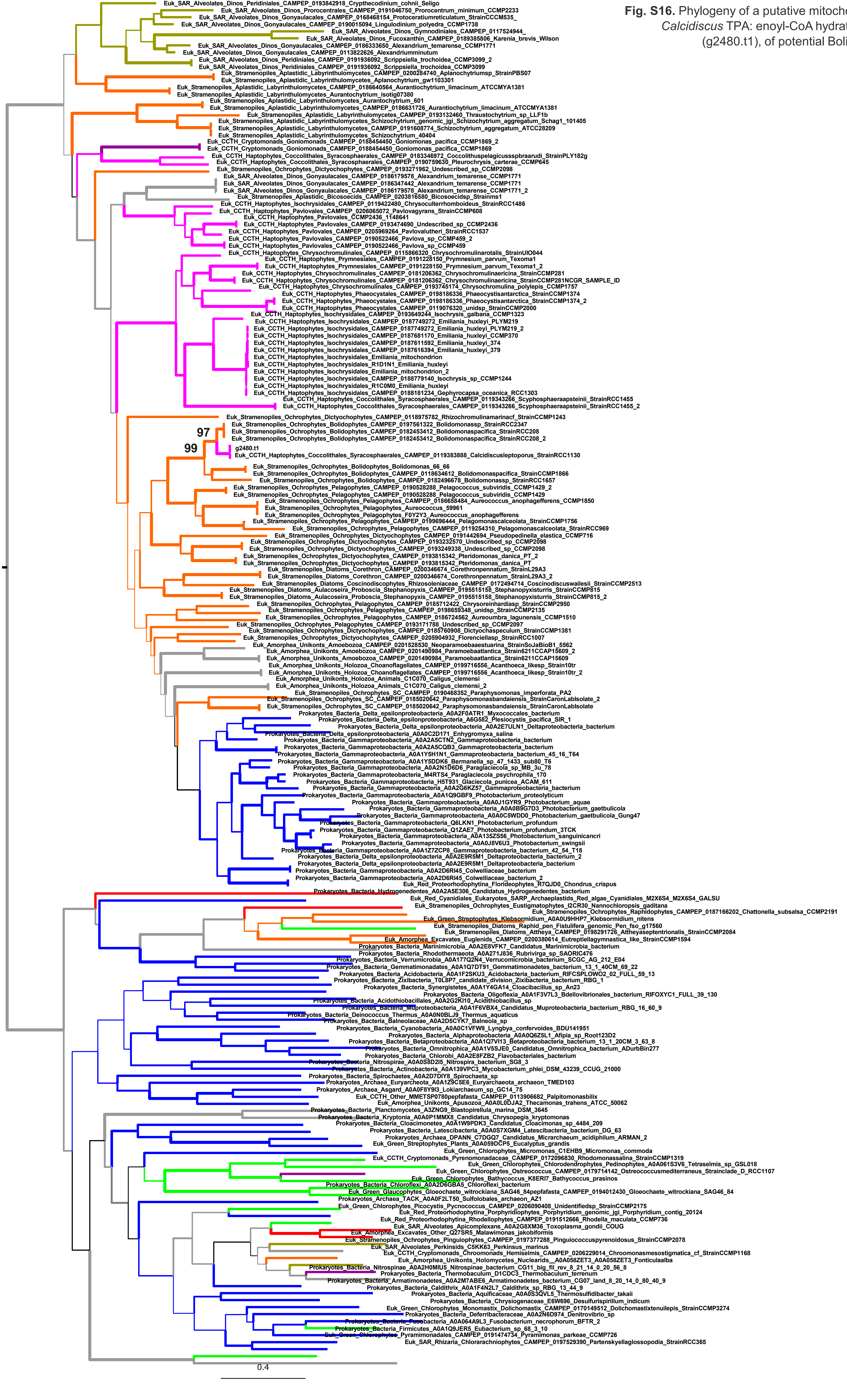

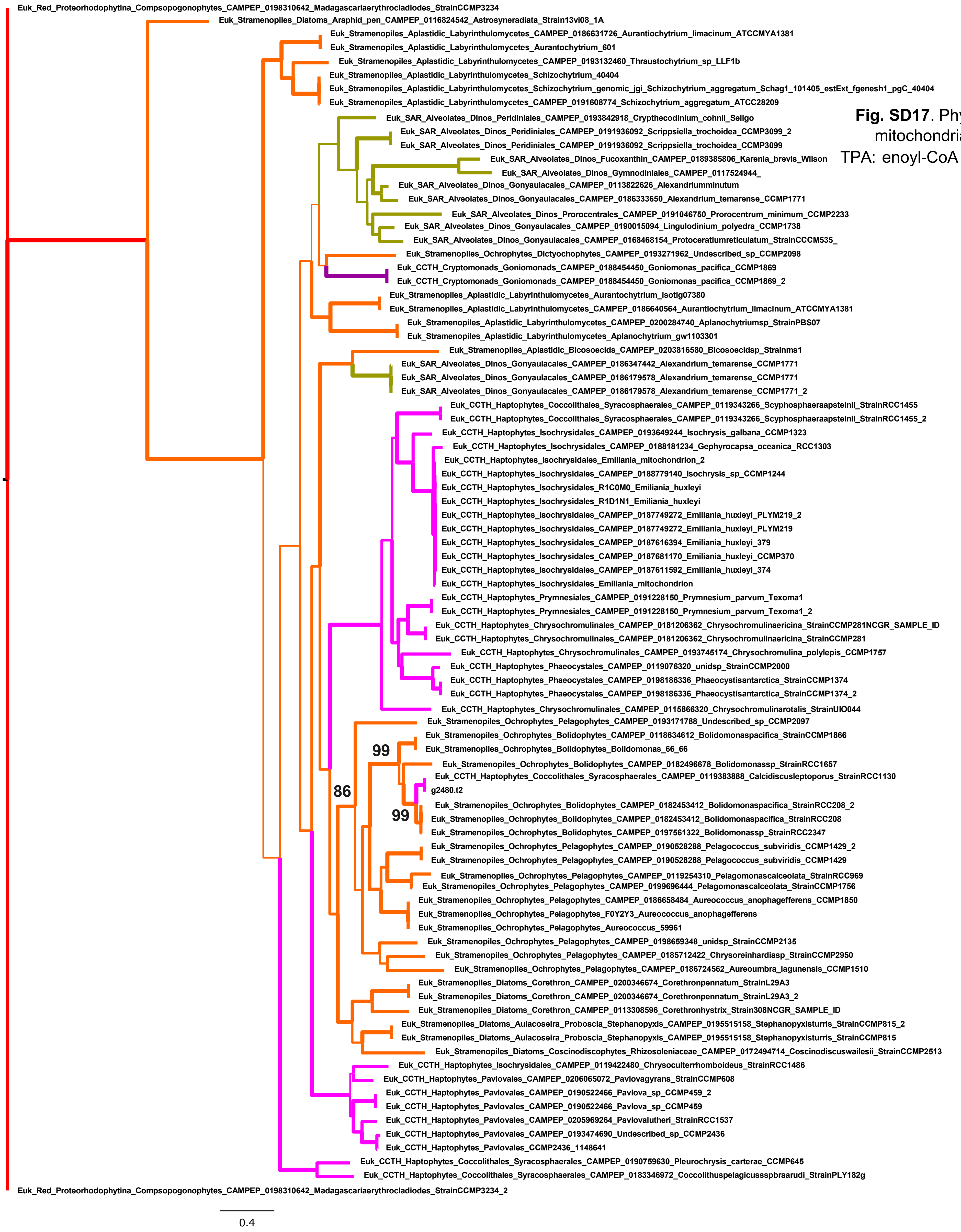

Fig. SD17. Phylogeny of a putatively mitochondria-targeted *Calcidiscus* TPA: enoyl-CoA hydratase/isomerase (g2480.t2), of potential Bolidophyte origin.

Fig. SD18. Phylogeny of a putatively endomembrane/ secreted *Calcidiscus* protein (g2560.t1), of potential stramenopile origin.

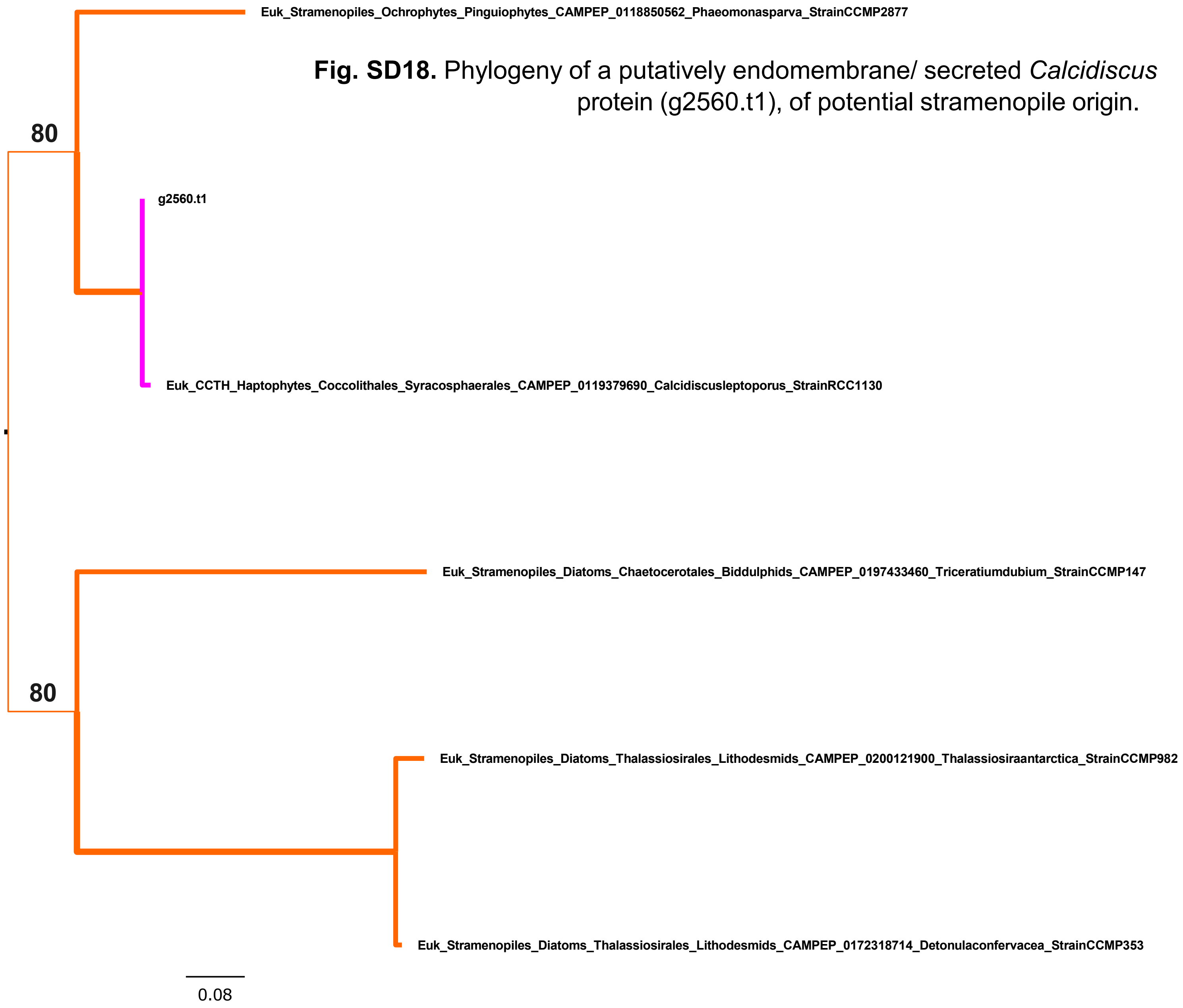

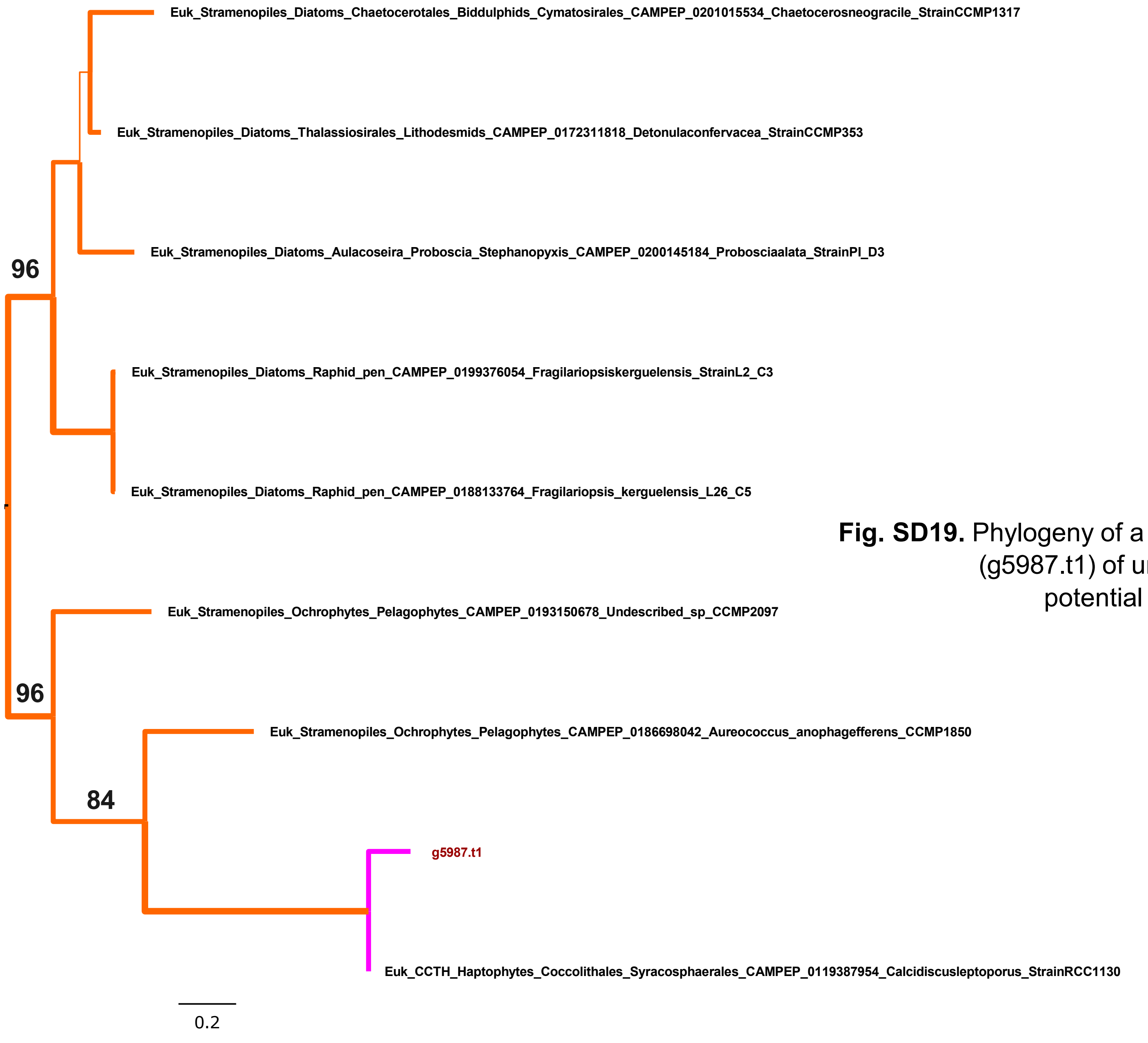

Fig. SD19. Phylogeny of a *Calcidiscus* protein (g5987.t1) of unknown function, of potential Pelagophyte origin.

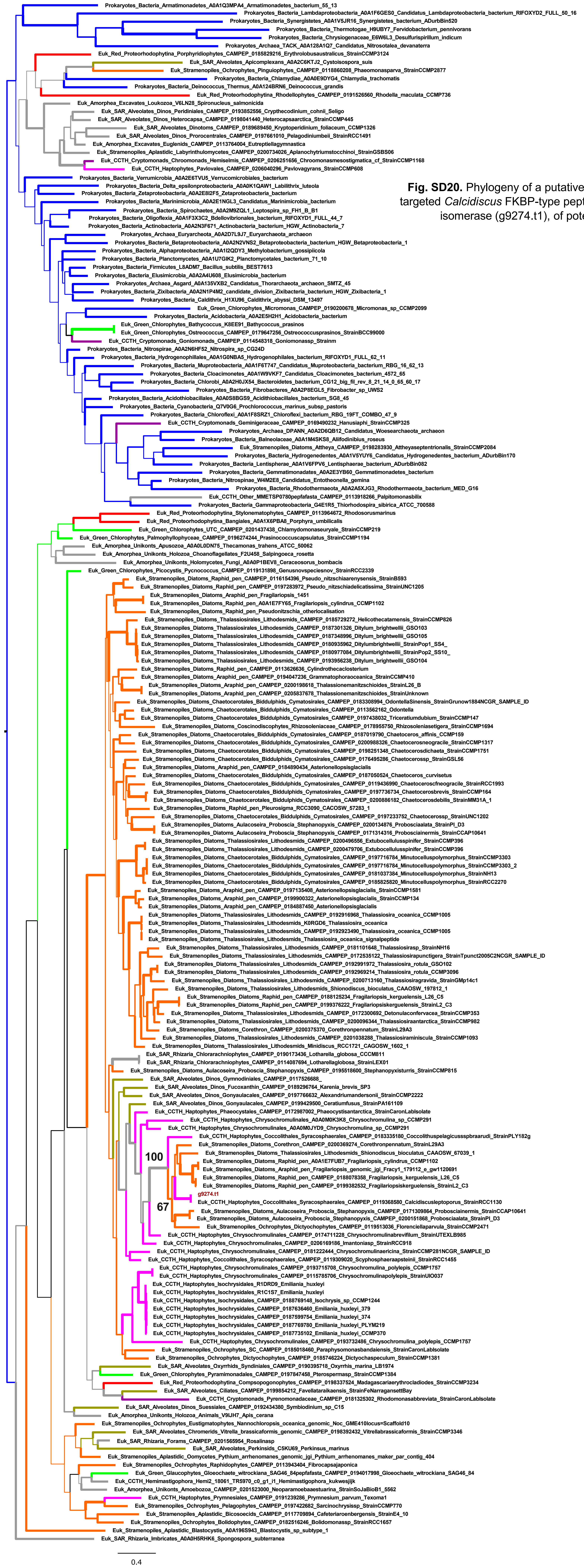

Fig. SD20. Phylogeny of a putatively endomembrane-targeted *Calcidiscus* FKBP-type peptidyl-prolyl cis-trans isomerase (g9274.t1), of potential diatom origin.

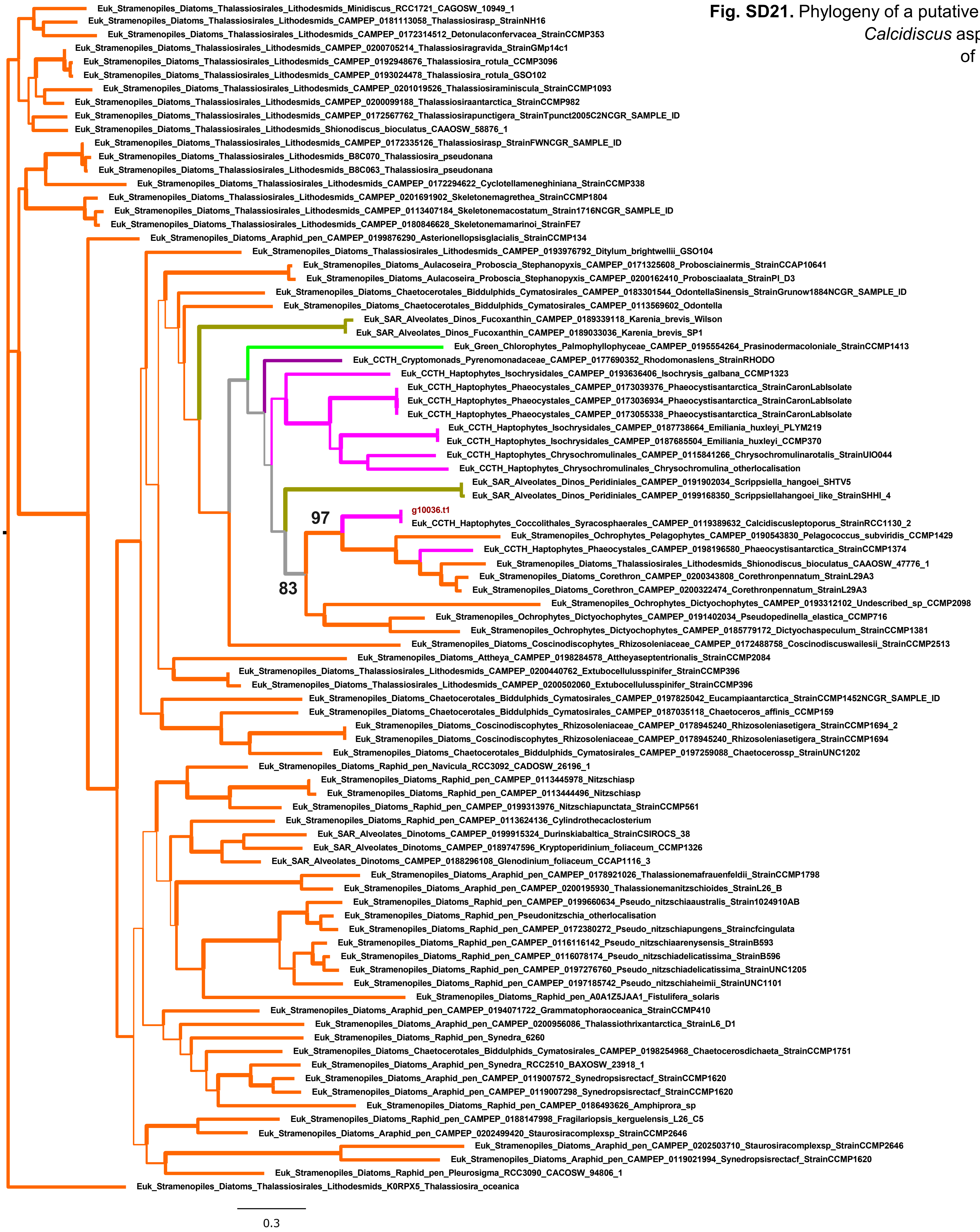

Fig. SD21. Phylogeny of a putatively endomembrane-targeted *Calcidiscus* aspartyl protease (g10036.t1), of potential Ochrophyte origin.

Fig. SD22. Phylogeny of a putatively mitochondria-targeted *Calcidiscus* aspartyl protease (g10036.t2), of potential Ochrophyte origin.

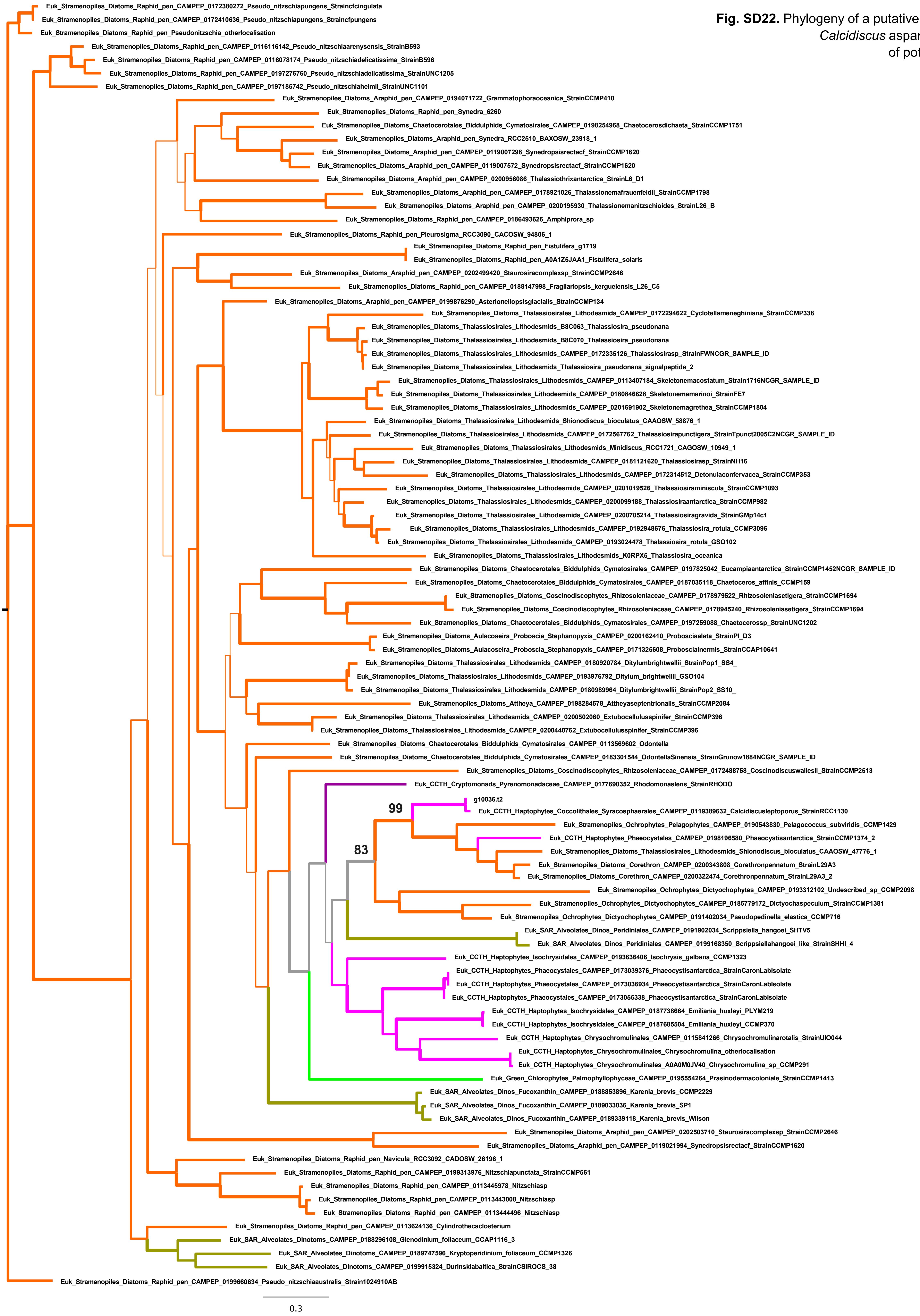

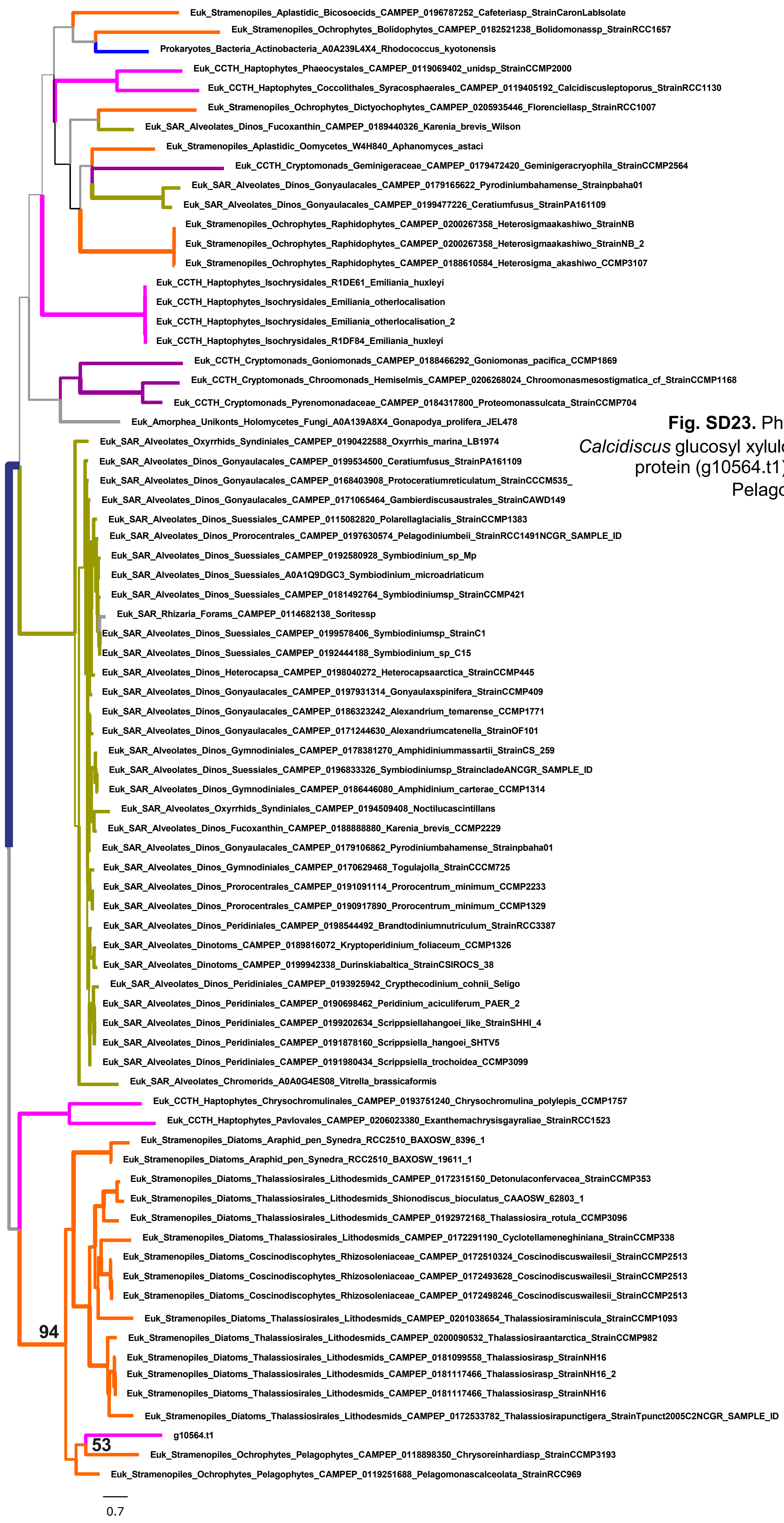

Fig. SD23. Phylogeny of a *Calcidiscus* glucosyl xylulotransferase protein (g10564.t1), of potential Pelagophyte origin.
