# Supplementary material for: The Calcidiscus leptoporus genome reveals vitamin-mediated holobiont interactions
Source: ISME Commun. 2026 Aug 23;6(1):ycag222. doi: 10.1093/ismeco/ycag222 (PMC13518057; doi:10.1093/ismeco/ycag222)
Supplement: Supplementary file 1 [file supplementary_material_ycag222.zip › Suppl Dataset SD3.pdf]

Supplementary Dataset 3. Whole proteome assessment of bacterial MAGs

Protein sequences were predicted for the six bacterial genomes assembled in this study.

SD24 to SD30

For reference genomes, protein sequences were downloaded from NCBI: *Balneola vulgaris*: DSM_17893; *Gracilimonas amylolytica*: GCF_002911695.1*; Marinobacter adhaerens* HP15: GCF_000166295.1; *Marinobacter goseongensis*: GCA_023156235.1; *Marinobacter lipolyticus* SM19: GCF_000397065.2; *Marivita cryptomonadis* : GCF_002115725.1*; Roseovarius lutimaris*: GCF_900115165.1; *Roseovarius marisflavi*: GCF_900142625.1; *Pseudosulfitobacter pseudonitzschiae*: GCF_000712315.1. Proteomes were compared (circle plots) and annotated with KEGGS Orthology numbers (KOs) using DRAM (https://github.com/WrightonLabCSU/DRAM) and upset diagrams generated using ggVennDiagram (https://github.com/gaospecial/ggVennDiagram).

In SD26 to SD30, shown are (A) circle plots of protein similarity and (B) upset diagrams between MAGs and closest relatives.

SD24

Table 1. Genome statistics and KO annotation summary for bacterial MAGs and reference genomes. Columns show genome identifier, contig count, total genome length (Mb), predicted gene count, total KEGG Orthology (KO) assignments, and number of distinct KOs. Reference genomes were included for comparison with the reconstructed bacterial MAGs.

Genome	Contig count	Total length (Mb)	Gene count	KO count	Distinct KO count
MAG_01	1	4.4	4222	2403	1928
MAG_02	1	4.1	3867	2240	1828
MAG_03	1	3.8	3220	1415	1116
MAG_04	2	3.9	3553	2115	1682
MAG_05	2	3.6	3342	1987	1613
MAG_06	3	4.0	3623	2022	1584
MAG_101	1	0.028	40	9	10
MAG_102	1	0.017	26	8	9
MAG_103	40	3.6	304	1453	1222
MAG_104	1	0.66	675	289	183
MAG_105	26	1.8	262	715	614
Balneola_vulgaris	18	2.9	2436	1223	1044
Gracilimonas_amylolytica	51	3.2	2672	1373	1102
Marinobacter_adhaerens HP15	3	4.7	4197	2452	1950
Marinobacter_goseongensis	88	4.0	3605	2148	1786
Marinobacter_lipolyticus SM19	57	4.0	3573	2255	1875
Marivita_cryptomonadis	115	4.6	4384	2408	1778
Roseovarius_lutimaris	73	4.3	4059	2241	1727
Roseovarius_marisflavi	83	4.1	3837	2102	1651
Pseudosulfitobacter pseudonitzschiae	80	4.9	4750	2596	1854

SD25

Whole proteome assessment of KO in bacterial MAGs.
Upset diagram between MAGs and each of the closest relative. Coloured boxes indicate pairs of related species.

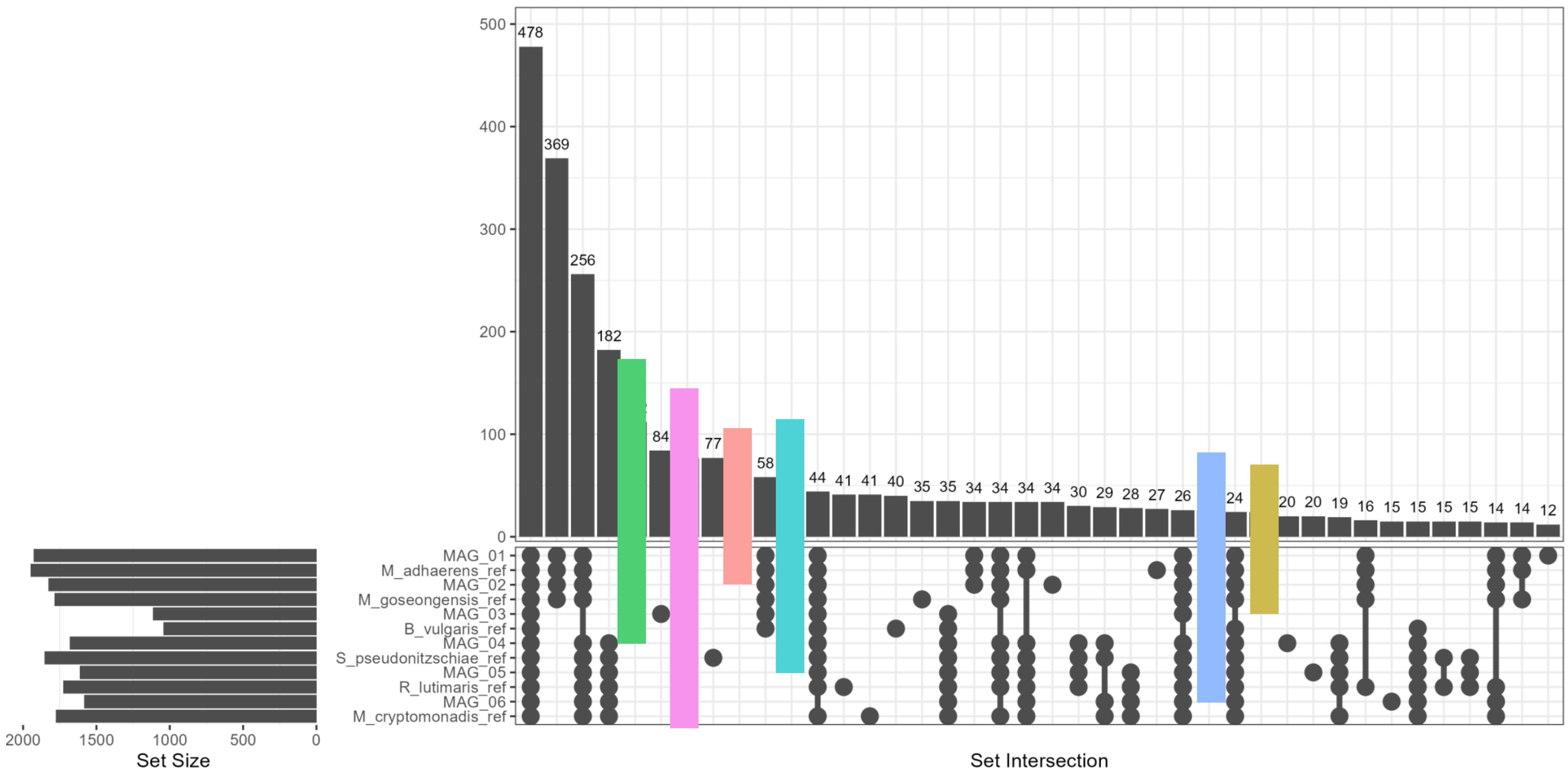

SD26

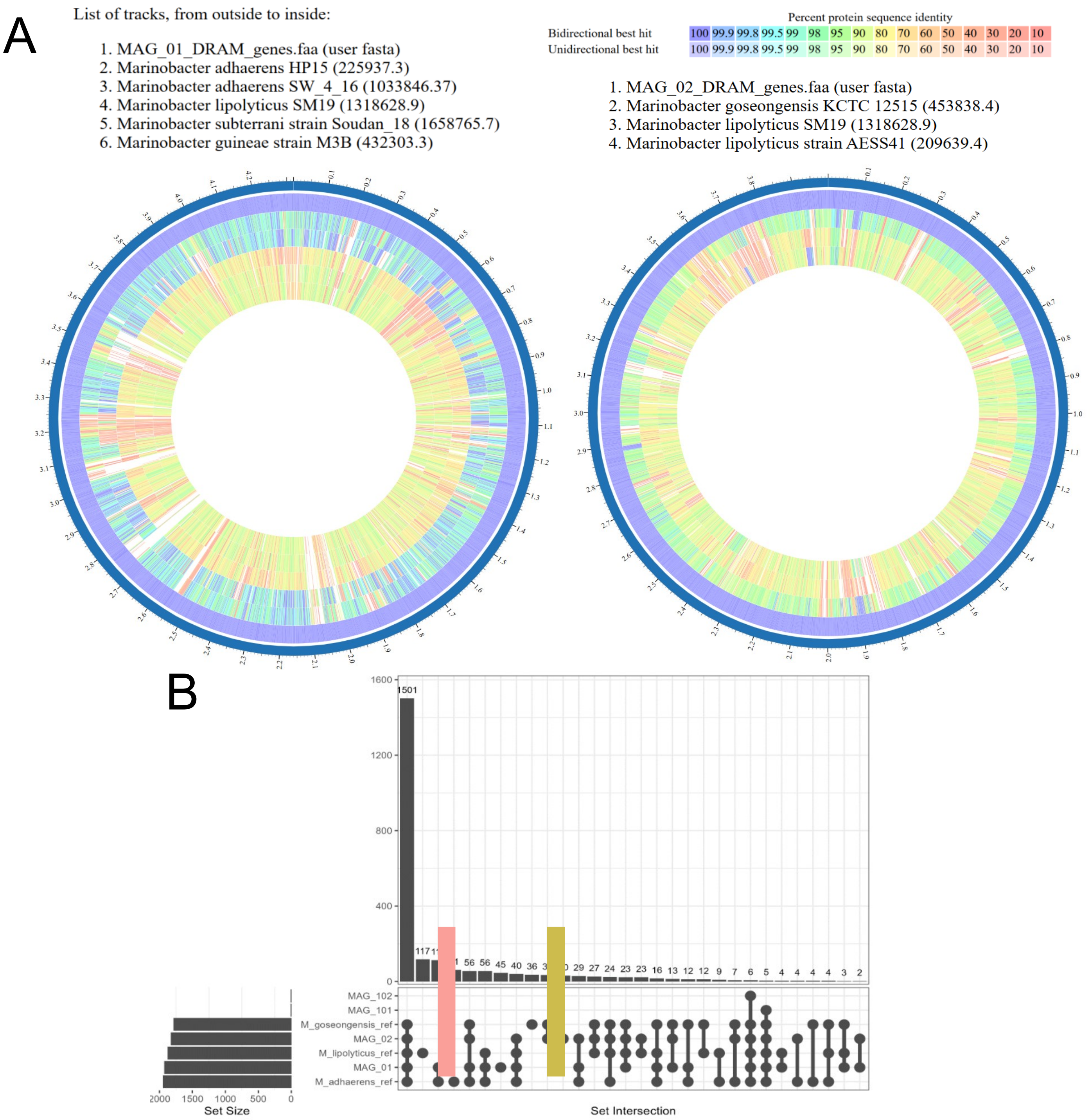
A
List of tracks, from outside to inside:
1. MAG_01_DRAM_genes.faa (user fasta)
2. Marinobacter adhaerens HP15 (225937.3)
3. Marinobacter adhaerens SW_4_16 (1033846.37)
4. Marinobacter lipolyticus SM19 (1318628.9)
5. Marinobacter subterrani strain Soudan_18 (1658765.7)
6. Marinobacter guineae strain M3B (432303.3)
Percent protein sequence identity
Bidirectional best hit
Unidirectional best hit
100 99.9 99.8 99.5 99 98 95 90 80 70 60 50 40 30 20 10
1. MAG_02_DRAM_genes.faa (user fasta)
2. Marinobacter goseongensis KCTC 12515 (453838.4)
3. Marinobacter lipolyticus SM19 (1318628.9)
4. Marinobacter lipolyticus strain AESS41 (209639.4)
B
1600
1200
800
400
0
1501
117
56 56 45 40 36
29 27 24 23 23 16 13 12 12 9 7 6 5 4 4 4 4 3 2
MAG_102
MAG_101
M_goseongensis_ref
MAG_02
M_lipolyticus_ref
MAG_01
M_adhaerens_ref
2000 1500 1000 500 0
Set Size
Set Intersection

SD27

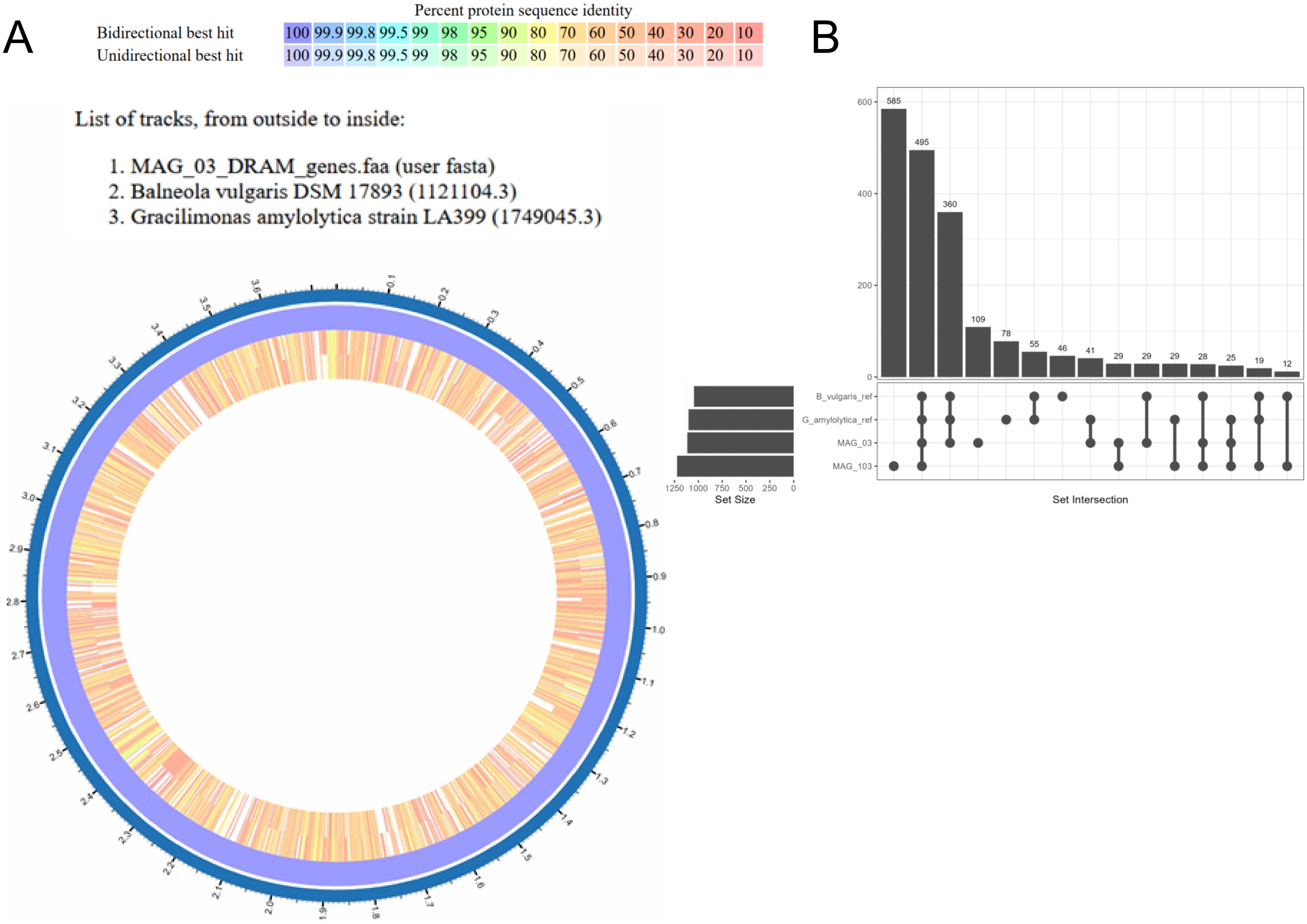
A
Percent protein sequence identity
Bidirectional best hit 100 99.9 99.8 99.5 99 98 95 90 80 70 60 50 40 30 20 10
Unidirectional best hit 100 99.9 99.8 99.5 99 98 95 90 80 70 60 50 40 30 20 10
List of tracks, from outside to inside:
1. MAG_03_DRAM_genes.faa (user fasta)
2. Balneola vulgaris DSM 17893 (1121104.3)
3. Gracilimonas amylolytica strain LA399 (1749045.3)
B
585
495
360
109
78
55
46
41
29
29
29
28
25
19
12
B_vulgaris_ref
G_amylolytica_ref
MAG_03
MAG_103
1250 1000 750 500 250 0
Set Size
Set Intersection

SD28

A

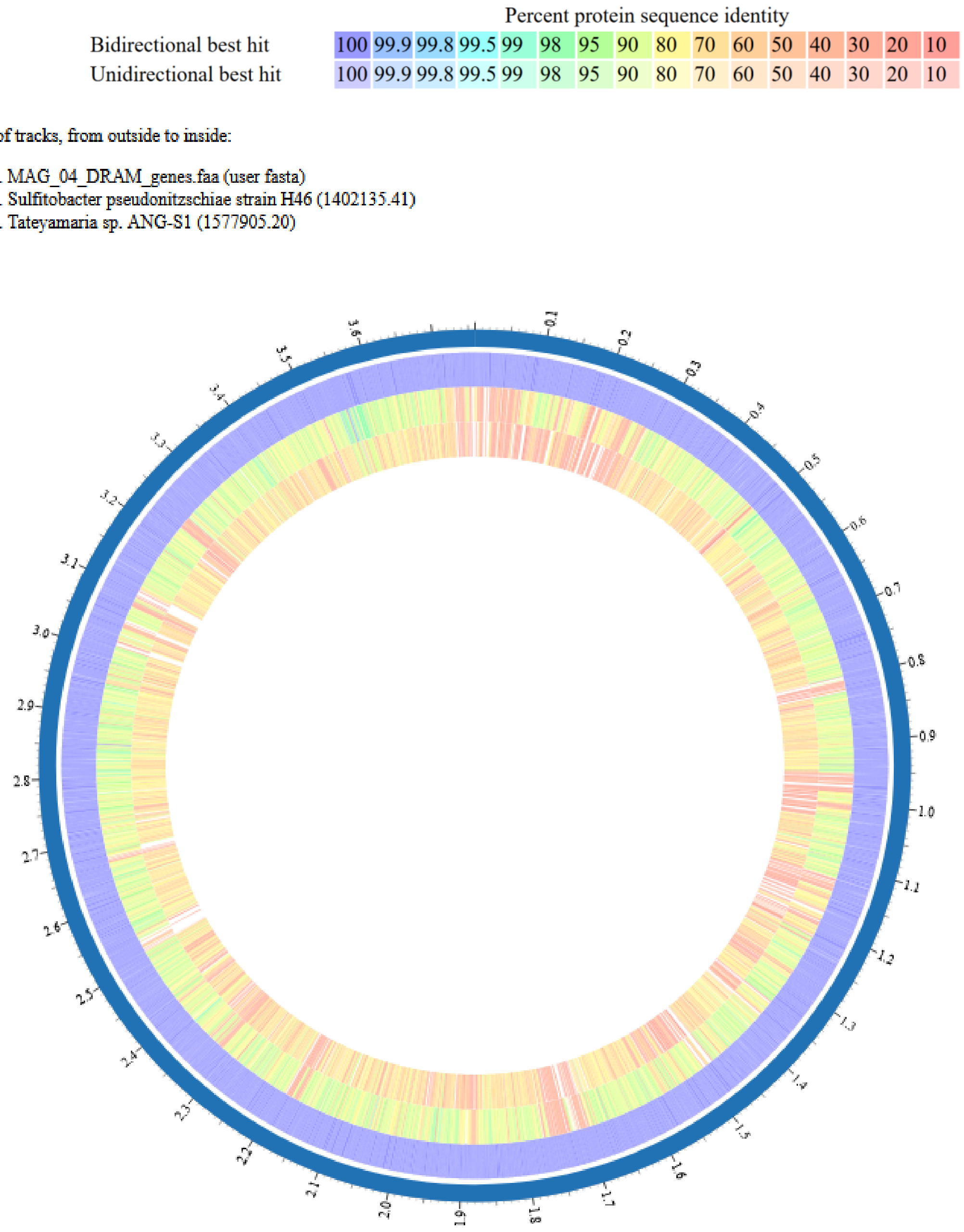

Percent protein sequence identity
Bidirectional best hit
Unidirectional best hit
100 99.9 99.8 99.5 99 98 95 90 80 70 60 50 40 30 20 10
100 99.9 99.8 99.5 99 98 95 90 80 70 60 50 40 30 20 10
List of tracks, from outside to inside:
1. MAG_04_DRAM_genes.faa (user fasta)
2. Sulfitobacter pseudonitzschiae strain H46 (1402135.41)
3. Tateyamaria sp. ANG-S1 (1577905.20)

B

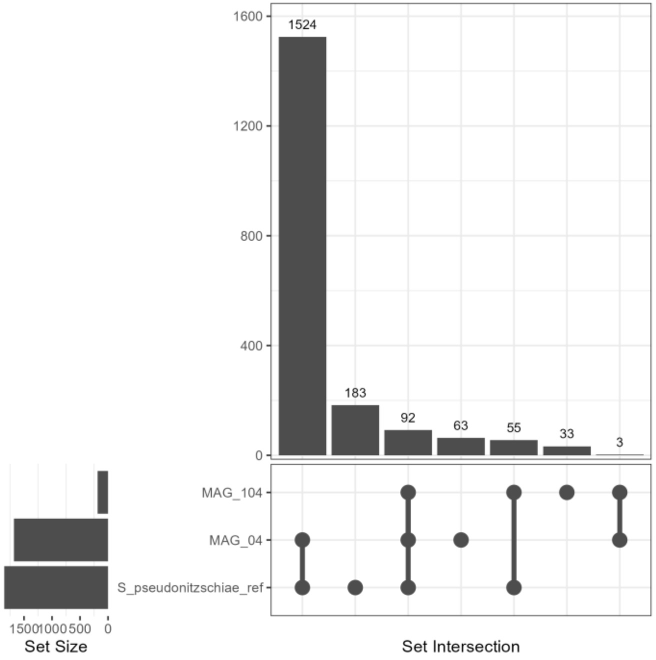

1600
1200
800
400
0
1524
183
92
63
55
33
3
MAG_104
MAG_04
S_pseudonitzschiae_ref
1500 1000 500 0
Set Size
Set Intersection

SD29

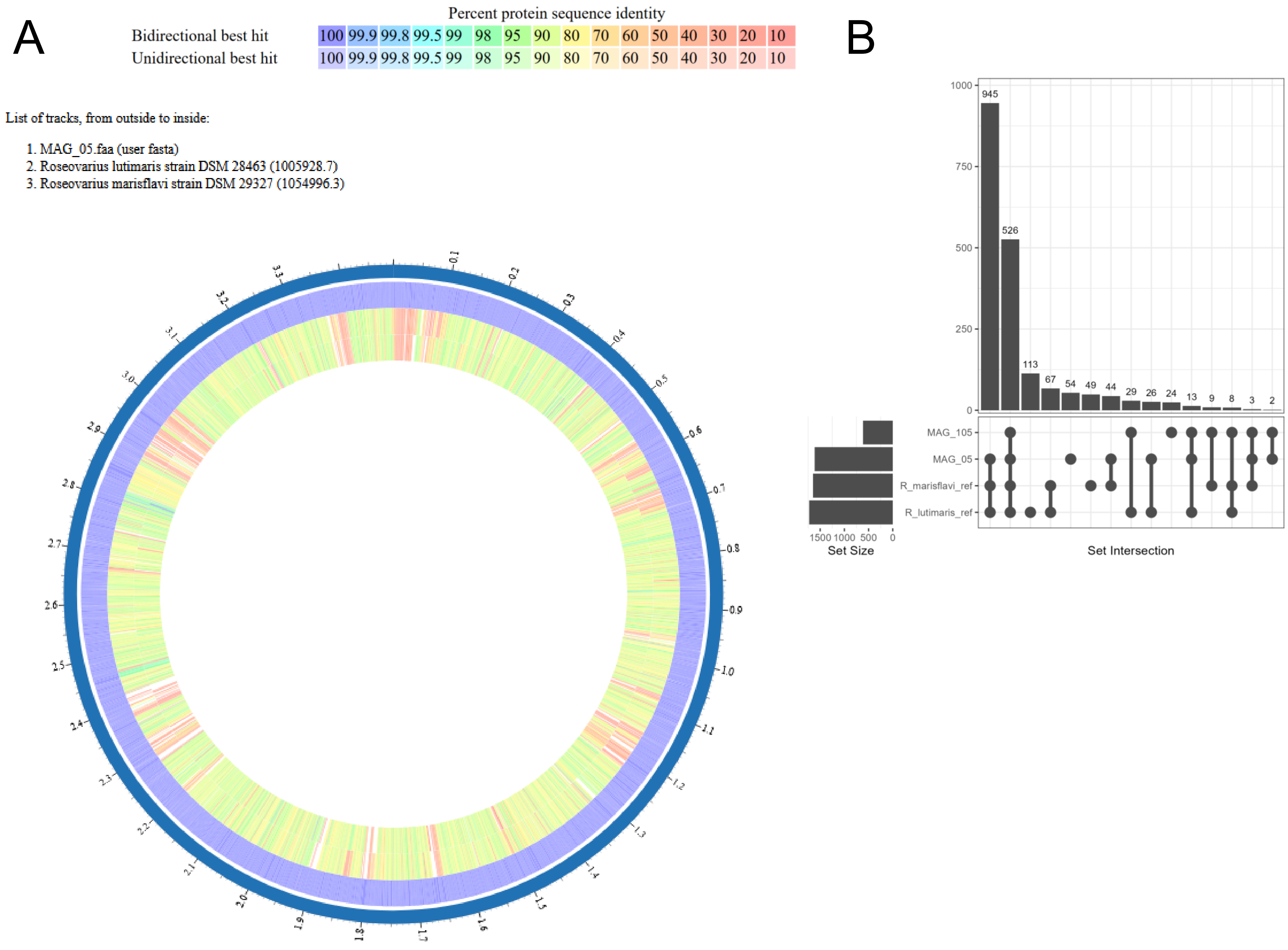
A
Percent protein sequence identity
Bidirectional best hit
Unidirectional best hit
100 99.9 99.8 99.5 99 98 95 90 80 70 60 50 40 30 20 10
List of tracks, from outside to inside:
1. MAG_05.faa (user fasta)
2. Roseovarius lutimaris strain DSM 28463 (1005928.7)
3. Roseovarius marisflavi strain DSM 29327 (1054996.3)
B
945
526
113
67
54
49
44
29
26
24
13
9
8
3
2
1000
750
500
250
0
MAG_105
MAG_05
R_marisflavi_ref
R_lutimaris_ref
1500 1000 500 0
Set Size
Set Intersection

SD30

A

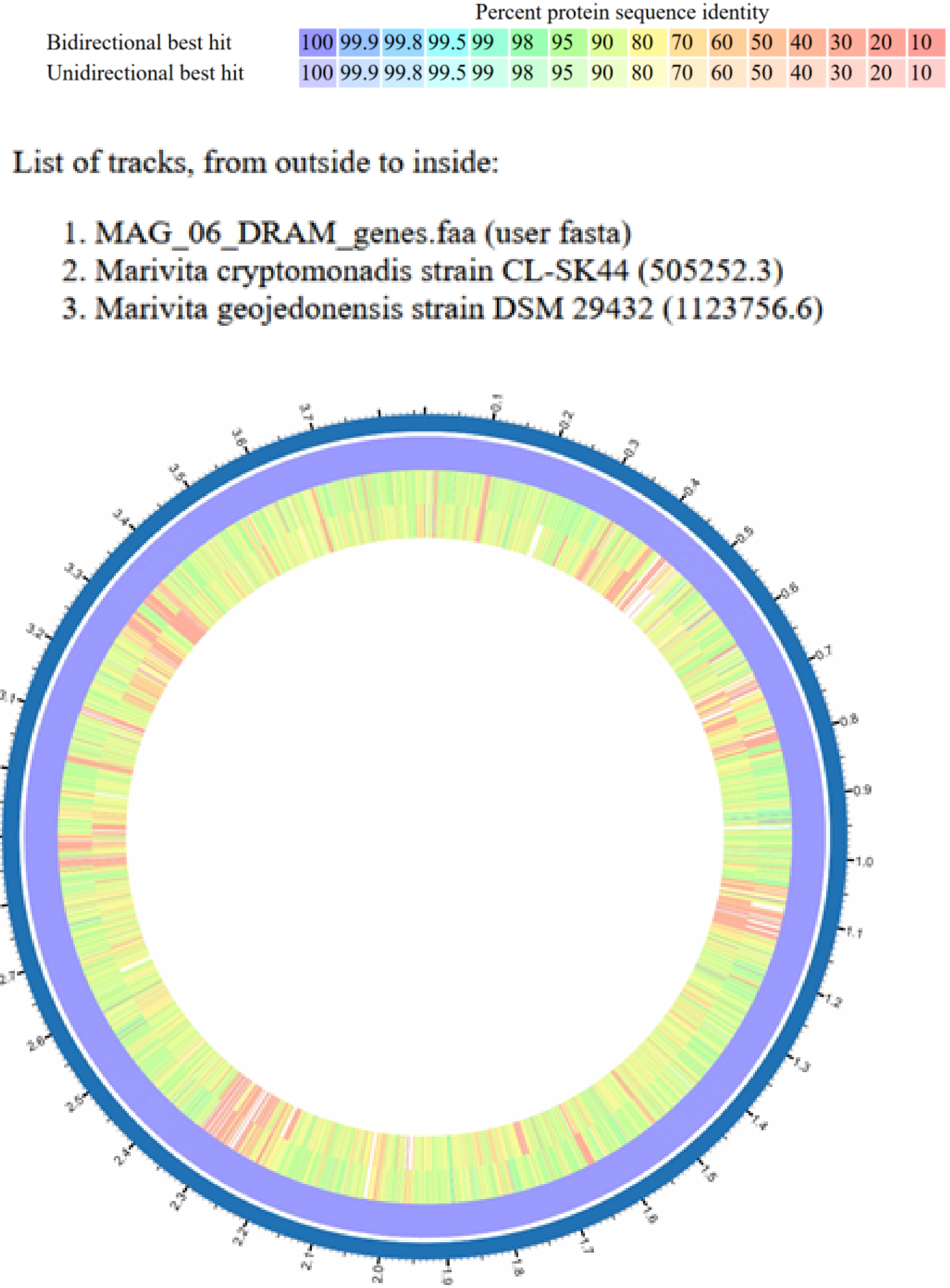

Percent protein sequence identity
Bidirectional best hit
Unidirectional best hit
100 99.9 99.8 99.5 99 98 95 90 80 70 60 50 40 30 20 10
100 99.9 99.8 99.5 99 98 95 90 80 70 60 50 40 30 20 10
List of tracks, from outside to inside:
1. MAG_06_DRAM_genes.faa (user fasta)
2. Marivita cryptomonadis strain CL-SK44 (505252.3)
3. Marivita geojedonensis strain DSM 29432 (1123756.6)

B

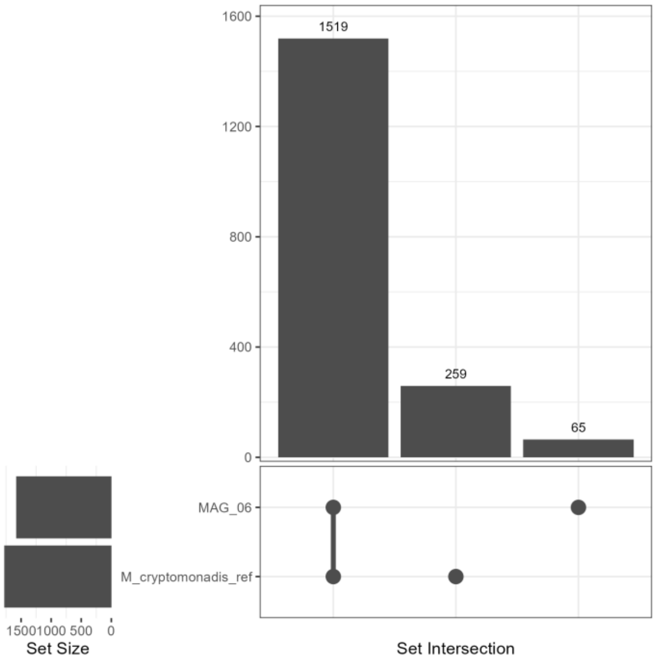

1600
1200
800
400
0
1519
259
65
MAG_06
M_cryptomonadis_ref
1500
1000
500
0
Set Size
Set Intersection
